# Importance of rare gene copy number alterations for personalized tumor characterization and survival analysis

**DOI:** 10.1186/s13059-016-1058-1

**Published:** 2016-10-03

**Authors:** Michael Seifert, Betty Friedrich, Andreas Beyer

**Affiliations:** 1 Carl Gustav Carus Faculty of Medicine, Technische Universität Dresden, Institute for Medical Informatics and Biometry, Fetscherstr. 74, Dresden, 01307 Germany; 2 National Center for Tumor Diseases (NCT), Dresden, Germany; 3 Institute of Molecular Systems Biology, Auguste-Piccard-Hof 1, Zurich, 8093 Switzerland; 4 Cellular Networks and Systems Biology, CECAD, University of Cologne, Joseph-Stelzmann-Str. 26, Cologne, 50931 Germany

**Keywords:** Cancer genomics, Bioinformatics, Computational systems biology, Network biology, Network inference, Network propagation, Gene copy number mutations

## Abstract

It has proven exceedingly difficult to ascertain rare copy number alterations (CNAs) that may have strong effects in individual tumors. We show that a regulatory network inferred from gene expression and gene copy number data of 768 human cancer cell lines can be used to quantify the impact of patient-specific CNAs on survival signature genes. A focused analysis of tumors from six tissues reveals that rare patient-specific gene CNAs often have stronger effects on signature genes than frequent gene CNAs. Further comparison to a related network-based approach shows that the integration of indirectly acting gene CNAs significantly improves the survival analysis.

**Electronic supplementary material:**

The online version of this article (doi:10.1186/s13059-016-1058-1) contains supplementary material, which is available to authorized users.

## Background

Tumor cells harbor combinations of mutations that together impair molecular pathways, which results in neoplastic transformation. Although only a relatively small fraction of all mutations in any given cancer cell contributes to tumorigenesis, it is emerging that many more genes than previously thought determine clinically relevant endpoints such as proliferation rates, metastatic potential, or drug resistance [1, 2]. Clearly, hundreds of genes have the potential to contribute to tumor phenotypes [3], but we are still far from being able to quantify individual cancer risks. The frequency at which specific genes are mutated in a certain cancer cohort is an indicator of clinical importance. Even though frequent mutations (i.e. mutations that are more frequent than expected by chance in a specific cohort) are more likely to have tumor-related effects, individual cancer risks are most likely not fully explained by frequent mutations alone [1, 2]. Rare mutations could act in combination with frequent mutations or they may, entirely independent from frequent mutations, establish a significant risk for the patient on their own. Quantifying the risks associated with rare mutations has been complicated by the following reasons: (1) by definition, only a few patients carry these mutations, which reduces the probability of observing them in clinical studies, (2) even if they are observed, it is often difficult to quantify cancer risks statistically by comparing carriers with non-carriers due to insufficient statistical power, (3) complex interactions with other mutations (epistasis) may hide effects when analyzing single mutations in isolation, and (4) rare mutations of individual genes may have weak effects, but the co-occurrence of a sufficient number of such mutations in the same cell could significantly increase cancer risks. For example, a set of oncogenes with small individual effects but residing on the same chromosomal arm may establish a significant selective advantage if this chromosomal arm is amplified [3]. Essentially, we do not know how important rare mutations are in comparison to frequently observed mutations, simply because we are lacking the means to quantify their effects. The specific pattern of small mutations (single nucleotide variations or SNVs and small indels) in candidate genes can be used to prioritize putative driver genes without using epidemiological information [2–5]. Also, it has been shown that molecular networks can be used to better stratify patient populations by considering frequent and rare mutations together [1].

Apart from SNVs, DNA copy number alterations (CNAs) and chromosomal instability are a hallmark of cancer [6–8]. Further, CNA-affected genes with altered expression levels are more likely to be involved in tumorigenesis than affected genes with unchanged expression levels [9]. This has been exploited in previous studies to identify driver genes [10]. However, since CNAs frequently alter the expression levels of directly affected genes [9], these methods typically make many false positive predictions and require a large number of samples for a reliable prediction of potential key drivers. Other model-based approaches for the integrative analysis of gene copy number and gene expression data have been developed utilizing genetic linkage analysis [11] or network-based approaches [12–14] to identify major regulators driving tumorigenesis. All these methods (and many others) have greatly contributed to the identification of potential CNA tumor driver mutations and a better understanding of tumorigenesis, but none of these methods allows us to quantify the impact of rare gene CNAs.

Hence, novel computational methods are required to explore the long tail of rare mutations in cancer. An important step in this direction was done by [1], which enables the stratification of tumors that rarely share the same mutational profile into clinically relevant subtypes. Recently, another study proposed a network-based method that enables the identification of rare mutations involved in the perturbation of pathways and protein complexes involved in tumorigenesis [15]. This study predicted significantly mutated sub-networks containing dozens of genes rarely affected by mutations across different cancer types. Importantly, a common feature of [1] and [15] is the use of specifically designed network propagation algorithms to identify rarely mutated, but potentially relevant genes. However, we are still lacking methods for directly quantifying the impact of rarely affected genes on clinical endpoints such as survival.

Here, we present an approach exploiting the additional information contained in gene expression data to quantify potential effects of rare CNAs on clinically relevant endpoints. Our framework rests on the notion that regulatory relationships between genes are fairly robust across tumors, whereas the specific mutational pattern of a given tumor is virtually private [1, 16]. Put differently, most CNAs increase or decrease the activity of genes, while potentially only a small fraction of them alter the regulatory relationships between genes. Hence, by using large compendia of expression and mutation data sets, we can establish regulatory relationships between genes in cancer cells and quantify the effects of CNAs on gene expression. Such a model can subsequently be used to analyze individual tumors with known mutational patterns to quantify the impact of specific CNAs on global expression. Further, by relating those expression changes to clinical endpoints, we are able to quantify the effects of single CNAs on the survival of an individual patient. Using this framework, we can quantify direct (cis) effects and indirect (trans) effects of CNAs, we can identify key regulators in CNA regions (driver genes) with a particularly strong impact on the expression of clinically relevant genes, we can compare the importance of rarely mutated genes with frequently mutated genes, and we can quantify the combined effects of all CNAs on survival risk for an individual patient. Our analysis shows that usually many gene CNAs together influence individual patient survival by together impacting on common molecular pathways. At the individual level, it turns out that rare gene CNAs (less than 1 % frequency in a given cancer cohort) can be as important as frequent gene CNAs and we are able to specifically pinpoint potential candidate genes that are the most risky rare and frequent gene CNAs in individual patients.

## Results and discussion

### Cancer cell transcriptional network

**Fig. 1:**
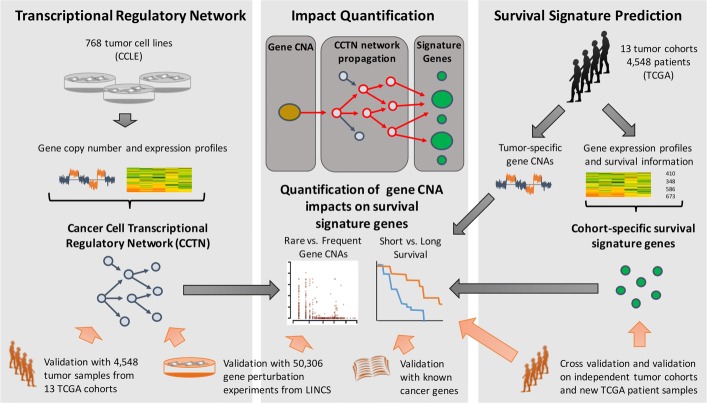
Methodological overview. *Left* A cancer cell transcriptional regulatory network (CCTN) was inferred from gene expression and corresponding gene copy number data of 768 cancer cell lines of the Cancer Cell Line Encyclopedia (CCLE) and validated using data of thousands of tumor patients from The Cancer Genome Atlas (TCGA) and thousands of gene-specific perturbation experiments from the Library of Integrated Network-based Cellular Signatures (LINCS). *Right* Signature genes whose expression correlated with patient survival were determined for individual TCGA cohorts and validated on independent test data. *Center* CCTN was applied to gene copy number profiles of individual tumor patients of TCGA cohorts to predict the impacts of individual gene CNAs on cohort-specific survival signature genes and to separate short- from long-lived patients. The impact prediction was validated using LINCS data, known cancer genes, and data from two independent clinical cohorts and new TCGA patients. *CCLE* Cancer Cell Line Encyclopedia, *CNA* copy number alteration, *CCTN* cancer cell transcriptional regulatory network, *LINCS* Library of Integrated Network-Based Cellular Signatures, *TCGA* The Cancer Genome Atlas

To predict the potential effects of gene CNAs in the specific environment of tumor cells, we computationally inferred a genome-wide transcriptional regulatory network from human cancer cell lines of 24 different tumor sites (Additional file 1: Figure S1) [17]. We termed this model the cancer cell transcriptional network (CCTN, Fig. 1). The input data for CCTN, consisting of genome-wide gene copy number and gene expression data, were strongly quality controlled for hybridization artifacts (e.g. Additional file 1: Figure S2): each microarray of the 991 cell lines was manually checked for potential artifacts and 768 cell lines were kept after this filtering step (Additional file 2: Table S1). To identify putative regulator genes for each of the considered 15,942 genes, we modeled the expression level of each gene (target gene) as a linear combination of the gene-specific copy number and the expression levels of all other potential regulator genes. Sparse regression based on lasso (least absolute shrinkage and selection operator) [18] was used to select those variables (target gene-specific copy number and expression levels of other regulator genes) that best predict the expression level of a specific target gene, while keeping the number of variables small. This approach has previously been shown to perform well in similar tasks [14, 19, 20]. We quantified the significance of the selected predictors of each target gene [21] and kept only edges with *p* values below 5×10^−5^ (unless stated otherwise). Further, we removed potentially spurious regulator genes in the chromosomal proximity of target genes that actually just reflect the copy number state of the target (see ‘Methods’ for details). This resulted in a sparse transcriptional regulatory network (CCTN) comprising 36,786 directed trans-acting edges between regulator and target genes (Additional file 1: Figure S3; Additional file 3: Table S2). We refer to all genes affecting the expression of at least one other gene in CCTN as regulator genes (i.e. genes with at least one outgoing edge in CCTN). Note that this regulator definition is driven by the network inference approach that selects the most relevant predictors of each response gene. Not every regulator gene is necessarily a direct transcriptional regulator of a corresponding response gene. Genes affected by at least one regulator gene are regarded as target genes (at least one incoming edge in CCTN; see ‘Methods’ for details).

**Fig. 2:**
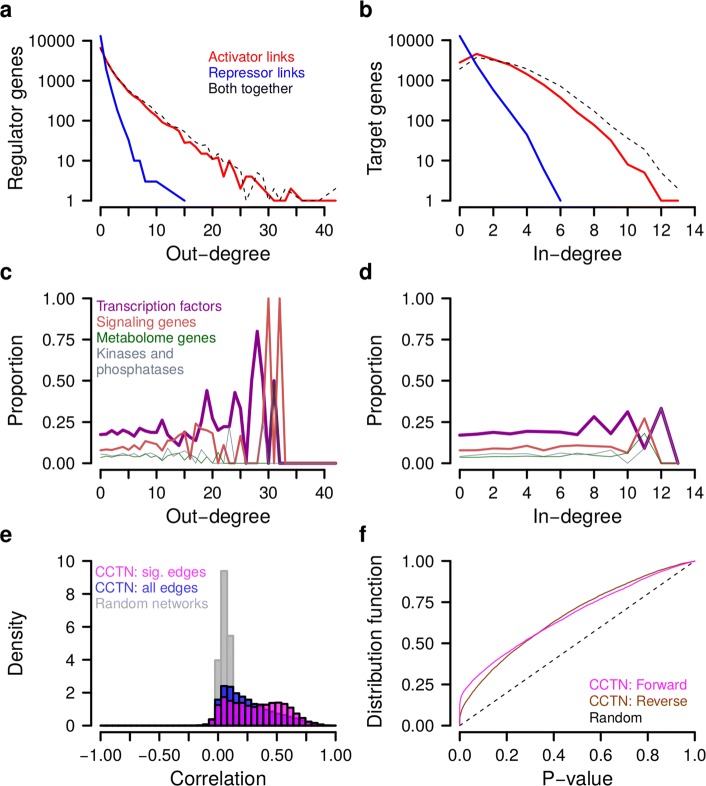
Cancer cell transcriptional network (CCTN) characteristics and validation. **a**, **b** Node degree distributions. **c**, **d** Functional annotation of network genes with respect to their node degrees. **a**, **c** Regulator genes. **b**, **d** Target genes. **e** Median gene-specific correlations between predicted and originally measured gene expression levels of individual genes in 13 TCGA cancer cohorts for CCTN including only significant edges (*pink*), CCTN using all edges (*blue*), and for random networks with the same complexity as CCTN with significant edges (*gray*). CCTN with significant edges predicts gene expression levels significantly better than CCTN with all edges (*p*<6×10^−169^) and random networks (*p*<2.2×10^−308^, Wilcoxon test). CCTN with significant edges was used for all subsequent analyses. **f** Cumulative *p* value distributions correlating experimentally measured and computationally predicted single-gene perturbations pooling results from all 13 TCGA cancer cohorts. *Forward*: *p* values of correlations between computed impacts flowing from a perturbed regulator to its targets and the corresponding experimentally measured impacts. The forward model specifies the basic CCTN properties that were used to make impact predictions (one-sided correlation test quantifying for each single-gene perturbation if the observed correlation between predicted and measured impacts is significantly greater than zero). *Reverse*: *p* values of correlations between computed impacts flowing in the reverse direction from the responding targets to their perturbed regulator and experimentally measured forward impacts. *Random*: Baseline for non-significant enrichment of small *p* values. See ‘Results and discussion’ and ‘Methods’ for details of the forward and backward models. The forward model predicted responses of single-gene perturbations significantly better than the reverse model (*p*<0.015 for each cohort) and than randomly expected (*p*<2.1×10^−23^ for each cohort, one-sided Kolmogorov–Smirnov test). *CCTN* cancer cell transcriptional regulatory network, *sig.* significant, *TCGA* The Cancer Genome Atlas

In total, 88 % of the genes (14,029 of 15,942) in CCTN were target genes, 60.6 % of the genes (9654 of 15,942) were selected as trans-acting regulators, and 27.3 % of the genes (4356 of 15,942) had a direct copy number effect that was always positively correlated with the underlying gene expression level (Additional file 3: Table S2). We further characterized the genes in CCTN based on their number of outgoing and incoming regulatory edges and found that the number of activator edges (32,521 of 36,786) is much greater than the number of repressor edges (4265 of 36,786) (Fig. 2 a and b). In addition, CCTN is characterized by a few central hub genes that have a large number of incoming and outgoing edges. Well-known cancer genes [2, 22] (e.g. TNFRSF17, FUS, IKZF1, GATA1, PAX8, SFPQ, IRF4, KLK2, COL1A1, MSL2, HSP90AB1, PHOX2B, CD79B, and LYL1) were significantly overrepresented among the 219 hub genes with more than 20 trans-acting regulatory edges to or from other genes (Fisher’s exact test: *p*<0.006; Additional file 4: Table S3). Further, regulator genes with a large number of outgoing edges (i.e. major regulators) were enriched for known transcription factors and signaling pathway genes (Fig. 2 c and d).

**Fig. 3:**
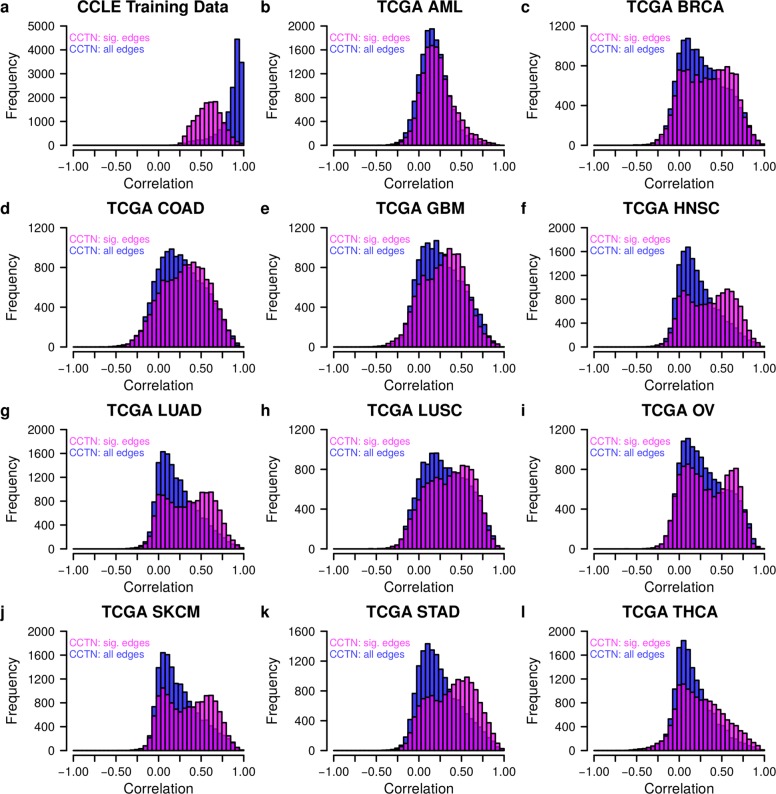
CCTN-based prediction of gene expression levels for cancer cell lines and tumor patients. Gene-specific correlations between predicted and originally measured gene expression levels of individual genes comparing CCTN including only significant edges (*pink*) to CCTN using all edges (*blue*). A greater proportion of positive correlations reflects a better predictive power. **a** Prediction quality for human cancer cell lines used to train CCTN. As expected, CCTN using all learned edges is better than CCTN with significant edges only. **b–l** Prediction quality of CCTN for tumor patients of 11 independent TCGA cohorts. CCTN including only significant edges reaches strongly improved predictions for the vast majority of cohorts in comparison to CCTN with all learned edges. See Additional file 1: Figure S5 for all cohorts. *AML* acute myeloid leukemia, *BRCA* breast invasive carcinoma, *CCLE* Cancer Cell Line Encyclopedia, *CCTN* cancer cell transcriptional regulatory network, *GBM* glioblastoma multiforme, *HNSC* head and neck squamous cell carcinoma, *LUAD* lung adenocarcinoma, *LUSC* lung squamous cell carcinoma, *OV* ovarian serous cystadenocarcinoma, *sig.* significant, *SKCM* skin cutaneous melanoma, *TCGA* The Cancer Genome Atlas, *COAD* Colon adenocarcinoma, *STAD* Stomach adenocarcinoma, *THCA* Thyroid carcinoma

CCTN was derived from cancer cell lines, i.e. in vitro data. To test the validity of CCTN for in vivo tumor cells, we used independent data of 13 different cancer cohorts from The Cancer Genome Atlas (TCGA) [23]. We downloaded gene expression and corresponding gene copy number data of 4548 tumor patients (Additional file 5: Table S4) and tested the predictive power of CCTN on each TCGA cohort separately by predicting the expression level of each gene for each tumor using its corresponding copy number and expression data. To quantify the quality of the prediction, we computed the correlation between the originally measured TCGA gene expression levels and the corresponding expression levels predicted by CCTN for each gene across all patients in a cohort. A strong positive correlation between originally measured and CCTN-predicted expression levels suggests that the respective gene is well predictable by CCTN. The vast majority of genes had a positive median correlation (median across the 13 TCGA cohorts) between the predicted and measured expression levels (Fig. 2 e): 94.7 % when using CCTN with all edges and 95.1 % when reducing CCTN to significant edges. Restricting CCTN to significant edges had an even more dramatic effect on the magnitude of the correlation between predicted and observed expression (Fig. 2 e; Wilcoxon–Mann–Whitney test: *p*<6×10^−169^, Fig. 3). An additional comparison of CCTN to random networks with the same complexity showed that CCTN makes significantly better predictions of expression levels for the vast majority of genes (Fig. 2 e; Wilcoxon–Mann–Whitney test: *p*<2.2×10^−308^). We further confirmed that both target gene-specific direct copy number effects and trans-acting regulator genes contributed to the correct prediction of expression levels (Additional file 1: Figure S4). Although the predictive power of CCTN was variable between individual genes and between tumor types, our model resulted in significant predictions for all considered patient cohorts (Fig. 3; Additional file 1: Figure S5) and was also very robust with respect to different *p* value cutoffs for including significant edges (Additional file 1: Figure S6).

We additionally compared CCTN, which was derived from in vitro cancer cell line data, to two network models derived from in vivo data of specific tumor types. These tumor type-specific network models tended to reach a slightly or moderately improved predictive power compared to CCTN on independent test data cohorts of the same tumor type (Additional file 1: Figure S7a and b). This is expected, because CCTN was trained on a mixture of cancer cell lines and is, therefore, not specific for a certain tumor type. However, CCTN reached nearly identical or slightly improved predictive power in comparison to non-tumor type-specific network models (Additional file 1: Figure S7c and d). This again suggests that CCTN can be generalized to different tumor entities.

In conclusion, CCTN works well on independent data and correctly captures the majority of potential regulatory relationships between genes in the in vivo tumor situation.

### Quantifying CNA impact on gene expression

Next we devised a method to quantify the impact of individual regulator genes on all other genes in the network (Fig. 1). This framework creates an impact matrix quantifying for each gene pair (*a,b*) the direct and indirect effect of gene *a* on the expression of gene *b* according to all existing directed regulatory network paths that link *a* to *b* in CCTN. The scoring also accounts for how well CCTN can predict the effects of mutations, i.e. CNA–target gene relationships that are poorly predicted get lower weights. Here, we operationally define the impact of a copy number change of gene *a* as its contribution to expression changes of gene *b*. That is, the impact is the (predicted) fraction of variance in the expression of a target gene caused by a specific gene CNA (see ‘Methods’ for details). The resulting impact matrix also accounts for the possibility of feedback cycles in CCTN, which could amplify (or dampen) the CNA effects.

We validated the correct prediction of impacts using individual gene perturbation data (LINCS L1000; see ‘Methods’ for details) [24, 25]. In these experiments, 933 genes (representatives of the human transcriptome) overlapped with CCTN genes and were perturbed on average 54 times and the expression responses of all other representative genes were measured, resulting in a total of 50,306 perturbation experiments (Additional file 6: Table S5). Note that the perturbations were repressing (knock-down) and increasing (overexpression) the transcript levels, which functionally mimics the effects of CNAs. We determined the significance of positive correlations between predicted and observed impacts across all 13 TCGA cancer types (see ‘Methods’) and found a strong enrichment of small *p* values (Fig. 2 f; Additional file 1: Figure S8), confirming that the impact score is predictive for direct and indirect effects (one-sided Kolmogorov–Smirnov test comparing the *p* value distribution under the forward model to a uniform distribution expected under a random model: *p* values across TCGA cohorts ranging from 1.2×10^−45^ for thyroid carcinoma to 2.1×10^−23^ for skin cutaneous melanoma). The perturbation-expression data also allowed us to validate the direction of predicted effects: the correlated expression of two genes does not reveal which of the two genes is affecting which or if they are together under the control of a third gene. Since for perturbation experiments the direction of effects is known, we can use these data to assess the correct prediction of directional effects. We, therefore, compared the quality of CCTN predictions using a forward model (i.e. a model with correctly pointing interactions) with those of a reverse model (i.e. a model with inverted interactions). If the directionality information in CCTN is not meaningful, we would expect both models to perform equally well on the LINCS L1000 data. However, we observed that the forward model performed significantly better than the reverse model (Fig. 2 f; Additional file 1: Figure S8: forward model contains more small *p* values than the reverse model: *p* values across TCGA cohorts ranging from 0.0004 for stomach adenocarcinoma to 0.015 for skin cutaneous carcinoma), suggesting that CCTN mostly correctly predicts the direction of effects.

### Identification of tumor type-specific survival signatures

The CCTN-derived impact matrix has the ability to predict how a CNA of a gene affects the expression of all other genes in the network. To quantify the clinical relevance of individual gene CNAs, we determined genes whose expression levels are predictive of patient survival (Fig. 1). We developed an approach based on a random forest (RF) [26] to determine genes whose expression levels were significantly correlated with patient survival in individual TCGA cohorts (see ‘Methods’ for details). We chose RF for this task, because RF is particularly robust against overfitting, can handle complex non-additive relationships between predictor variables, and is able to exploit the molecular heterogeneity within a tumor cohort, which is essential for robust survival prediction and the characterization of survival-associated genes. In addition, an in-depth model comparison has previously shown that RF is among the best methods for the prediction of patient survival from gene expression data [27].

We initially tested our RF approach on all cohorts with more than 20 patients with survival information (8 of 13 TCGA cohorts; Additional file 5: Table S4). Testing of the resulting models on held-out patient samples (cross-validation) revealed that at least 100 patients with survival information were required to reach modest or more significant survival predictions (Additional file 1: Figure S9), which is in good accordance with previous findings for selected TCGA cohorts [28]. Correlations between RF-predicted and real patient survival on held-out samples were in the range of 0.12 to 0.35 for six TCGA cohorts (Additional file 1: Figure S9) with corresponding modest significance (*p*<0.1) for acute myeloid leukemia (AML) and skin cutaneous melanoma (SKCM), and stronger significance (*p*<0.013) for head and neck squamous cell carcinoma (HNSC), glioblastoma multiforme (GBM), lung adenocarcinoma (LUAD), and ovarian serous cystadenocarcinoma (OV). The RF approach was not predictive for breast invasive carcinoma (BRCA) and lung squamous cell carcinoma (LUSC) (Additional file 1: Figure S9), possibly due to limited numbers of tumor samples or inadequate follow-up time. In addition, we also compared our RF approach to random survival forest (RSF) [29]. RSF can handle right-censored data to gain additional information from patients that were alive. However, our RF approach consistently reached better predictions of patient survival on held-out patient samples than RSF with and without censoring except for slightly improved survival predictions for AML (Additional file 1: Figure S10). RSF was also not predictive for BRCA and LUSC (Additional file 1: Figure S10). We further validated the RF-based survival prediction on GBM data from an independent patient cohort that was not part of the TCGA initiative [30]. The prediction of survival was highly significant, indicating that our RF model can make robust, potentially clinically relevant predictions (Additional file 1: Figure S11, *r*=0.52, *p*<0.0006, 36 patients). Thus, we focused on our RF approach and only kept the six cohorts (AML, GBM, HNSC, LUAD, OV, and SKCM) in all subsequent analyses, but note that the performance on held-out patients indicates a potentially greater clinical utility for HNSC, GBM, LUAD, and OV than for AML and SKCM.

Next, we ranked all genes based on their importance for predicting patient survival and filtered for the most important genes (signature genes) in each cohort by considering gene-specific contributions to the average correlation between RF-predicted and real patient survival (see ‘Methods’ and Additional file 1: Figure S12). The number of selected signature genes for the six cohorts ranged from eight for AML to 199 for GBM for a correlation cutoff of greater than 0.1 (Additional file 7: Table S6; Additional file 1: Figure S12). As expected, a complex clinical endpoint such as survival cannot be predicted from a small number of genes. Accordingly, the correlation of individual gene expression levels with survival was weak, thus underlining the need to consider multiple marker genes in combination to obtain significant predictions of patient survival (Additional file 1: Figure S13, *p*<0.004 for all cohorts except for a more modest significance for genes positively correlated with HNSC survival reaching *p* < 0.043).

We further analyzed the obtained survival signatures for known cancer genes [22]. We found, for example, that the tumor suppressor NF1, a marker for mesenchymal GBMs [31], and the oncogene DNMT3A, a DNA methyltransferase impacting on proliferation and cell survival under hypoxic conditions [32], were part of the GBM survival signature. Interestingly, the transcription factor HOXD13 [33], which has been recently associated with poor survival of breast cancer patients [34], was part of the LUAD survival signature. Further, the tumor suppressor CAMTA1, a transcription factor involved in the regulation of cell growth and differentiation of neuroblastomas [35], and the tumor suppressor NIPBL, a cohesin regulator involved in developmental regulation, growth delay, and DNA repair [36], were part of the OV survival signature. We finally note that signature genes are not necessarily directly affected by CNAs. They should rather be considered as targets of driver mutations.

### Impact of individual gene CNAs on survival signature genes

Using the CCTN-derived impact measures as defined above, we next quantified the contribution of each gene’s copy number state on the expression of survival signature genes in each individual tumor (Fig. 1). First, we performed an integrated validation of the entire impact computation pipeline observing a significant positive correlation between patient-specific cumulative impacts of all individual gene CNAs and patient survival using an independent GBM cohort [30] that was not used for learning of any of our models for network effect quantification and survival signature prediction (Additional file 1: Figure S14a, Spearman rank correlation test: rho =0.33, *p*=0.024, 36 patients, see ‘Methods’ for details). This significant correlation between our impact scores and survival was not necessarily expected, as it does not account for mutations other than CNAs. In addition, these patient-specific impact scores further enabled a significant classification into short and long survival groups using Kaplan–Meier curves (Additional file 1: Figure S14b, *p*<0.02).

After validating our impact scoring, we focused on the TCGA cohorts. For each mutated gene, we averaged its corresponding impact scores across all signature genes, yielding a single impact score that quantifies the contribution of this specific gene CNA on the expression of all survival signature genes. We selected high-impact gene CNAs for each of the six TCGA cohorts and corrected for multiple testing by comparing the originally obtained gene CNA-specific impact scores against corresponding gene-specific impact scores obtained under ten random networks of the same complexity as CCTN (Additional file 1: Figure S15, *q*<0.006 for all cohort-specific selected genes, see ‘Methods’ for details). We further confirmed that these genes were enriched for known cancer genes [22] (Fisher’s exact tests: *p*<0.03 except for AML and SKCM).

**Fig. 4:**
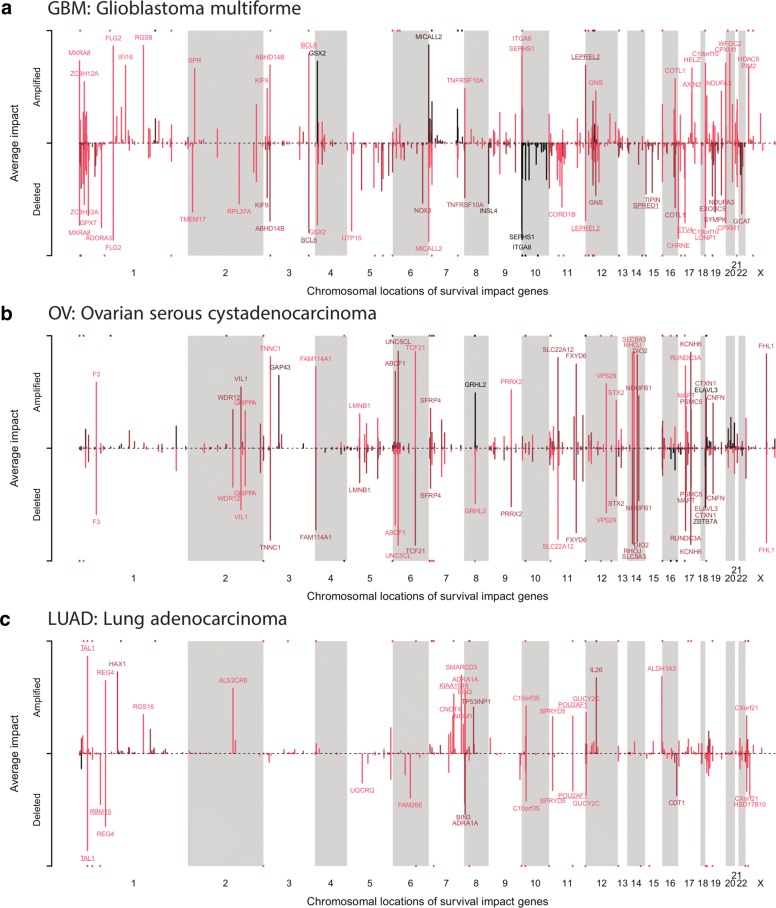
Chromosomal locations of gene CNAs with high impact on survival signatures. **a** Glioblastoma multiforme (GBM). **b** Ovarian serous cystadenocarcinoma (OV). **c** Lung adenocarcinoma (LUAD). Genes are colored based on their observed mutation frequency (*light red*: rare, *black*: frequent). Heights of the peaks reflect the impact on survival signature genes. Selected gene names are displayed depending on their impact strength. Gene names of known cancer genes are underlined. *Colored dots* above or below genes additionally indicate the positions of previously reported cancer genes that were predicted as high-impact genes by CCTN. Only genes with significant impacts on survival signatures are shown (*q*<0.006 for all cohort-specific genes, one-sided Wilcoxon test comparing the gene-specific impact score obtained under CCTN against corresponding impact scores obtained under random networks of the same complexity as CCTN, see ‘Methods’ and Additional file 1: Figure S15 for details). The cohort-specific genes shown are further significantly enriched for known cancer genes (*p*<0.03 for each cohort, Fisher’s exact test). *GBM* glioblastoma multiforme, *LUAD* lung adenocarcinoma, *OV* ovarian serous cystadenocarcinoma

In addition, our impact scoring identified many genes with established roles in the respective tumor classes (Fig. 4). For example, TAL1 had the greatest impact score among all LUAD-associated genes and had previously been identified as a hub transcriptional regulator in LUAD with effects on TGF-beta signaling [37]. Another example is TNNC1, which is involved in the metastatic potential of ovarian cancer cells [38] and was among the top-ranking OV impact genes. Further, histone deacetylases (HDAC) have a well-established role in tumorigenesis and serve as important cancer drug targets. We correctly detected HDAC6 as a high-impact gene in GBM [39, 40]. The predicted high-impact gene CNAs impacting on AML, HNSC, and SKCM survival signatures are shown in Additional file 1: Figure S16. Apart from just confirming well-known tumor markers, our CCTN approach also provides supporting evidence for previously reported candidate genes and has the potential to reveal novel candidate genes impacting on survival. Both are demonstrated by the following examples.

#### Different gene CNAs putatively impact on the same survival signature gene

For example, HAX1 has been suggested to be involved in lung cancer [41]. We confirm that an increased HAX1 copy number contributes to an increased HAX1 expression level with downstream effects on the expression of TSEN15 (Additional file 3: Table S2). TSEN15, a LUAD survival signature gene, is involved in the tRNA splicing required for cell growth and division [42]. Our impact analysis further predicts TSEN15 as a downstream target of two other high-impact gene deletions of PLXNB2 and CHAC1 that both strongly impact on the expression of TSEN15. PLXNB2 is involved in cell proliferation and migration [43]. CHAC1 is a negative regulator of Notch signaling [44], involved in apoptosis [45] and known to function in other cancers [46, 47]. Thus, these three genes impact on a common molecular endpoint that is correlated with patient survival.

#### Duplication of chromosome 7 in GBM suggests further driver genes in addition to EGFR

It has previously been suggested that the clustering of driver genes on chromosomal arms may explain frequent amplifications or deletions of large chromosomal regions [3]. Our results support this notion and assist in the understanding of specific large deletions and amplifications. For example, the duplication of chromosome 7 is one of the most prominent chromosomal mutations found in GBMs [48] (Fig. 4 a). Despite the frequency of this event, we have only an incomplete understanding about the genes in this region driving the cancerous phenotype. The amplification of the oncogene EGFR [49] on chromosome 7 is involved in GBM etiology. However, most likely additional genes on chromosome 7 contribute to GBM development and prognosis [50]. This is supported by our finding that patient-specific cumulative impact scores of all genes on chromosome 7 explain survival significantly better than the EGFR impact score alone (Meng’s *t*-test on independent GBM cohort [30]: *p*<0.005). We identified additional candidate genes on chromosome 7 with a high impact on GBM survival signature genes (Additional file 8: Table S7), including genes involved in (1) cell adhesion and migration, cytoskeletal organization, and neurite outgrowth (ARHGEF5, BAIAP2L1, MICALL2, SEMA3C, and TNS3), (2) transcriptional regulators and chromatin remodelers (ACTL6B, EZH2, H2AFV, IKZF1, and MLL3), and (3) cell proliferation, apoptosis, and DNA damage response (GIMAP6, HBP1, MCM7, PAXIP1, PPIA, SAMD9, and TBRG4). That EZH2 and MLL3 were found to be affected by small somatic mutations further supports their potential role in GBM etiology [48, 51].

#### Amplifications of tumor suppressors can contribute to longer survival

Interestingly, we also observed several high-impact genes that were amplified in some patients and deleted in others. The effect of an amplification or deletion may be conditional on other concurrent mutations, which is one possible explanation for this observation. However, we also detected some instances of positive gene CNAs where the respective CNA was associated with increased survival. For example, amplifications of the tumor suppressor genes WAC in GBM (97 tumors with amplifications vs 218 tumors with normal gene copy number, Fig. 4 a, chromosome 10, p-arm) and CDH1 in OV (61 tumors with amplifications vs 174 tumors with normal gene copy number, Fig. 4 b, chromosome 16, q-arm) were associated with significantly prolonged survival (*t*-test *p* values 0.0005 and 0.009, respectively).

#### Rare patient-specific gene CNAs strongly impact on survival signatures

**Fig. 5:**
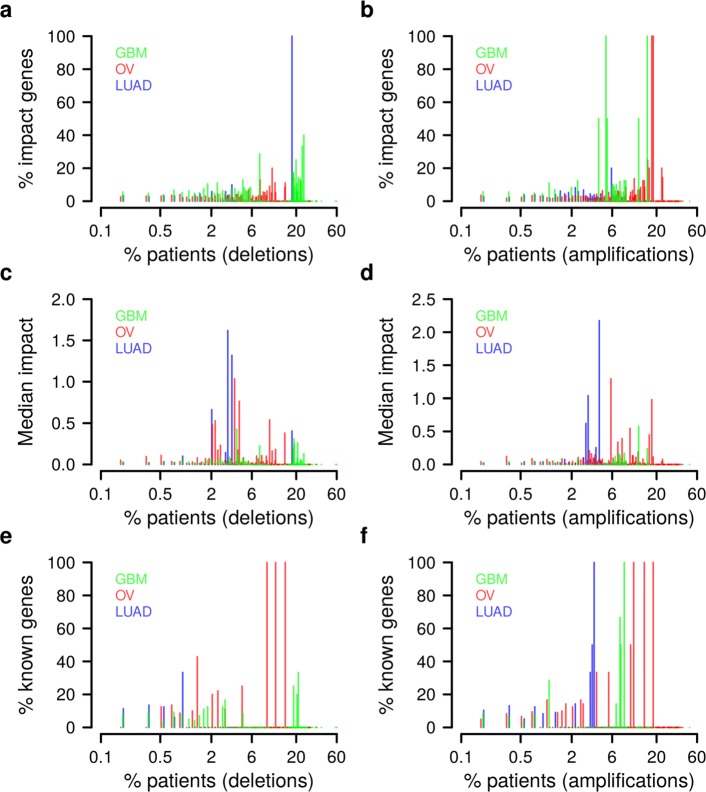
Cohort-specific characterization of gene CNAs with a high impact on survival signature. All gene deletions and amplifications in each TCGA cohort (GBM, OV, and LUAD) with a high impact on the corresponding survival signature were grouped based on their mutation frequency shown in log-scale along the *x*-axes. **a**, **b** Percentage of genes in each bin belonging to the cohort-specific genes with a high impact on survival signature genes. More frequently mutated genes are more likely high-impact genes (*p*<0.03 for each cohort, one-sided correlation test). **c**, **d** Median impact of high-impact genes in each frequency bin. The median impact quantifies the contribution of all high-impact gene CNAs in a bin to the variation of the expression levels of all cohort-specific survival signature genes. Average percentages of explained variance of survival signature expression computed for all high-impact gene CNAs were used to determine the median impact per bin. **e**, **f** Proportion of known cancer genes among the high-impact genes in each bin. Known tumor suppressor and oncogenes are enriched among more frequently mutated genes with high survival impact (*p*<0.005 except for LUAD deletions, one-sided correlation test). *CNA* copy number alteration, *GBM* glioblastoma multiforme, *LUAD* lung adenocarcinoma, *OV* ovarian serous cystadenocarcinoma

**Fig. 6:**
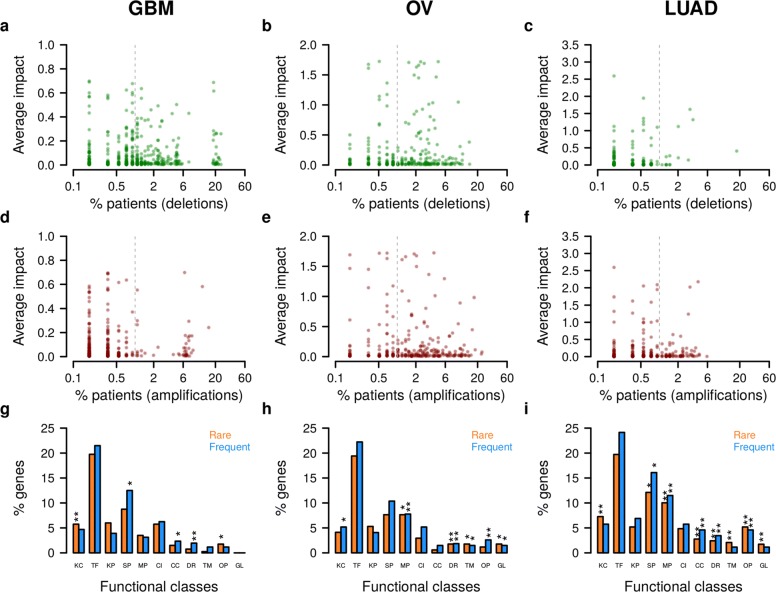
Individual impact of rare and frequent gene CNAs with high impact on survival signatures. Characterization of TCGA cohorts GBM, OV, and LUAD. **a–f** Average impact of gene deletions (**a–c**) and amplifications (**d–f**) on the expression of cohort-specific survival signature genes. The average impact value of each specific gene CNA quantifies the average contribution of this gene CNA to the variation of the expression levels of all cohort-specific survival signature genes in percentage of explained variance. The *vertical gray dashed line* at 1 % of patients defines the cutoff used to separate rare from frequent CNAs. The *x*-axes are in logarithmic scale. **g–i** Corresponding joint functional annotation of deleted and amplified impact genes. Genes were classified as rarely (CNA frequency <1 %) or frequently (CNA frequency ≥1 %) mutated. The proportion of genes in selected functional classes are shown. Significant enrichment of an individual category is shown above bars as * (*p*<0.05) and ** (*p*<0.01, Fisher’s exact test). Note that the height of the bars doses not necessarily correlate with significance due to the different sizes of the functional classes. *CC* cell cycle, *CI* cell–cell interaction, *CNA* copy number alteration, *DR* DNA replication, *GBM* glioblastoma multiforme, *GL* glycolysis, *KC* known cancer genes, *KP* kinases and phosphatases, *LUAD* lung adenocarcinoma, *MP* metabolic pathways, *OP* oxidative phosphorylation, *OV* ovarian serous cystadenocarcinoma, *SP* signaling pathways, *TCGA* The Cancer Genome Atlas, *TF* transcription factors, *TM* telomere maintenance

After having established confidence in the impact scoring, we next determined the number of genes that have to be considered in combination to explain a certain fraction of survival risk in a given patient (Additional file 1: Figure S17). We revealed considerable variation between patients with respect to how many and to what extent gene CNAs affect survival signature genes. Up to 100 gene CNAs contributed together to the individually explained risk. Next, we focused on the relationship between impact and frequency at which gene CNAs occur in a tumor cohort (Fig. 5 for GBM, OV, and LUAD; Additional file 1: Figure S18 for AML, HNSC, and SKCM). As expected, more frequently mutated genes are more likely high-impact genes (Fig. 5 a and b, correlation tests: *p*<0.03) and accordingly, the median impact of frequently mutated genes tends to be higher than that of rarely mutated genes (Fig. 5 c and d). Also, known tumor suppressor and oncogenes are enriched among more frequently mutated genes with high impact (Fig. 5 e and f, correlation tests: *p*<0.005 except LUAD deletions). However, even though frequently mutated genes had on average larger impacts on signature genes, a substantial number of rarely mutated genes (frequency <1 %) also had strong impacts (Fig. 6 for GBM, OV, and LUAD; Additional file 1: Figure S19 for AML, HNSC, and SKCM). Importantly, some of these genes with CNAs in only one, two, or three individuals per cohort had impacts that were larger than those of many frequently mutated genes (Fig. 6 a–f; Additional file 1: Figure S19a–f; Additional file 8: Table S7). In addition, a significant fraction of the low-frequency high-impact genes in GBM, OV, and LUAD have previously been reported as cancer genes [22] in other tissues (Fisher’s exact tests: *p*<0.009). In conclusion, the patient-specific expression pattern of survival signature genes can substantially be driven by individual rare gene CNAs, which is consistent with recent findings that patient-specific mutation patterns impact on survival [1].

#### Number of gene CNAs alone or single-gene tests do not allow to quantify survival impacts

The previous examples have shown that CCTN allows us to pinpoint rare and frequent gene CNAs that act on patient survival. We further analyzed if similar results can also be obtained using two alternative approaches. First, we considered the gene CNA burden of each patient, but we did not find any significant correlation between the number of CNA-affected genes (rare, frequent, or both together) and survival in any of the six TCGA cohorts (see Additional file 1: Text S2 for details). Second, we considered single-gene tests to determine if patients with a specific gene CNA had significant differences in survival compared to patients without this gene CNA. Considering the six TCGA cohorts, we were able to detect only some gene CNAs for AML that were significantly associated with survival, but as expected there were no rare gene CNAs among those genes (Additional file 1: Figure S20; see Additional file 1: Text S3 for details). Thus, our CCTN-based impact scoring approach allows us to gain novel insights into the putative impacts of specific gene CNAs.

### Chromosomal location instead of gene function explains CNA frequency

**Fig. 7:**
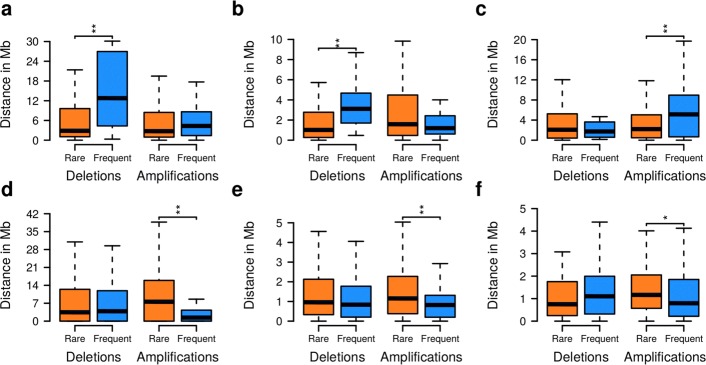
Distances of rare and frequent high-survival-impact gene CNAs from genomic features. Selected examples for LUAD, GBM, and OV considering chromosomal distances of all gene CNAs with a high impact on cohort-specific survival signature genes from genomic features. See Additional file 1: Figure S21 for distance distributions of all tumor cohorts. **a** Distances of rare and frequent LUAD gene CNAs from known oncogenes. **b** Distances of rare and frequent LUAD gene CNAs from known essential genes. **c** Distances of rare and frequent LUAD gene CNAs from known tumor suppressor genes. **d** Distances of rare and frequent GBM gene CNAs from known fragile sites. **e** Distances of rare and frequent GBM gene CNAs from known frequently occurring germ-line CNVs. **f** Same as (**e**), but for OV. Significant differences in distances of rare and frequent CNAs from genomic features are represented by * (*p*<0.05) and ** (*p*<0.01, Wilcoxon test). *CNA* copy number alteration, *GBM* glioblastoma multiforme, *LUAD* lung adenocarcinoma, *OV* ovarian serous cystadenocarcinoma

Genes with very similar survival impact scores can have very distinct CNA frequencies in the same tumor class (Fig. 6 a–f). We sought to identify factors explaining why some of those gene CNAs are observed much more rarely than others. A first hypothesis was that rare high-impact mutations occur later in the tumor etiology and affect different endpoints than frequent gene CNAs. For example, frequent mutations might primarily drive the neoplastic transformation and thus affect proliferation, DNA damage response, and apoptosis, whereas rare CNAs might affect angiogenesis, metastatic potential, or drug resistance. However, functional classification of rare and frequent gene CNAs did not yield striking differences between the two CNA groups (Fig. 6 g–i). Instead of function, the chromosomal location of genes seems to explain variable gene CNA frequencies better. The close placement of two tumor-relevant genes with antagonistic effects reduces the frequency of observing the respective CNAs [3]. For example, an oncogene and a tumor suppressor gene located in close chromosomal proximity reduce the chance that a CNA in that region will be beneficial for the tumor. We observed similar effects that distinguished rare from frequent gene CNAs in our data (Fig. 7; Additional file 1: Figure S19). For example in LUAD, frequent gene deletions are on average significantly further away from oncogenes [2, 3] and essential genes [52] than rare gene deletions (Fig. 7 a and b, one-sided Wilcoxon tests: *p*<0.003, average distance from oncogenes: 14.5 vs 6.3 Mbp, average distance from essential genes: 3.8 vs 2.9 Mbp), while gene amplifications are typically significantly further away from tumor suppressor genes [2, 3] (Fig. 7 c, one-sided Wilcoxon test: *p*<0.002, average distance from tumor suppressor genes: 7.2 vs 3.7 Mbp). Our data further show that the distance to fragile genomic sites [53] is correlated with the observed frequency of gene CNAs impacting on survival signatures. For example, in GBM, frequently amplified genes are significantly closer to fragile sites than rarely amplified genes (Fig. 7 d, one-sided Wilcoxon test: *p*<5×10^−5^, average distance from fragile sites: 4.7 vs 10.7 Mbp). Finally, the distance to frequently observed germ-line copy number variations (CNVs) [54] is correlated with the observed frequency of high-impact gene CNAs acting on survival signatures. For example, frequently amplified genes in GBM and OV are significantly closer to known germ-line CNV sites than rarely amplified genes (Fig. 7 e and f, one-sided Wilcoxon tests: *p*<0.016, average distance from tumor germ-line CNV sites for GBM is 0.98 vs 1.7 Mbp and 1.4 vs 1.7 Mbp for OV). Interestingly, these correlations between CNA frequency and genomic positioning were independent of survival impact, but highly specific for tumor type (Fig. 4; Additional file 1: Figure S21), suggesting that the molecular mechanisms leading to and maintaining CNAs are tissue-specific. Taken together, these analyses support that the chromosomal location of a gene rather than its function determines variable CNA frequencies among genes with similar impact.

### Indirectly acting tumor-specific gene CNAs clearly improve survival prediction

**Fig. 8:**
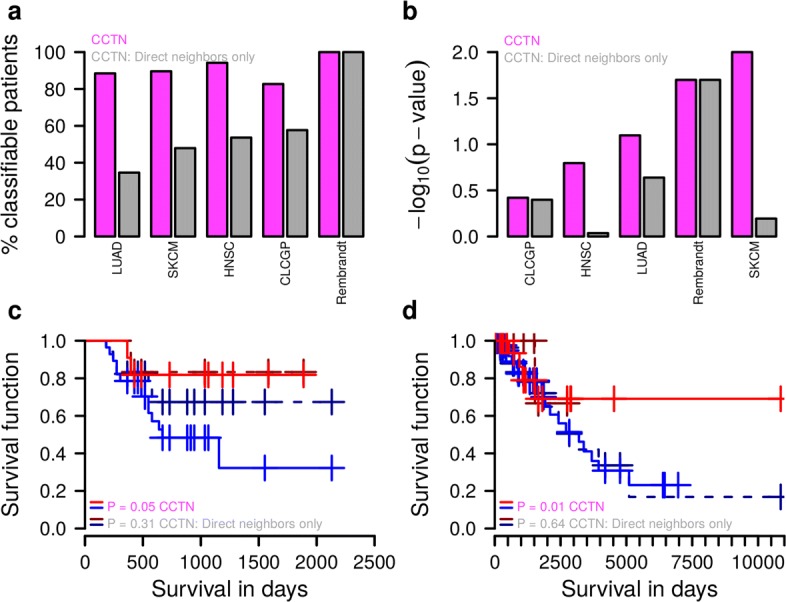
Model comparison highlighting the importance of indirectly acting gene CNAs for survival analysis. Comparison of our standard survival impact quantification (CCTN) to a basic version (CCTN: direct neighbors only). The standard approach considers all gene CNAs that directly or indirectly impact on survival signature genes, whereas the basic version accounts only for directly acting gene CNAs to realize a classification into short- and long-lived patients. **a** Percentage of patients per cohort that can be classified by both approaches. CCTN reaches significantly increased numbers of classifiable patients compared to the basic version (*p*<2.2×10^−16^, Fisher’s exact test). **b** Separation quality of patients classified as short- or long-lived by both approaches. Corresponding *p* values quantify the separation between patients classified as short- or long-lived with respect to differences of the short- and long-lived Kaplan–Meier curve obtained for each approach under consideration of all tumor-specific gene CNAs. The *y*-axis is plotted in negative logarithmic scale. CCTN reaches a clearly improved patient separation for the majority of cohorts in comparison to the basic version. **c** Kaplan–Meier curves for separation of CLCGP patients into short and long survival based on frequent gene CNAs. CCTN reaches a significantly improved patient separation. **d** Like (**c**), but here for SKCM patients considering all patient-specific gene CNAs. *CNA* copy number alteration, *CCTN* cancer cell transcriptional regulatory network, *HNSC* head and neck squamous cell carcinoma, *LUAD* lung adenocarcinoma, *SKCM* skin cutaneous melanoma

Our CCTN-based impact quantification approach utilizes all patient-specific gene CNAs that directly or indirectly act on patient survival to distinguish between short- and long-lived patients (Additional file 1: Figure S14). We further analyzed the value of integrating indirectly acting gene CNAs by comparing our approach to a basic version that only considers CNAs of genes in the direct network neighborhood of survival signature genes. Both impact scoring approaches utilize CCTN as the basis for enabling a fair comparison (see Additional file 1: Text S4 for details). To compare both approaches, we considered five independent test cohorts [Rembrandt: GBM [30]; Clinical Lung Cancer Genome Project (CLCGP): LUAD [55]; newly added TCGA patients: LUAD, SKCM, and HNSC; Additional file 5: Table S4]. First, we determined the numbers of patients that could be assigned to the short or long survival group based on their individual gene CNAs. We found that the integration of indirectly acting gene CNAs led to significantly increased numbers of classifiable patients for four out of five cohorts (Fig. 8 a, *p*<2.2×10^−16^, Fisher’s exact test). This is explained by the observation that many patients did not have gene CNAs in the direct network neighborhood of survival signature genes, which prohibits a classification by the basic version. Second, we compared the separation quality between patients classified as short- and long-lived by both impact scoring approaches. In two out of five cohorts (Rembrandt and CLCGP), we did not find a significant difference in the separation between short- and long-lived patients (Fig. 8 b). For all other cohorts (LUAD, SKCM, and HNSC), including indirect effects significantly improved the survival prediction compared to considering only direct effects (Fig. 8 b). For example, this significant performance improvement is also observed when comparing the survival curves of short- and long-lived CLCGP and SKCM patients utilizing only frequent or all gene CNAs (Fig. 8 c and d). Thus, the integration of indirectly acting gene CNAs into the prediction of short or long patient survival is an important factor to improve the classification of patients.

### Frequent and rare tumor-specific gene CNAs contribute to survival prediction

**Fig. 9:**
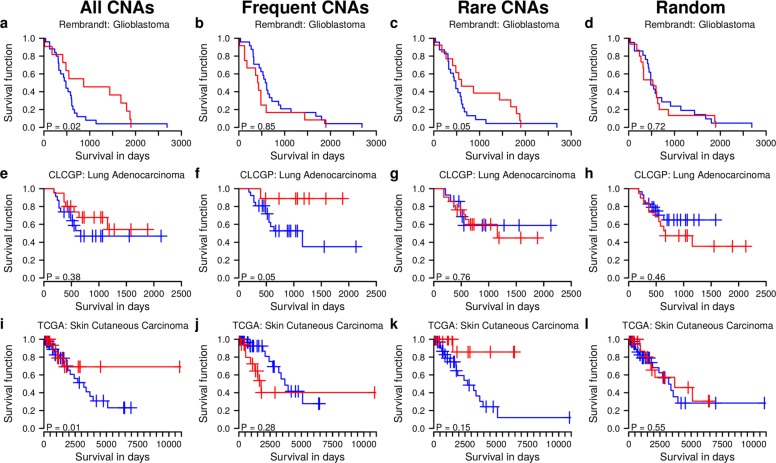
Impact of rare and frequent patient-specific gene CNAs on survival. Kaplan–Meier curves for patients with negative (*blue*) and positive (*red*) average impact of their corresponding tumor-specific gene CNAs on cancer type-specific survival signature genes. Shown are results for independent tumor cohorts (Rembrandt: GBM; CLCGP: LUAD; TCGA: SKCM new patients) that were not involved in any step of CCTN network inference or RF-based prediction of survival signature genes. CCTN derived from cancer cell lines and cancer type-specific survival signature genes identified from TCGA cohorts were used to investigate the impact of rare and frequent gene CNAs on patient survival for these cohorts. Separation of long- and short-lived patients by CCTN is expected to be predictive if patients with positive average survival impact scores (*red*) tend to survive longer than patients with negative impact scores (*blue*). The corresponding *p* value quantifies if the *red curve* is significantly above the *blue curve* in comparison to random class label permutations. **a–d** GBM results. **a** Patients with positive average impact scores on survival signature genes (*red*) tend to survive significantly longer than patients with negative impact scores (*blue*). **b** Frequent patient-specific gene CNAs (frequency ≥1 % in corresponding training cohort) alone cannot explain the significant difference in (**a**). **c** Rare patient-specific gene CNAs (frequency <1 %) significantly contribute to the observed significant differences in (**a**). **d** Loss of patient separation into short and long survival for patient-specific survival impact scores computed based on all patient-specific gene CNAs under a random network. **e–h** LUAD results. Similar to GBM, but in contrast to GBM, frequent mutations alone contribute to a significant separation between long- and short-lived patients. **i–l** SKCM results. Overall trends are comparable with those observed for GBM. *CLCGP* Clinical Lung Cancer Genome Project, *CCTN* cancer cell transcriptional regulatory network, *CNA* copy number alteration, *GBM* glioblastoma multiforme, *LUAD* lung adenocarcinoma, *RF* random forest, *SKCM* skin cutaneous melanoma, *TCGA* The Cancer Genome Atlas

We have already shown that individual frequent and rare tumor type-specific gene CNAs can have strong impacts on survival signature genes (Fig. 4). This motivated us to analyze further if tumor-specific gene CNAs of individual patients can be used to distinguish between short and long survival. A slight modification of our impact quantification algorithm enabled us to compute personalized impacts for each gene CNA in a patient-specific tumor (see ‘Methods’ and Additional file 1: Text S1 for details). This personalized impact score quantifies if the corresponding gene CNA has an inhibitory impact (negative impact value) or an activating impact (positive impact value) on a tumor type-specific survival signature gene. To account for the direction of the survival association of each signature gene, we multiplied this regulatory impact with the corresponding sign of the correlation observed between the expression levels of the signature gene and the survival of patients. This resulted in a personalized score that quantifies the impact of each tumor-specific gene CNA on survival. The score captures, for example, that a gene CNA with an inhibitory impact on a signature gene that is negatively correlated with survival has a potential positive impact on survival (may increase survival), whereas a gene CNA with an inhibitory impact on a positively correlated survival signature gene has a potential negative impact on survival (may decrease survival). To get an integrated survival score for all gene CNAs of a patient-specific tumor, we summarized the tumor-specific gene CNA scores to an average patient-specific survival impact score. Based on the score derivation, negative scores are expected to be associated with shorter patient survival than positive scores. We used these patient-specific average survival impact scores to analyze if the CCTN-based impact quantification allows us to distinguish between short and long survival coupled with a systematic analysis to quantify how frequent and rare gene CNAs contribute to the discrimination. In total, we utilized data of 292 tumor patients from five independent tumor cohorts including GBM patients from Rembrandt [30], LUAD patients from CLCGP [55] and newly added TCGA patients from LUAD, SKCM, and HNSC (Additional file 5: Table S4; no new GBM and AML patients were added to TCGA and only too few new OV patients were available from TCGA) that were not involved in any step of the CCTN inference nor in any step of the survival signature gene prediction before. To analyze these new patient samples, we used CCTN as derived from the cancer cell lines in combination with the corresponding tumor type-specific survival signature genes derived for the TCGA cohorts representing the same tumor entity to perform patient-specific impact quantification. An analysis of the contributions of (1) all patient-specific gene CNAs, (2) only patient-specific frequent gene CNAs, and (3) only rare patient-specific gene CNAs to the separation of long- and short-lived patients is shown in Fig. 9 for selected cohorts (Rembrandt: GBM; CLCGP: LUAD and SKCM; new TCGA patients). Results obtained for the other cohorts are shown in Additional file 1: Figure S22 (new LUAD and HNSC patients from TCGA). Importantly, this patient stratification was better than using random networks of the same complexity as CCTN, which led to a collapse of the impact quantification system (Fig. 9; Additional file 1: Figure S23), implying that our scoring is able to prioritize successfully gene CNAs with strong impacts on individual patient survival.

In more detail, for Rembrandt GBMs and new SKCM patients from TCGA, we observed a significant stratification into long- and short-lived patients (Fig. 9 a and i, *p*<0.02). Interestingly, rare gene CNAs (frequency <1 % in the training cohort) strongly contributed to the correct impact scoring for GBM and SKCM (Fig. 9 c and k). In contrast to GBM and SKCM, the full scoring based on all patient-specific gene CNAs was not predictive for LUAD samples from CLCGP (Fig. 9 e), whereas a scoring based only on frequent gene CNAs (frequency ≥1 % in the training cohort) was predictive for long and short survival (Fig. 9 f, *p*<0.05). Rare gene CNAs did not improve the LUAD patient stratification (Fig. 9 g). These trends were also confirmed by an independent analysis of new LUAD patients from TCGA (Additional file 1: Figure S22a–c, *p*<0.01 for frequent gene CNAs). Finally, we note that our impact scoring was not predictive for HNSC patients (Additional file 1: Figure S22i–l, Figure S23q–t), possibly due to the great molecular heterogeneity of HNSC tumors containing subtypes with only very few CNAs [56, 57].

In summary, frequent and rare gene CNAs are both important for the prediction of survival impacts. Overall there is no general trend that frequent gene CNAs tend to be more important than rare gene CNAs for the prediction of patient survival. The contributions of patient-specific rare and frequent gene CNAs tend to be rather tumor type-specific.

## Conclusions

Multiple mutational patterns can perturb molecular pathways in similar ways leading to clinically almost indistinguishable phenotypes [1]. Thus, although the number of cellular endpoints that have to be altered is limited [6], the space of possible mutational patterns affecting the aggressiveness of a tumor (and ultimately patient survival) is practically unlimited. As a corollary of that, frequency-based approaches for detecting clinically relevant mutations will be capable only of detecting the mountains, leaving much of the phenotypic variation unexplained [2]. This study demonstrates the feasibility of an alternative strategy: the impact of gene CNAs on the expression of signature genes can be predicted using large compendia of independent data. Importantly, gene–gene relationships inferred from such data are largely conserved across multiple tumor types and enable statistically significant predictions of in vivo expression levels of most genes. Thus, although the expression variation of individual regulators changes the activity of molecular sub-networks, the topology of regulatory relationships as such turns out to be remarkably robust across cell types [58]. Because of that, we were able to quantify the importance of gene CNAs for individual tumor risks leading to the observation that rare variants can be as important as frequent variants. Although this observation is not unexpected in light of recent research [1–3, 15], our framework allows us to specifically identify individual CNA-affected genes with a potentially high impact on survival. Importantly, the frequency at which a high-impact gene gets mutated seems to be determined by factors that are independent of its function or impact. Thus, the fact that some high-impact genes have higher CNA frequencies may simply be due to their placement in genomic regions that are more amenable for CNAs than others. Because of the higher CNA frequency in those regions, these genes will preferentially be selected during tumor evolution leading to increased average impacts of high-frequency CNAs. In short, impact does not affect frequency, but high frequency still correlates with high impact.

In addition, we noticed striking differences between tissues and between tumor types. For example, the correlation between CNA frequencies and genomic features was highly dependent on the tumor type. In addition, the importance of rare and frequent gene CNAs to distinguish between short and long patient survival was also highly tumor type-specific. Further, we found many survival impact genes that are well-established cancer genes in one tissue to be also mutated (with a large predicted impact) in other tumors. However, the CNA frequency in those new tissues was mostly low, explaining why many of these genes have not been detected as being relevant in those tumors before. These observations imply that tissue-specific factors such as chromatin state, cell-cycle rates, exposure to DNA-damaging agents, number of stem cell divisions, or even the expression of specific genes could considerably impact on mutational mechanisms [59–61] that in the end affect patient survival.

Our conclusions rest on two computational models: the first, CCTN, describes transcriptional regulatory relationships between genes in a tumor context, i.e. in fast proliferating cells, but independent of a specific tumor type. The second model predicts signature genes associated with patient survival given cohort-specific expression and survival data. Three lines of evidence suggest that these models are robust and predictive. First, CCTN was predictive on a large set of in vitro perturbation-expression measurements. Second, CCTN was predictive on in vivo tumor data from all TCGA cohorts that we tested. Third, the impact scoring (which integrates both models) was predictive for survival in four out of five independent clinical cohorts that were not used for any of the previous analyses, revealing the tumor type-specific contributions of rare and frequent gene CNAs for the separation into long- and short-lived patients. However, despite our efforts to validate the models using a wide range of external data, this study is just a proof of principle. Obviously, improved models will have to account for a much wider range of mutation types, consider epigenetic effects, and include non-coding genes. Further, CCTN was learned from cancer cell lines to exclude variations in tumor cell purity between tumor samples that may have caused spurious dependencies between genes. Clearly, the usage of cancer cell line data has also disadvantages in comparison to tumor samples. Cell lines may not always correctly reflect the in vivo situation in tumors due to limitations set by cell cultures. We have designed the CCTN-based impact computation such that only those genes whose expression can adequately be predicted in the respective tumor entity contribute to the impact estimate. Thus, our framework makes no statement about genes whose regulatory networks differ significantly between cell lines and tumors. The list of genes that can be included in this analysis is further restricted by the use of different experimental platforms that did not cover identical gene sets. In addition, the quality of the predicted survival signatures varied greatly between the different tumor types, which is in agreement with previous reports on the limited usability of TCGA gene expression data for the prediction of patient survival [28]. This variability is in part due to the different sizes of patient cohorts or inadequate follow-up time. Further, the complexity of the mutational patterns and the relevance of CNAs in particular for the etiology of a tumor entity may further contribute to the differences in the predictive power of CCTN.

Our study provides clear indications that personalized analyses of patient-specific gene CNA profiles are feasible. The potential impacts of each patient-specific rare and frequent gene CNA on clinically relevant signature genes can be determined. So far we have only analyzed the impacts of rare and frequent gene CNAs on survival, but our framework is much more general, enabling, for example, other studies that may focus on impacts of rare and frequent gene CNAs on cancer-relevant signaling pathways or molecular signatures associated with treatment resistance. In addition, our framework also allows us to pinpoint potential high-impact genes in large chromosomal regions or on chromosomes that are recurrently affected by deletions or amplifications. We have demonstrated this potential for the recurrent duplication of chromosome 7 in glioblastomas, suggesting additional driver genes apart from the known role of EGFR. Further, comparative analyses of single-gene tests and a related network-based approach clearly demonstrated the value of our approach. Thus, our framework enables us to study the impacts of rare and frequent gene CNAs. Since copy number changes play a role in many other diseases or genetic disorders (e.g. trisomy 21), we anticipate applications of our framework beyond other interesting applications in cancer research.

Future work yet has to establish the value of accounting for rare gene CNAs to improve diagnostic and therapeutic measures. Fortunately, the availability of a regulatory model facilitates the detection of genes that are commonly affected by different rare gene CNAs, which might open a window of opportunity for developing therapeutic strategies against such rare mutations.

## Methods

### Cancer cell line data for CCTN inference

We initially considered all 991 human cancer cell lines from the Cancer Cell Line Encyclopedia (CCLE) [17] and reconstructed hybridization images of corresponding gene expression and aCGH microarrays to systematically screen for and remove all cancer cell lines with hybridization artifacts. This resulted in a cancer cell line data set of 768 cell lines from 24 primary tumor sites (Additional file 2: Table S1). We normalized the gene expression experiments using GCRMA [62] in combination with a BrainArray design file (HGU133Plus2_Hs_ENTREZG 15.0.0). The resulting gene expression levels of each cancer cell line were further standardized by subtracting the corresponding average gene-specific expression level of all cell lines leading to log-ratios. We removed genes that did not show any variation in gene expression across all cancer cell lines leading to 15,942 genes that were finally considered. The corresponding gene copy number data of all cancer cell lines were taken from CCLE. The copy number of a gene in a cell line was given by the log-ratio of the gene-specific copy number measured in the cell line in comparison to a normal reference.

### CCTN inference

We divided the genome-wide transcriptional regulatory network inference problem into independent gene-specific sub-network inference tasks to obtain CCTN. For each target gene *i*∈{1,…,*N*}, we assume that the expression level *e*_*id*_ of gene *i* in a cancer cell line *d*∈{1,…,*D*} can be predicted by a linear combination,

1 $$\begin{array}{*{20}l}  e_{id} = a_{ii} \cdot c_{id} + \sum_{j \ne i} a_{ji} \cdot e_{jd}, \end{array} $$

of the gene-specific CNA *c*_*id*_ and the expression levels *e*_*jd*_ of other potential regulator genes *j*≠*i*. The unknown parameters of this gene-specific linear model are specified by $\vec {a}_{i} := (a_{1i}, \dots, a_{Ni})^{\mathrm {T}} \in \mathbb {R}^{N}$. Here, *a*_*ii*_ quantifies the direct local gene copy number effect and *a*_*ji*_ with *j*≠*i* specifies the impact of the expression level of gene *j* on the expression level of gene *i*. The integration of gene-specific copy number data into the linear model extends gene expression-based correlation network inference approaches [19] and contributes to predicting the directionality of regulatory effects. We assume that a CNA of a regulator gene can lead to an altered expression of the regulator. This altered regulator expression can further lead to expression changes of target genes of the mutated regulator. Thus, each model parameter *a*_*ji*_ has a straightforward interpretation: (1) *a*_*ji*_<0 implies that the putative regulator *j* is associated with the repression of target *i*, (2) *a*_*ji*_>0 implies that the putative regulator *j* is associated with the activation of target *i*, and (3) *a*_*ji*_=0 implies that no putative regulatory edge between *j* and *i* exists.

All unknown parameters of the gene-specific linear model can be learned from the gene expression and gene copy number data of the 768 curated CCLE cancer cell lines. The use of cancer cell line data (which is free of normal cells) circumvented the variation in tumor cell purity between tumor samples that could lead to a spurious correlation between CNAs and expression levels of affected genes. Obviously, using cell line data also has disadvantages compared to data from tumors. For example, cell lines may incorrectly reflect the in vivo situation. However, our in-depth validation on TCGA tumor data suggests that the regulatory relationships are strongly conserved between cancer cell lines and patient-specific tumors (Fig. 2 e and f; Additional file 1: Figures S4, S5). We utilized lasso regression [18] to compute a sparse solution for the linear model in Eq. (1). Lasso minimizes the residual sum of squares,

2 $$ \begin{aligned} \vec{a}_{i}^{*} = \underset{\vec{a}_{i}}{\text{argmin}}\ \sum_{d=1}^{D} \left(e_{id} - \left(a_{ii} \cdot c_{id} + \sum_{j \ne i} a_{ji} \cdot e_{jd} \right) \right)^{2} + \ \lambda_{i}\sum_{j=1}^{N} |a_{ji}|, \end{aligned}   $$

of the measured expression *e*_*id*_ of gene *i* and the model-based predicted expression of gene *i* under consideration of all cancer cell lines in dependency of a fixed complexity parameter *λ*_*i*_≥0. The complexity parameter *λ*_*i*_ determines the amount of shrinkage of the individual model parameters *a*_*ji*_ toward zero, where larger values of *λ*_*i*_ lead to greater shrinkage. This also enables us to select relevant predictors (gene-specific copy number impact and regulator genes) that best explain the expression of the response gene, because irrelevant model parameters can be shrunk to zero. The values of the fitted model parameters depend on the choice of the gene-specific complexity parameter. We utilized the R package glmnet [63] to determine an optimal gene-specific complexity parameter and corresponding optimal model parameters. We determined *λ*_*i*_ by averaging the optimal complexity parameters (cv.glmnet: lambda.min) obtained from ten independent repeats of a tenfold cross-validation across all cancer cell lines. We then used this gene-specific complexity parameter *λ*_*i*_ to compute the corresponding optimal model parameters $\vec {a}_{i}^{*}$ considering all cancer cell lines. We further determined the significance of model parameters when they first enter the lasso model in Eq. (2) using a recently developed significance test for lasso [21]. This provides an efficient way to get *p* values instead of using computationally expensive permutation strategies. To realize this, we first computed the lasso solution paths for the active predictors (model parameters in $\vec {a}_{i}^{*}$ that are unequal to zero) with respect to all cancer cell lines using the R package lars [64]. These results were then evaluated using the R package covTest [65] to obtain *p* values that characterize the importance of individual active predictors in the gene-specific linear model.

The *p* value distributions of active predictors and a quantile–quantile plot are shown in Additional file 1: Figure S24a and b for ten learned CCTN instances. We observed a strong enrichment of non-significant *p* values close to one and a smaller peak for highly significant *p* values with values close to zero. *p* values between these two extremes tended to be uniformly distributed. This highly left-skewed *p* value distribution (strong enrichment of non-significant *p* values) favors the parsimony of the model and is expected from the mathematical theory behind the significance test for lasso [21] (see Additional file 1: Text S5 and Figure S24c and d for details). Thus, as expected for lasso-based network inference, only very few predictor genes are required for the prediction of the expression levels of specific response genes, whereas the majority of predictors shrink to zero. Note that the selected predictors remained significant after correction for multiple testing (Additional file 1: Figure S24e). Thus, regularization via lasso (reduction of the potential predictor test space) followed by additional filtering based on the significance of individually selected predictors represents an appropriate strategy to account for multiple testing.

We further removed all potentially selected local chromosomal regulator genes that were 50 genes upstream or downstream of each target gene to avoid the inclusion of genes that may simply reflect the copy number state of the target gene rather than regulatory dependencies. The choice of the local predictor cutoff is motivated by the observation that local chromosomal correlations of gene expression levels quickly approach zero with increasing distance between genes (Additional file 1: Figure S25a). Further, the structure of CCTN was hardly affected by varying local gene predictor cutoffs considering 20, 50, or 80 genes upstream or downstream of each response gene (Additional file 1: Figure S25b and c). Importantly, removing local chromosomal predictors did not affect the CCTN prediction accuracy, which was stable for the varying local predictor cutoffs (Additional file 1: Figure S25d–f). We just note that one could replace the fixed cutoff by a nucleotide distance cutoff to account for differences in local gene density, but as shown in Additional file 1: Figure S25, this will not have a strong influence on the results of our study.

We further tested if our network inference approach was affected by the multicollinearity of predictors by computing variance inflation factors (Additional file 1: Figure S26). Collinearity is present when two or more of the response gene-specific predictors have highly correlated measurements. The vast majority of variance inflation factors were close to one. Only 0.16 % of the predictor combinations had a variance inflation factor greater than ten, which is considered as an indicator of high multicollinearity [66]. Thus, CCTN is not affected by multicollinearity.

We repeated the learning of each gene-specific linear model ten times to evaluate the stability of our approach. We observed only very little variation of the gene-specific optimal complexity parameter, the gene-specific root mean square error, and the selected gene-specific predictors across the ten independent runs (Additional file 1: Figure S27a–c). We further selected for each target gene only those gene-specific predictors that had *p*<5×10^−5^ (standard numerical precision limit of the R package covTest) in all ten runs (Additional file 1: Figure S25d and e). This corresponds to a *q* value cutoff of 0.0032. Note that also other cutoffs can be used, but we specifically focused on the resulting most parsimonious network, which reached substantially better predictive power than more complex network instances. In more detail, the prediction accuracy of the resulting ten instances of the gene-specific linear model was highly similar considering the CCLE data (Additional file 1: Figure S27f). The resulting reduced gene-specific linear models also showed significantly improved prediction accuracies for all independent TCGA patient cohorts compared to the initially obtained linear models, which also included non-significant predictors (Fig. 2 e; Additional file 1: Figures S4–S6). Further, these predictions were also significantly better than the predictions of ten random networks of the same complexity as CCTN derived by degree-preserving permutations obtained by randomly exchanging predictors between the reduced gene-specific linear models while keeping the number of incoming and outgoing regulatory links constant for each gene (Fig. 2 e). All subsequent analysis was based on average predictions done by an ensemble of ten CCTN instances focusing on significant predictors. The computation of a CCTN instance was computationally demanding and could not be realized on a standard desktop computer. It took on average 13.03 ± 3.06 min to learn the parameters of a gene-specific linear model from the 768 CCLE cancer cell lines (AMD Opteron 6274, 2.2 GHz, 2 GB RAM). Thus, it would take more than 140 days to obtain the whole network for the 15,942 genes in a sequential approach. We, therefore, solved the independent regression problems in parallel on a high-performance computing cluster (HPC Atlas Cluster TU Dresden, AMD Opteron 6274).

Generally, the network inference is very time-consuming because of the large number of potential gene-specific regulators and the large number of samples that should be considered to obtain robust networks. So far, we have removed only genes with constant expression levels among all cancer cell lines to reduce the number of potential predictors. This could be further extended by removing genes that show only little variation of expression levels for all cancer cell lines. Additionally, a preselection of potential gene-specific predictors via a correlation analysis could further help to reduce the predictor space to reduce the global computation time. However, such potential future preselection steps should be done carefully to avoid the loss of predictive power, because in our final network, about 61 % of all genes were selected as potential regulators of other genes.

### Tumor data for validation, survival signature prediction and CNA impact studies

We downloaded gene expression and gene copy number data of 13 different tumor cohorts (4548 tumor patients in total) from TCGA [23]. Additional file 5: Table S4 contains all patient identifiers and dates of data freezes for the individual cohorts. We reorganized these data sets to obtain for each patient the corresponding gene expression levels and gene copy numbers for the 15,942 genes considered in the CCLE cancer cell line data set. To obtain gene-specific copy number log-ratios for each tumor patient, we mapped the tumor-specific aCGH segments to the corresponding genes. If segment breaks occurred within a gene, we used the average log-ratio of the involved segments as a gene-specific copy number measurement. If a gene was not covered by at least one aCGH segment, we assumed that this gene was not affected by a copy number change and set its corresponding gene copy number measurement to zero. Note that personal normal aCGH controls were not available from TCGA. Instead, copy number signals were normalized against a universal reference. Thus, it is not possible to distinguish germ-line gene CNVs from somatic gene CNAs. This, however, does not affect our impact estimates, since our estimates do not require any enrichment of somatic mutations at driver genes. Microarray gene expression data were already reported by TCGA as log-fold changes against a universal reference. For RNA-seq data, we computed log-fold changes by normalizing to the average expression of the given gene in a cohort. Generally, genes that were measured in the CCLE data set used for CCTN inference, but which were not measured in some TCGA cohorts due to different experimental platforms, were always included with artificial measurements of zero, which did not provide any information for CCTN. This was done to enable a standardized application of CCTN to the different cohorts. We finally added corresponding patient survival information (status: dead or alive; survival time; and follow-up time) from TCGA.

We further downloaded gene expression, gene copy number, and survival data of five additional tumor cohorts (292 tumor patients in total) to validate the whole CCTN impact scoring pipeline based on tumor data that were not used in any analysis before. We considered independent GBM patients from the Rembrandt repository [30], curated and standardized in [67]. We downloaded processed data of independent LUAD patients form the CLCGP cohort [55]. We further downloaded newly added patients for the TCGA cohorts HNSC, LUAD, and SKCM and processed them as described above. Corresponding patient identifiers and dates of data freezes of all cohorts are provided in Additional file 5: Table S4.

### CCTN-based impact computation

We developed a two-step approach to predict the impact of a specific gene CNA on the expression of a target gene of interest (here, signature genes) using CCTN, which represents regulatory relationships between genes learned from CCLE data. We now use CCTN to infer a cohort- or patient-specific impact matrix by propagating effects through CCTN using its regulatory paths between genes. Importantly, the resulting impact score is corrected for the variance that can be explained by CCTN at each node (gene) on the paths from the CNA gene to the target. An alternative naive approach would have been to correlate CNA states of genes directly with the expression of target genes of interest. Such an approach, however, has several disadvantages. First of all, such a model would be unable to predict the effects of CNAs that were not already contained in the training data, rendering it basically useless to investigate the effects of rare CNAs. Our approach can predict the effects of CNAs that were not seen in the specific patient cohort before. Second, the naive correlation model would lack mechanistic detail about how effects are propagated through the network, which is important for the interpretation of the results.

#### Basic network propagation algorithm

For all these reasons, we developed a network propagation algorithm that utilized CCTN to compute the information flow between genes in the network. This allowed us to compute the impact of patient-specific gene CNAs on survival signature genes. We considered a given TCGA cancer cohort of *D* patients for which gene expression and gene copy number profiles were measured for *N* genes. For each patient *d*∈{1,…,*D*}, we took its gene expression and gene copy number profile to predict the expression level *e*_*id*_ of each gene *i*∈{1,…,*N*} using the corresponding gene-specific linear model in Eq. (1) with optimal parameters $\vec {a}_{i}^{*}$ from CCTN. Next, we computed each gene-specific correlation coefficient *r*_*i*_ between the predicted and the originally measured expression levels of gene *i* across all *D* patients of that cohort. Subsequently, we analyzed only genes with a positive correlation between predicted and observed expression levels (*r*_*i*_>0), and we termed those genes predictable. The fraction of predictable genes varied between tumors types (Additional file 1: Figure S5). Note that poorly predictable genes (i.e. genes with small positive *r*_*i*_) will contribute only very little to the total impact score (see below). Thus, it is not necessary to further increase the minimal *r*_*i*_ for calling predictable genes. Next, we computed the corresponding variance ${R_{i}^{2}} = r_{i} \cdot r_{i}$ explained for predictable genes that was covered by the underlying linear model in Eq. (1) and set ${R_{i}^{2}} := 0$ for unpredictable genes (*r*_*i*_<0). Thus, ${R_{i}^{2}}$ directly reflects the network-based prediction accuracy for the expression level of gene *i* under CCTN by quantifying to what extent CCTN can explain the variance of gene *i* in a specific cancer cohort. Next, we considered each regulator gene *j* of gene *i* and determined for each regulator its direct contribution to the observed explained variance ${R_{i}^{2}}$ of gene *i*. Therefore, we computed the average proportion of each regulator *j* on the prediction of the expression of target gene *i* by

$$\begin{array}{*{20}l} p_{ji} &= \frac{1}{D}\sum_{d=1}^{D} \frac{|a_{ji} \cdot e_{jd}|}{|a_{ii} \cdot c_{id}| + \sum_{v \ne i} |a_{vi} \cdot e_{vd}|} \end{array} $$

and determined the direct average copy number contribution of target gene *i* by

$$\begin{array}{*{20}l} p_{ii} &= \frac{1}{D}\sum_{d=1}^{D} \frac{|a_{ii} \cdot c_{id}|}{|a_{ii} \cdot c_{id}| + \sum_{v \ne i} |a_{vi} \cdot e_{vd}|} \end{array} $$

under consideration of the *D* patients. We used absolute values in the computation of *p*_*ij*_ (and *p*_*ii*_) to account for regulator genes that act as either inhibitors or activators of target gene *i*. If a gene *j* is not a direct regulator of gene *i* (*a*_*ji*_=0), then *p*_*ji*_ is set to zero. In analogy, if target gene *i* does not have a direct copy number effect (*a*_*ii*_=0), then *p*_*ii*_ is set to zero. Based on that, we defined a basic network flow matrix,

$$ F = \left(\,f_{ji} \right)_{1 \le j,i \le N} := p_{ji} \cdot {R_{i}^{2}}, $$

by weighting the explained variance ${R_{i}^{2}}$ of target gene *i* with the average proportion *p*_*ji*_ of its direct predictors (gene copy number and regulator genes) *j*. Thus, each column *i* of *F* contains the explained variance of a target gene *i* split into average proportions according to the contributions of its copy number and its target gene-specific regulators. The prediction of gene expression levels in tumors is of good quality, but of course not perfect (Additional file 1: Figure S5). For that reason, the explained variance fulfills $0 \le {R_{i}^{2}} < 1$ and, thus, the column sum norm of *F* is strictly less than one. We utilized this to compute the indirect effects of gene CNAs on other genes (i.e. the network flow) via:

$$ F^{*} = \sum_{k=1}^{\infty} F^{k}, $$

which sums over the contributions of all network paths of increasing length *k*. Here, *F* ^*k*^ specifies the *k*th matrix power obtained by a *k*-fold matrix multiplication of *F*. An element $f_{ji}^{k}$ of *F* ^*k*^ represents the impact of a trans-acting regulator gene *j* on the explained variance of a target gene *i* via all directed network paths from *j* to *i* of length *k*. Since the basic network flow matrix *F* has a column sum norm that is strictly less than one, the network flow *F* ^∗^ will converge to its limit (*I*−*F*)^−1^−*I* (geometric series of matrix *F* starting at one), where *I* is the identity matrix and (*I*−*F*)^−1^ specifies the inverse of matrix *I*−*F*. However, the computation of the inverse of a large matrix is very time-consuming (*I*−*F* has dimension *N*×*N*). In addition, due to the sparsity of *F* (the majority of entries are zero because CCTN utilizes only the most relevant predictors) and its entries in [0,1), we also know that the values of the elements in *F* ^*k*^ quickly approach zero. Thus, it is more efficient to approximate *F* ^∗^ by adding only an additional *F* ^*k*^ if the obtained difference of the sum over *F* ^*k*^ up to *k* and the previous sum up to *k*−1 is greater than a predefined threshold. We stopped the approximation of *F* ^∗^ if the sum of the differences of the column sums of the current and the previous approximated matrix was less than 10^−3^. Starting with a TCGA cohort-specific sparse initial basic flow matrix *F*, we typically reached convergence after less than 50 iterations for most of the 13 different TCGA cohorts. The resulting network flow matrix *F* ^∗^ represents the impact values for each gene pair. All impact values in *F* ^∗^ are equal to or greater than zero. We further standardized each column of *F* ^∗^ by dividing each column-specific impact entry by the total sum of column-specific impacts followed by multiplication by 100 to get impact values in percentages. The impact of a gene *j* on the variation of expression of a gene *i* is given by $f_{ji}^{*}$. By considering the corresponding entries of *F* ^∗^, we were able to quantify the impact of each patient-specific gene CNA on the predicted TCGA cohort-specific survival signature genes.

#### Identification of gene CNAs with a high impact on survival

We applied the basic network propagation algorithm to all TCGA cohorts for which we obtained survival signature genes that were significantly associated with patient survival (AML, GBM, HNSC, LUAD, OV, and SKCM). This resulted in a cohort-specific impact matrix *F* ^∗^ for each cohort. We next determined for each patient in a cohort all of their tumor-specific gene CNAs (genes with absolute aCGH log-ratio ≥0.75; Additional file 1: Figure S17: results obtained for a more stringent absolute aCGH log-ratio cutoff ≥1 were highly similar) and computed the frequency of all gene-specific deletions or duplications in the whole cohort. We then took each mutated gene and considered the cohort-specific impact matrix *F* ^∗^ to compute the average impact that a mutated gene had on all cohort-specific survival signature genes (Additional file 1: Figure S12: selection of a stringent set of signature genes using a correlation cutoff >0.1; Additional file 1: Figure S13; and Additional file 1: Figure S17: results obtained for a less stringent correlation cutoff >0.05 were highly similar). We next considered for each cohort all genes that had at least one deletion or duplication, sorted all these genes in increasing order of their impacts, computed the cumulative impact across all mutated genes, and plotted this cumulative impact clearly highlighting cohort-specific gene CNAs with a high impact on patient survival (Additional file 1: Figure S15). We next used a cumulative impact cutoff of greater than one to select high-impact gene CNAs for each cohort (Additional file 1: Figure S15: black dashed line close to zero). We further ensured that the impact of each selected high-impact gene on the survival signature genes was significantly greater than the corresponding gene-specific impacts obtained under ten random networks of the same complexity as CCTN (degree-preserving network permutations). Therefore, we computed for each CNA gene in a cohort the difference between its CCTN-based impact score and each corresponding impact score under a random network leading to ten gene-specific impact score differences per gene. We then tested for each gene if the gene-specific differences between the original and the random impact scores were greater than zero using a one-sided Wilcoxon test. We further corrected the resulting *p* values for multiple testing by computing false-discovery-rate-adjusted *p* values (*q* values) for all genes [68]. We recognized that also very small impacts close to zero can be highly significant, because the observed impacts obtained under random networks were even closer to zero. However, such genes with very small impact are less likely to be biologically or clinically relevant. Therefore, we decided to focus only on stringent selections of cohort-specific high-impact genes based on Additional file 1: Figure S15 as described above instead of using a fixed *q* value cutoff. The *q* values of the selected high-impact genes were less than 0.006 for all TCGA cohorts (*q* value cutoffs: AML < 0.0053, GBM < 0.0048, HNSC < 0.0058, LUAD < 0.0056, OV < 0.0046, and SKCM < 0.0049).

#### Extension to patient-specific impact scores

Further, we note that the proportions *p*_*ji*_ and *p*_*ii*_ used to construct the basic network flow matrix *F* are cohort-specific averages using the basic network propagation algorithm described above. One can easily modify the computation to get specific proportions for each individual tumor patient (Additional file 1: Text S1: Patient-specific absolute impact scores) to construct a patient-specific basic network flow matrix *F*, but these computations and the later network propagation steps are even more time- and resource-consuming because one now has to apply the network propagation algorithm to each individual patient. This takes about 24 hours on an AMD Opteron 6274 with 2.2 GHz, requiring up to and more than 80 GB RAM for one patient. A compressed basic network flow matrix *F* required about 1 MB of disk storage for one patient, but the resulting compressed final impact matrix *F* ^∗^ required about 1 GB of hard disk space. To compare both approaches, we randomly selected 100 patients from each of the six TCGA cohorts (AML, GBM, HNSC, LUAD, OV, and SKCM) and found that the obtained patient-specific impact values acting on survival signature genes or all network genes are strongly correlated with the corresponding cohort-specific impact values (Additional file 1: Figure S28). For that reason, we decided to work with cohort-specific impact scores in Figs. 4, 5, 6 and 7 and the corresponding Additional file 1: Figures S15–S20. We did notice, however, that in some cases the patient-specific impact matrix significantly deviated from the cohort average (Additional file 1: Figure S28), suggesting that in the future, it might even be worthwhile to use personalized impact matrices.

In addition to this absolute quantification of impacts of patient-specific gene CNAs, one can further slightly modify the computation of the specific proportions *p*_*ji*_ and *p*_*ii*_ to obtain relative proportions that enable us to propagate patient-specific repressive and activating impacts through the network (Additional file 1: Text S1: Patient-specific relative impact scores). We used these scores to compute patient-specific survival impact scores considering all corresponding tumor-specific gene CNAs as described in ‘Results and discussion’. These patient-specific survival impact scores enabled us to distinguish between long- and short-lived patients and to investigate the contributions of all, frequent, or rare tumor-specific gene CNAs on patient survival (Fig. 9; Additional file 1: Figures S14, S22, and S23). This approach was as time and resource intensive as described above.

### Perturbation data for CCTN-based impact validation

We used the L1000 data set of the Library of Integrated Network-based Cellular Signatures (LINCS) [24] to validate our CCTN-derived impact scores. The L1000 data set provides information about gene expression changes of different human cell lines in response to chemical (small molecule) or genetic (shRNA) perturbations. We focused on perturbation experiments done for the about 1000 landmark genes defined by the LINCS consortium as representatives of the human transcriptome. We found that 933 of these landmark genes were part of CCTN. We next considered all gold standard perturbation experiments performed for these 933 genes and downloaded for each perturbation experiment the corresponding accessible top 100 response genes (top 50 up- and top 50 down-regulated landmark genes) via the application programming interface accessible under http://api.lincscloud.org/. Overall, we obtained the top 100 response genes of 50,306 perturbation experiments leading to on average 54 perturbation experiments for each of the 933 genes (Additional file 6: Table S5). We used this information to create a response gene frequency statistic for each perturbed gene by taking into account all corresponding gene-specific perturbation experiments, i.e. we counted how frequently each of the 933 landmark genes was observed among the top 100 response genes. Next, we compared the ranks of the corresponding impact scores from the CCTN-derived impact matrix with these independently obtained response scores. CCTN-derived impact scores and LINCS-derived response scores were correlated gene-wise. The distribution of *p* values resulting from a pan-cancer analysis of the individual impact matrices obtained for the 13 different TCGA cohorts was significantly shifted towards small values [Fig. 2 f, one-sided Kolmogorov–Smirnov test comparing the *p* value distribution of the forward model (see below) to a uniform distribution representing the baseline for non-significant enrichment: *p*<2.1×10^−23^ for each TCGA cohort], confirming the overall significant predictive power of our impact scores. Importantly, such a significant shift towards small *p* values was also observed for each individual impact matrix of a TCGA cohort (Additional file 1: Figure S8).

In addition, for the perturbation experiments, the directionality of effects is known. Thus, we utilized the LINCS data to validate the correct prediction of the directionality of effects by CCTN. Therefore, we compared the standard forward model, which quantifies the significance of correlations between computed impacts flowing from a perturbed regulator to its targets and the corresponding experimentally measured impacts, to the reverse model, which quantifies the significance of correlations between computed impacts flowing in the reverse direction from the responding targets to their perturbed regulator and experimentally measured forward impacts. That means that in the forward model, both compared impacts flow in the same direction, whereas in the reverse model, the compared impacts flow in opposite directions. If CCTN contained only information about pairwise correlations of gene expression levels, we would expect that the forward and the reverse models would perform equally well on the LINCS data. We found that the forward model reached a stronger enrichment of small *p* values than the reverse model for a pan-cancer analysis of the individual impact matrices obtained for the 13 different TCGA cohorts (Fig. 2 f, one-sided Kolmogorov–Smirnov test comparing the *p* value distribution of the forward model to the *p* value distribution of the reverse model: *p*<0.015 for each TCGA cohort). This was also found for the impact matrix of each individual TCGA cohort (Additional file 1, Figure S8a–m) and further supported by direct gene-specific comparisons of the forward and backward models (Additional file 1: Figure S8o). This suggests that CCTN is mostly able to correctly predict the directionality of effects.

### Identification of survival signature genes

We used random forest (RF) [26] to identify genes that were associated with the survival of patients in TCGA cohorts. RF was previously found to be one of the best performing methods for the prediction of patient survival based on gene expression data [27]. All analyses were performed on uncensored data using the R package randomForest [69] with standard settings. We initially applied RF to patient-specific gene expression profiles of each TCGA cohort containing more than 20 patients with survival information (Additional file 5: Table S4: AML, BRCA, GBM, HNSC, LUAD, LUSC, OV, and SKCM) to evaluate how many patients are required for significant predictions of patient survival. Validations of each cohort-specific RF on corresponding out-of-the-bag data (patient-specific gene expression profiles that a specific tree of the RF has not seen during its construction) showed that for six TCGA cohorts with more than 100 patients (AML, GBM, HNSC, LUAD, OV, and SKCM), significant predictions of patient survival were possible (one-sided correlation tests: *p*<0.1; Additional file 1: Figure S9).

Next, we focused on these six cohorts and developed an RF-based approach to determine genes that are associated with patient survival. For each of the selected TCGA cohorts, we standardized the expression levels of each gene to a mean of zero and a standard deviation of one across all patients. We next randomly selected 90 % of the patients for the training of an RF and utilized the remaining patients as independent test sets for the evaluation of the performance of survival prediction and the characterization of relevant genes. We trained an RF on the training set and determined the corresponding gene-specific selection frequencies (SFs) that quantify how frequently each gene was chosen as a relevant survival predictor. We repeated the separation into training and test data 100 times and trained the corresponding RFs to evaluate the stability of the obtained SFs. We found that the standard deviations of the SFs were close to zero also for genes with SFs clearly greater than zero. Thus, the RF-based association of genes with patient survival was robust. Next, we computed the average SF for each gene based on the 100 RFs and corrected them for selection biases. This was done by subtracting average gene-specific SFs obtained from 100 corresponding RFs that were trained using randomly permuted survival information. To obtain a ranking of genes with respect to their strength of association with patient survival, we ranked all genes in decreasing order of their average corrected SFs. This allowed us to quantify their importance for the prediction of patient survival utilizing the independent test data set that we had initially put aside. Therefore, we considered each of the 100 RFs and its corresponding test data set and predicted the survival of the test patients with respect to successively increasing numbers of permuted expression levels (permutation of gene-specific expression levels across all test patients) for the previously determined top-ranking predictor genes. For each of these successive permutation steps, we computed the correlation between the originally observed test patient survival and the RF-predicted test patient survival to quantify the importance of the top-ranking genes associated with survival. We repeated this procedure ten times for each of the 100 RFs leading to 1000 permutation runs in total. We did this in steps of single genes for the first 1000 top-ranking predictors followed by steps of 100 genes for the remaining top-ranking predictors. We finally averaged the obtained correlation profiles for successively permuted top-ranking predictors across the 1000 permutations. We found that the average correlation profile of the top-ranking predictors quickly approached zero, enabling us to set a cutoff to select the most relevant genes associated with survival (Additional file 1: Figure S12). We subsequently considered all predictor genes above a stringent correlation cutoff of 0.1 (also later used in our in-depth studies) and a less stringent cutoff of 0.05 as TCGA cohort-specific survival signature genes and confirmed that the expression of these genes was correlated with patient survival.

Therefore, we used standard hierarchical clustering to group the top-ranking predictor genes revealing two major groups: (1) survival signature genes negatively associated with survival and (2) survival signature genes positively associated with survival. We finally computed average patient-specific gene expression levels for these two clusters and confirmed that these average expression profiles are significantly correlated with patient survival (one-sided correlation tests: *p*<0.05; Additional file 1: Figure S13), suggesting that our RF approach is well suited for the identification of survival signature genes.

In addition, we also compared our RF approach to random survival forest (RSF) [29], which can handle right-censored data to gain additional information for the prediction of patient survival. We used the corresponding R package randomForestSRC to determine RSFs. We found that our RF approach reached clearly better predictions of patient survival than RSF without and with censoring for the initially considered TCGA cohorts (Additional file 1: Figure S10). See ‘Results and discussion’ for more details.

### Gene annotations and genomic features

Lists of human transcription factors and co-factors, phosphatases, kinases, signaling and metabolic pathway genes, essential genes, tumor suppressor and oncogenes, and known cancer genes were compiled from different public resources (see Additional file 9: Table S8 for genes and references). Fragile genomic sites [53] were extracted and lifted over to hg19 (Additional file 10: Table S9). Frequently observed CNV sites [54] were available for hg19 (Additional file 11: Table S10).

## Additional files

Additional file 1
**Texts S1–S5 and Figures S1–S28.** (PDF 6430 kb)

Additional file 2
**Table S1.** Cancer cell lines used. (TXT 62 kb)

Additional file 3
**Table S2.** CCTN gene interaction table. (TXT 966 kb)

Additional file 4
**Table S3.** CCTN node degrees. (TXT 1177 kb)

Additional file 5
**Table S4.** Patient samples used. (XLS 278 kb)

Additional file 6
**Table S5.** LINCS perturbation experiments used. (TXT 1054 kb)

Additional file 7
**Table S6.** Predicted TCGA cohort-specific survival signature genes. (XLS 58 kb)

Additional file 8
**Table S7.** Gene CNA rates and survival impacts. (XLS 4259 kb)

Additional file 9
**Table S8.** Gene annotations table. (XLS 3420 kb)

Additional file 10
**Table S9.** Fragile sites used. (TXT 2 kb)

Additional file 11
**Table S10.** Germ-line CNV sites used. (TXT 17 kb)

## Abbreviations

AML: Acute myeloid leukemia
BRCA: Breast invasive carcinoma
CCLE: Cancer cell line encyclopedia
CCTN: Cancer cell transcriptional network
CLCGP: Clinical lung cancer genome project
CNA: Copy number alteration
CNV: Copy number variation
COAD: Colon adenocarcinoma
GBM: Glioblastoma multiforme
HNSC: Head and neck squamous cell carcinoma
lasso: Least absolute shrinkage and selection operator
LINCS: Library of Integrated Network-based Cellular Signatures
LUAD: Lung adenocarcinoma
LUSC: Lung squamous cell carcinoma
OV: Ovarian serous cystadenocarcinoma
RF: Random forest
RSF: Random survival forest
SKCM: Skin cutaneous melanoma
SNV: Single nucleotide variation
STAD: Stomach adenocarcinoma
TCGA: The cancer genome atlas
THCA: Thyroid carcinoma
