# Supplementary material for: Importance of rare gene copy number alterations for personalized tumor characterization and survival analysis
Source: Genome Biol. 2016 Oct 3;17:204. doi: 10.1186/s13059-016-1058-1 (PMC5046221; doi:10.1186/s13059-016-1058-1)
Supplement: Additional file 1 — Texts S1–S5 and Figures S1–S28. (PDF 6430 kb) [file 13059_2016_1058_MOESM1_ESM.pdf]

Supplementary Texts and Figures

Importance of rare gene copy number alterations for personalized tumor characterization and survival analysis

Michael Seifert [1,2,4], Betty Friedrich [3] and Andreas Beyer [4]

[1]Carl Gustav Carus Faculty of Medicine, Technische Universität Dresden, Institute for Medical Informatics and Biometry, Fetscherstr. 74, D-01307 Dresden, Germany; [2]National Center for Tumor Diseases (NCT), Dresden, Germany; [3]Institute of Molecular Systems Biology, Auguste-Piccard-Hof 1, CH-8093, Zurich, Switzerland; [4]Cellular Networks and Systems Biology, CECAD, University of Cologne, Joseph-Stelzmann-Str. 26, D-50931, Cologne, Germany.

Contact: michael.seifert@tu-dresden.de

Contents

1 Supplementary Texts **3**

1.1 Text S1: Basics for CCTN-based impact computation . . . 3

1.1.1 Cohort-specific impact scores . . . 3

1.1.2 Patient-specific absolute impact scores . . . 3

1.1.3 Patient-specific relative impact scores . . . 3

1.2 Text S2: Number of patient-specific gene CNAs alone does not allow to predict survival . 4

1.3 Text S3: Single-gene tests widely fail to identify gene CNAs that impact on survival 4

1.4 Text S4: Impact scoring only utilizing directly acting gene CNAs classifies a reduced number of patients with less discriminative power . . . 5

1.5 Text S5: A strong enrichment of non-significant p-values is expected from the mathematical theory behind the significance test for lasso . . . 5

2 Supplementary Figures **7**

2.1 Figure S1: Tissue origins of considered CCLE cancer cell lines . . . 7

2.2 Figure S2: CCLE microarray hybridization artifacts . . . 8

2.3 Figure S3: Genome-wide visualization of CCTN . . . 9

2.4 Figure S4: CCTN predictions for independent TCGA cohorts . . . 10

2.5 Figure S5: CCTN predictions for individual TCGA cohorts . . . 11

2.6 Figure S6: Robustness of CCTN predictions for varying p-value cutoffs . . . 12

2.7 Figure S7: Comparison of CCTN to tumor type-specific networks . . . 13

2.8 Figure S8: Summary of LINCS-based validation of CCTN impact values . . . 14

2.9 Figure S9: RF-based prediction of patient survival for TCGA cohorts . . . 15

2.10 Figure S10: Comparison of RF to RSF without and with censoring . . . 16

2.11 Figure S11: RF-based prediction of Rembrandt GBM patient survival . . . 17

2.12 Figure S12: Selection of survival predictor genes for TCGA cohorts . . . 17

2.13 Figure S13: TCGA cohort-specific survival signatures . . . 18

2.14 Figure S14: Validation of entire pipeline on Rembrandt GBM cohort . . . 19

2.15 Figure S15: Selection of gene CNAs with high impact on survival . . . 20

2.16 Figure S16: Genomic locations of rare and frequent survival-impact genes . . . 21

2.17 Figure S17: Patient-specific survival risk curves . . . 22

2.18 Figure S18: General characterization of gene CNAs impacting on survival . . . 23

2.19 Figure S19: Impact of rare and frequent gene CNAs on survival 24
2.20 Figure S20: Identification of survival-associated gene CNAs using t-tests 25
2.21 Figure S21: Distance of survival impact genes from genomic features 26
2.22 Figure S22: Kaplan-Meier analyses 27
2.23 Figure S23: Kaplan-Meier analyses comparing CCTN to random networks 28
2.24 Figure S24: CCTN p-value and q-value distributions 29
2.25 Figure S25: Influence of local chromosomal predictors 31
2.26 Figure S26: CCTN variance inflation factors 32
2.27 Figure S27: Robustness of CCTN network inference 33
2.28 Figure S28: Patient-specific vs. cohort-specific impact scores 34

1 Supplementary Texts

1.1 Text S1: Basics for CCTN-based impact computation

1.1.1 Cohort-specific impact scores

In the methods section of the main manuscript, we have derived the average contribution of each regulator j on the prediction of the expression level of a target gene i by

$$p_{ji} = \frac{1}{D}\sum_{d=1}^{D}\frac{|a_{ji} \cdot e_{jd}|}{|a_{ii} \cdot c_{id}| + \sum_{v \neq i} |a_{vi} \cdot e_{vd}|}$$

and we have determined the direct average copy number contribution of target gene i by

$$p_{ii} = \frac{1}{D}\sum_{d=1}^{D}\frac{|a_{ii} \cdot c_{id}|}{|a_{ii} \cdot c_{id}| + \sum_{v \neq i} |a_{vi} \cdot e_{vd}|}$$

under consideration of all D patients of a given cohort. These proportions were used to define a basic cohort-specific network flow matrix $F = (f_{ji})_{1 \leq j,i \leq N} := p_{ji} \cdot R_i^2$ by weighting the CCTN-derived explained variance R_i^2 of target gene i in a given tumor cohort with the average proportions p_{ji} of its corresponding direct predictors j.

1.1.2 Patient-specific absolute impact scores

To obtain patient-specific impact scores, we have computed the patient-specific proportion of each regulator j on the prediction of the expression level of target gene i by

$$p_{ji}^{d} = \frac{|a_{ji} \cdot e_{jd}|}{|a_{ii} \cdot c_{id}| + \sum_{v \neq i} |a_{vi} \cdot e_{vd}|}$$

and we have determined the direct copy number contribution of target gene i by

$$p_{ii}^{d} = \frac{|a_{ii} \cdot c_{id}|}{|a_{ii} \cdot c_{id}| + \sum_{v \neq i} |a_{vi} \cdot e_{vd}|}$$

for each individual patient $d \in \{1, \ldots, D\}$ in a given tumor cohort. In analogy to the cohort-specific impact scores derived on the basis of p_{ji} and p_{ii} followed by the application of our network propagation algorithm, the use of absolute values in the computations of p_{ij}^{d} and p_{ii}^{d} does not allow to distinguish between opposed effects of repression and activation by different predictors (gene copy number, regulator genes) of target gene i, but instead allow to account for the absolute contribution of both effects to identify those tumor-specific gene CNAs with the strongest total impact on survival signature genes.

In analogy to the definition of the basic cohort-specific impact matrix above, we used the proportions p_{ij}^{d} and p_{ii}^{d} to define a basic patient-specific network flow matrix $F_d = \left(f_{ji}^{d}\right)_{1 \leq j,i \leq N} := p_{ji}^{d} \cdot R_i^2$. Here, R_i^2 again specifies the CCTN-derived explained variance R_i^2 of target gene i for a given tumor cohort accounting for the uncertainty of CCTN predictions as used previously for the definition of F. The resulting patient-specific basic network flow matrix F_d is then utilized to compute a patient-specific network flow matrix F_d^* using the network propagation algorithm described in the methods section of the main manuscript.

1.1.3 Patient-specific relative impact scores

A slight modification of the computation of the patient-specific proportions allows to obtain patient-specific impact scores that account for repressor and activator interactions in CCTN. This is important to quantify the putative regulatory effects of each individual tumor-specific gene CNA on signature genes.

To realize this, we computed the patient-specific proportion of each regulator j on the prediction of the expression of target gene i by

$$p_{ji}^d = \frac{a_{ji} \cdot e_{jd}}{|a_{ii} \cdot c_{id}| + \sum_{v \neq i} |a_{vi} \cdot e_{vd}|}$$

and we determined the direct copy number contribution of target gene i by

$$p_{ii}^d = \frac{a_{ii} \cdot c_{id}}{|a_{ii} \cdot c_{id}| + \sum_{v \neq i} |a_{vi} \cdot e_{vd}|}$$

for each individual patient $d \in \{1, \ldots, D\}$ in a given tumor cohort. The individual proportions now have a positive or negative sign that is derived based on the type of regulator j, which can either repress ($a_{ji} < 0$) or activate ($a_{ji} > 0$) the target gene i, under consideration of the corresponding patient-specific gene expression level e_{jd} and gene copy number measurement c_{id}. Thus, these proportions account for opposed effects of repressors and activators of the target gene i.

In analogy to the previous definitions, we used the proportions p_{ji}^d and p_{ii}^d to define a basic patient-specific network flow matrix $F_d := \left(f_{ji}^d\right)_{1 \leq j,i \leq N} := p_{ji}^d \cdot R_i^2$. Here, R_i^2 again specifies the CCTN-derived explained variance R_i^2 of target gene i for a given tumor cohort accounting for the uncertainty of CCTN predictions. Based on the definitions of p_{ji}^d and p_{ii}^d in combination with R_i^2, the column sum norm of F_d is still strictly less than one.

The resulting patient-specific basic network flow matrix F_d is then utilized to compute the network flow matrix F_d^* as described in the methods section of the main manuscript with a slight modification of the convergence criterion. Since we now account for opposed effects of repressive and activating impacts propagated through CCTN, the impacts accumulated during the approximation of F^* by taking into account paths of increasing lengths through CCTN are no longer guaranteed to increase monotonically until convergence is reached. Still, the absolute impact of a path of increasing length gets smaller, because one increases the path always by a multiplication with a proportion that is by definition in the interval $(-1, 1)$, but the sign of the added impact can change. Therefore, we stop the approximation of F_d^* if the sum of the absolute differences of the column sums of the current and the previously approximated matrix is less than $1 \cdot 10^{-3}$.

1.2 Text S2: Number of patient-specific gene CNAs alone does not allow to predict survival

We tested if a count statistic based on the number of patient-specific gene CNAs alone is sufficient to predict patient survival. Therefore, we determined the number of rare (frequency $<$ 1%) and frequent gene CNAs (frequency $>=$ 1%) for each patient in each of the six TCGA cohorts (AML, GBM, HNSC, LUAD, OV, SKCM). We did not find any significant correlation between the patient-specific number of gene CNAs (all gene CNAs, only frequent gene CNAs, or only rare gene CNAs) and survival for any cohort. In addition to the fact that such a frequency based approach would not allow to pinpoint specific genes, this also clearly indicated that the prediction of patient survival only based on the number of gene CNAs is not possible for the considered cohorts. This further motivated the application of our approach that allowed to identify patient-specific gene CNAs (out of the large set of patient-specific gene CNAs) that impact on survival.

1.3 Text S3: Single-gene tests widely fail to identify gene CNAs that impact on survival

We have shown that our approach allows to pinpoint rare and frequent gene CNAs that act on survival signature genes (Fig. 4) and that our approach can distinguish between short and long patient survival

(Fig. S14b). Next, we investigated if a basic statistical analysis based on single-gene tests would allow to identify differences in survival between patients with and without a specific gene CNA. Such a basic approach has at least three major limitations in comparison to our approach: (i) each specific gene CNA can only be considered in isolation independent of the other patient-specific gene CNAs, (ii) it will be almost impossible to analyze the impact of rare gene CNAs on survival due to the limited number of patients that have such a mutation, and (iii) potential regulatory links through which gene CNAs may act on survival remain hidden. However, we determined for each of the six TCGA cohorts (AML, GBM, HNSC, LUAD, OV, SKCM) all genes that had at least two gene CNAs within a cohort. We further treated deletions and amplifications separately and compared the survival of patients with and without a specific gene CNA using a t-test. We corrected for multiple testing by computing q-values and visualized the q-values with their corresponding gene CNA frequencies (Fig. S20). Only in the case of AML some frequent gene CNAs associated with differences in patient survival were identified (q-value < 0.01). Further, as expected, this basic approach did not allow to identify any significant rare gene CNA that acted on survival. Thus, this further motivated the application of our approach to pinpoint rare and frequent gene CNAs that act on patient survival.

1.4 Text S4: Impact scoring only utilizing directly acting gene CNAs classifies a reduced number of patients with less discriminative power

In addition, we compared our CCTN-based impact quantification approach, which considers all patient-specific gene CNAs that directly or indirectly act on survival signature genes, to a basic CCTN-based version. This basic version only considers CNAs of regulator genes in the direct network neighborhood of survival signature genes to distinguish between short and long-lived patients. This is computationally realized by only utilizing the basic network flow matrix F_d (direct impacts between gene pairs) instead of the final network flow matrix F_d^* (direct and indirect impacts between gene pairs) obtained via network propagation. Details to the computation of both matrices are given in the Methods section of the main manuscript and in Text S1. Thus, except for the quantification of indirect impacts, both impact quantification approaches were identical. This enabled a fair comparison to quantify the importance and additional contribution of gene CNAs that indirectly act on survival signature genes via regulatory paths defined by CCTN. We compared both impact scoring approaches on five independent test cohorts and found that our CCTN-based impact quantification approach that integrates directly and indirectly acting gene CNAs significantly improved the number of classifiable patients and the separation between short and long-lived patients (Fig. 8). Details are given in the Results section of the main manuscript.

1.5 Text S5: A strong enrichment of non-significant p-values is expected from the mathematical theory behind the significance test for lasso

The highly left-skewed p-value distributions (strong enrichment of non-significant p-values close to one) in Fig. S24a are explained by the mathematical theory behind the significance test for lasso (Lockhart et al. (2014)). The underlying covariance test statistic asymptotically follows an exponential distribution Exp(1) with scale parameter one. Further, the comparison of the test statistic along the lasso path to this exponential distribution is increasingly conservative. In more detail, consider a set of N predictors of which the first predictors $\{1, \ldots, n\}$ are the truly active ones, whereas the remaining predictors $\{n + 1, \ldots, N\}$ are the truly non-active ones. It was shown in Lockhart et al. (2014) that the covariance test statistic of each truly active predictor follows an Exp(1), whereas the test statistic of each truly non-active predictor $i \in \{n + 1, \ldots, N\}$ follows an Exp(s) with scale parameter $s := 1/i$. Because truly active and non-active predictors are generally not known, the comparison of the covariance test statistic along the lasso path to is done to the Exp(1), which is increasingly conservative. This means that as the number of active predictors in the model increases, it is less and less likely to integrate a predictor based on a false

rejection of the null hypothesis that all truly active predictors are already included in the model. Thus, for a fixed value of the covariance test statistic, the p-value obtained under Exp(1) is greater than under an exponential distribution with smaller scaling parameter (Fig. S24c). This leads to the observation of the highly left-skewed p-value distribution (Fig. S24d), which favors the parsimony of the model (Lockhart et al. (2014)). Thus, as expected for lasso-based network inference, only very few predictor genes are required for the prediction of the expression levels of specific response genes, whereas the majority of predictors is shrunken to zero.

2 Supplementary Figures

2.1 Figure S1: Tissue origins of considered CCLE cancer cell lines

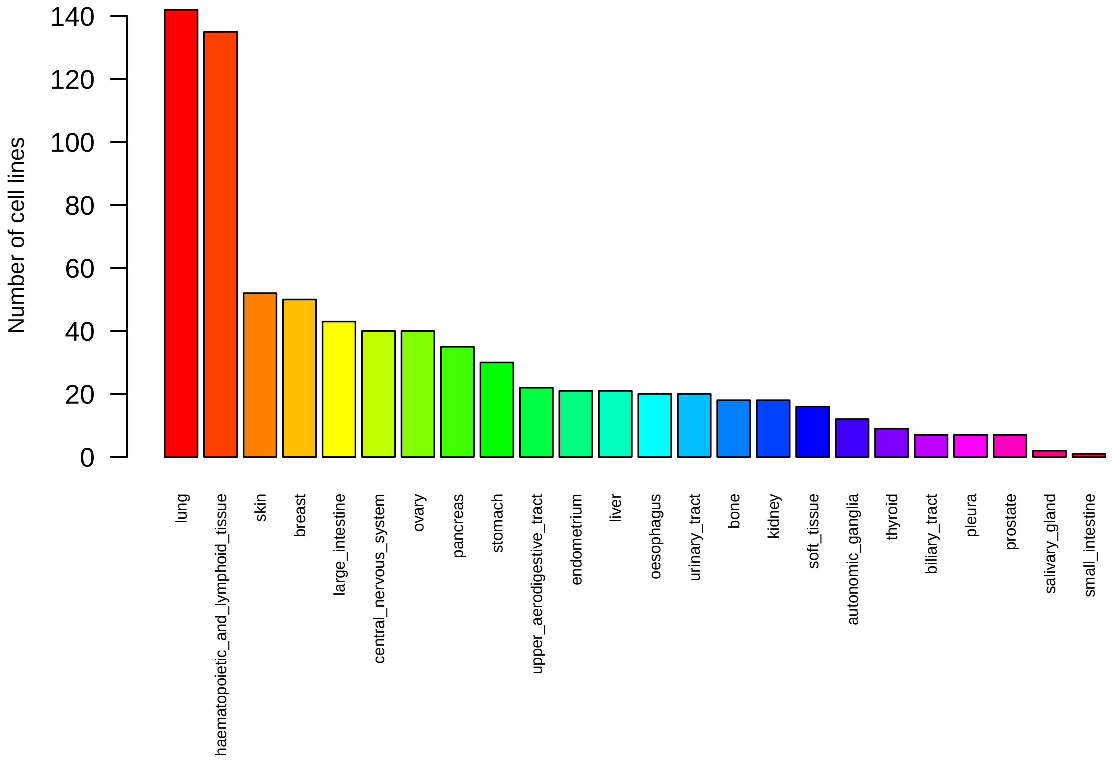

Figure S1: Tissue origins of the 768 CCLE human cancer cell lines that were used to learn CCTN. See Tab. S1 for identifiers of included cell lines.

2.2 Figure S2: CCLE microarray hybridization artifacts

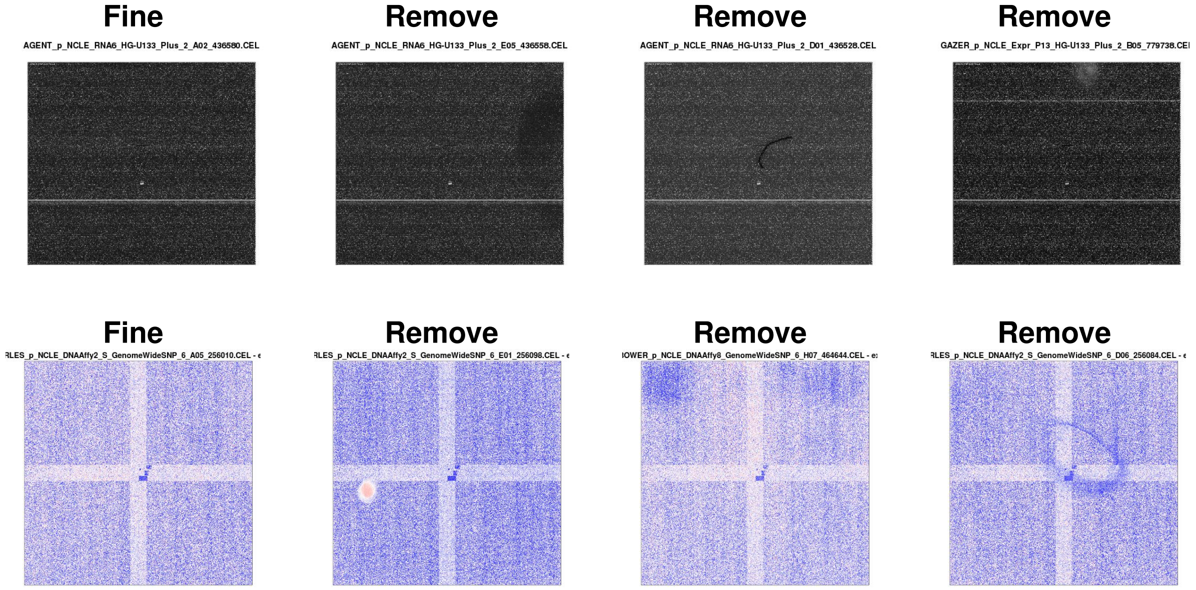

Figure S2: Examples of CCLE microarray hybridization images reconstructed from downloaded CEL files. The top row represents four hybridization images from Affymetrix GeneChip Human Genome U133 Plus 2.0 Arrays that were used to measure gene expression levels. The bottom row represents four hybridization images from Affymetrix Genome-Wide Human SNP 6.0 Arrays that were used to measure copy number variations. We kept microarrays from the CCLE data set with hybridization images highly similar to those labeled as 'Fine' and removed all microarrays that had artifacts similar to those labeled as 'Remove'. In total, 26 of 991 gene expression and 197 of 991 gene copy number arrays had hybridization artifacts. We focused on the remaining 768 CCLE cancer cell lines for which expression and copy number microarrays were both of 'Fine' quality. See Tab. S1 for included cell lines.

2.3 Figure S3: Genome-wide visualization of CCTN

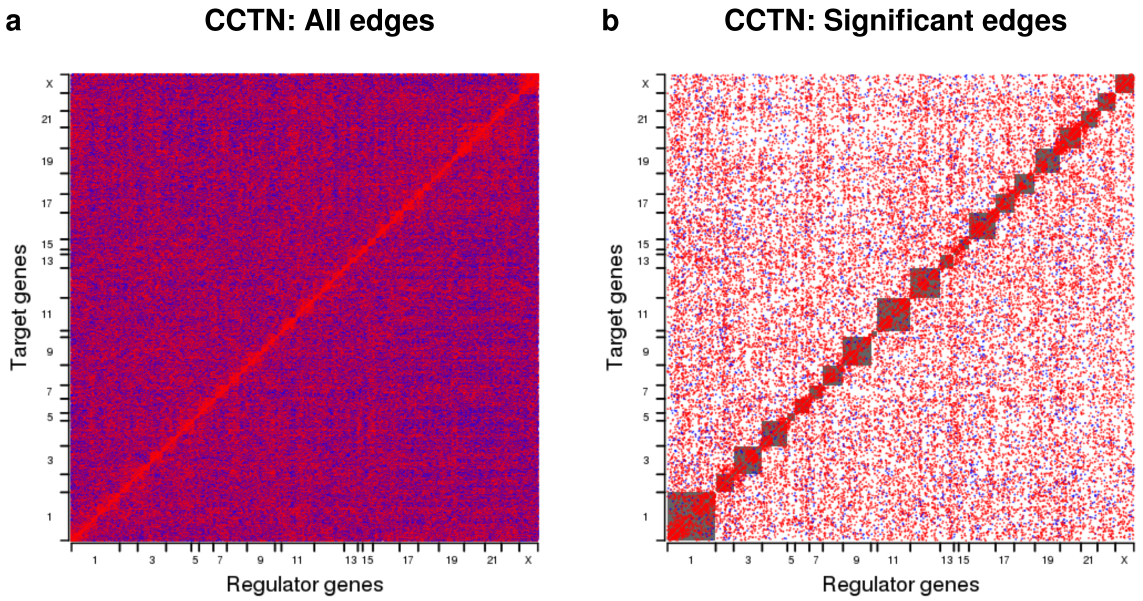

Figure S3: Visualization of CCTN derived from 768 CCLE human cancer cell lines. **a,** CCTN including all regulatory links. **b,** CCTN only including significant regulatory links (p-value < $5 \cdot 10^{5}$) and cleaned up for local regulators 50 genes up- and downstream of each target gene. Edges from regulator to target genes are displayed by colored dots (red: activation, blue: repression). Regulator and target genes are aligned in chromosomal order from 1 to X. The red band around the main diagonals represents local chromosomal activator interactions remaining after the removal of local interactions (50 genes up- and downstream of each target). A similar band can also be observed in a genome-wide regulatory network learned from a large collection of human gene expression profiles by Belcastro et al. (2011). See Tab. S2 for a tab-delimited representation of CCTN in b.

2.4 Figure S4: CCTN predictions for independent TCGA cohorts

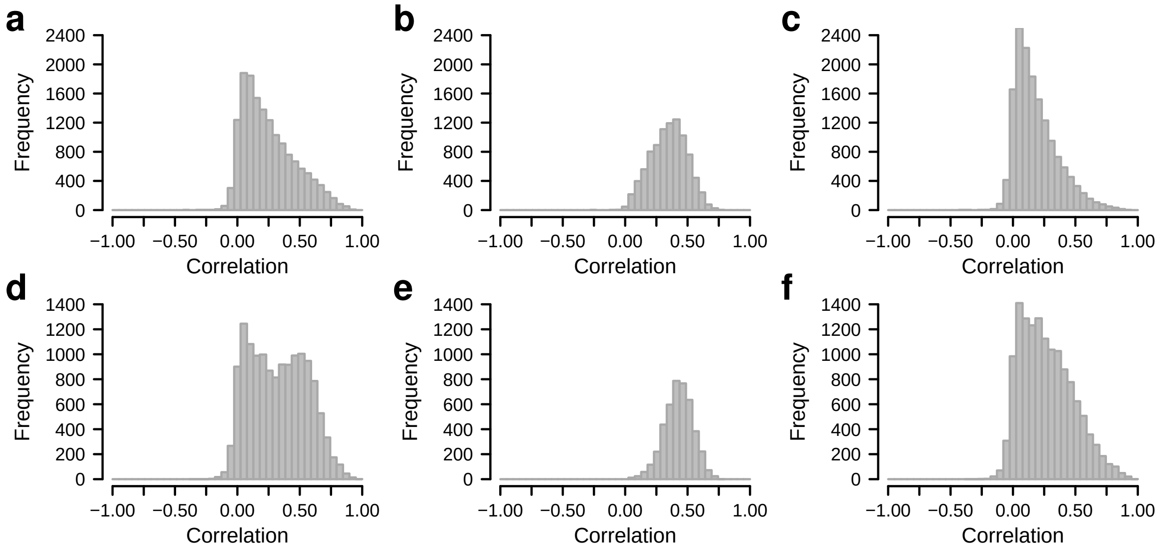

Figure S4: Histograms of median gene-specific correlations between CCTN-predicted and measured expression levels of individual genes summarized for 13 TCGA cohorts. Gene expression and gene copy number data for each of the 4,548 tumor patients were used to predict the patient-specific expression level of each gene using CCTN. **a – c,** CCTN predictions including all links. **d – f,** CCTN predictions only including significant links (p-values $< 5 \cdot 10^{-5}$) and cleaned up for local regulators 50 genes up- and downstream of each target. **a and d,** Predictions including direct copy number and trans-regulatory effects. **b and e,** Predictions only including direct copy number effects. **c and f,** Predictions only including trans-regulatory effects. Generally, the predictions of CCTN focusing on significant predictors was significantly improved compared to CCTN using all predictors. This was confirmed by one-sided Wilcoxon-Mann-Whitney tests evaluating the significance of a shift towards stronger positive correlations for pairs of correlation distributions (**a to d,** p-value $< 6 \cdot 10^{-169}$; **b to e,** p-value $< 6.5 \cdot 10^{-210}$; **c to f,** p-value $< 8 \cdot 10^{-275}$).

2.5 Figure S5: CCTN predictions for individual TCGA cohorts

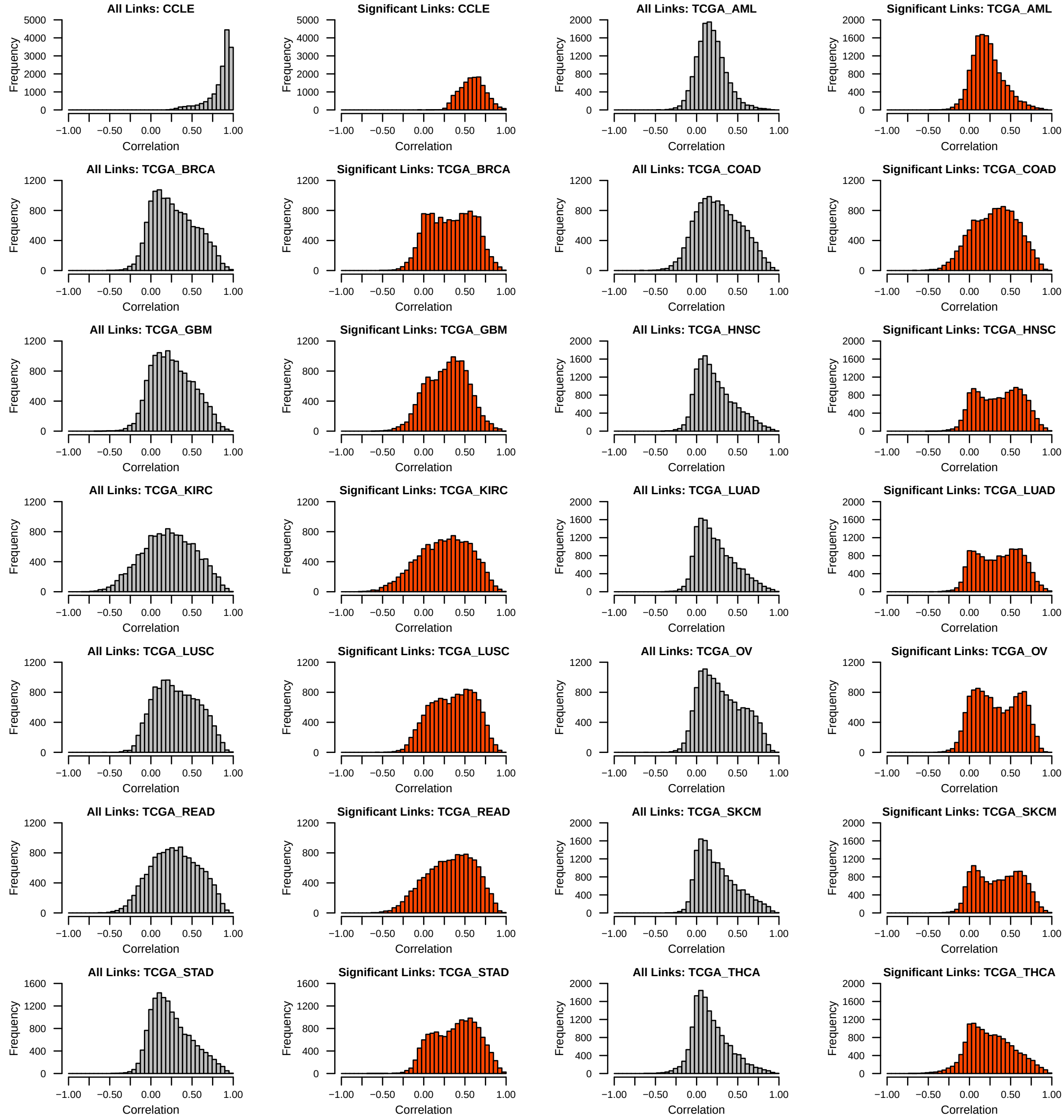

Figure S5: Histograms of gene-specific correlations between CCTN-predicted and measured gene expression levels for CCLE and 13 TCGA cohorts. Gene expression and gene copy number data of each tumor cell line (CCLE) or tumor patient (TCGA) were used to predict the specific expression level of each gene using CCTN. Correlations between predicted and measured expression levels were computed for each gene in a cohort across all samples. Cohort-specific predictions by CCTN including all predictors (All Links) are shown in grey. Corresponding predictions of CCTN only focusing on significant predictors (Significant Links: p-values $< 5 \cdot 10^{-5}$ and cleaned up for potential local regulators 50 genes up- and downstream of each target gene) are shown in orange. For the two CCLE sub-figures, the prediction power of CCTN on the CCLE training data is shown. Considering the TCGA sub-figures, the application of CCTN restricted to significant links substantially improved the predictive power on tumor data (orange vs. grey distributions) for all considered tumor types. The restriction to significant links often resulted in characteristic bi-modal distributions with a second strong peak in the range of positive correlations.

2.6 Figure S6: Robustness of CCTN predictions for varying p-value cutoffs

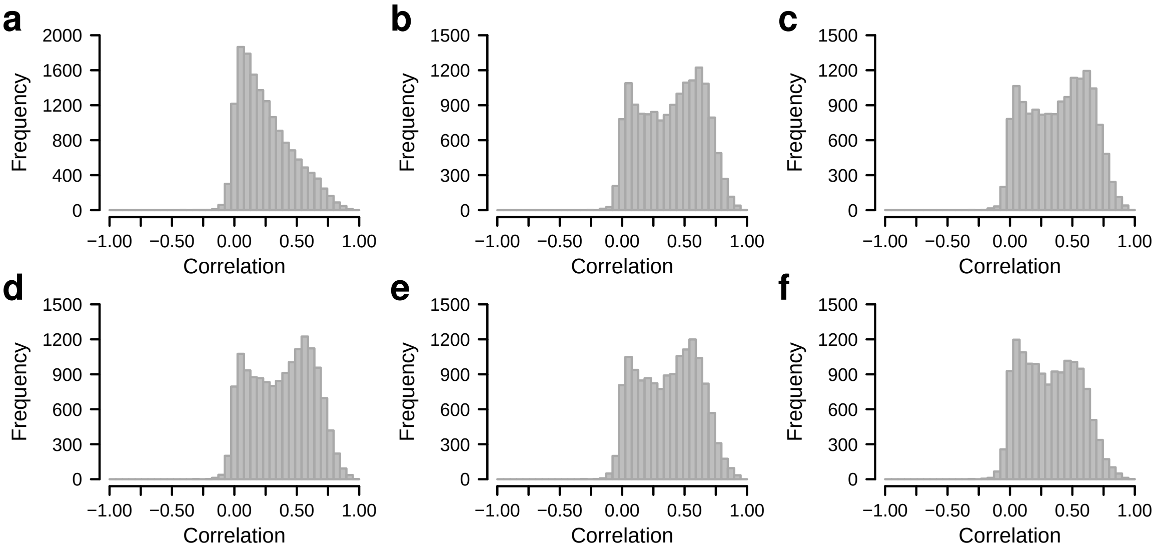

Figure S6: Histograms of median gene-specific correlations between predicted and measured gene expression levels of individual genes in 13 TCGA cancer cohorts. Gene expression and gene copy number data for each of the 4,548 tumor patients were used to predict the patient-specific expression level of each gene using CCTN. **a,** CCTN predictions including all selected predictors, **b - e,** CCTN predictions including only significant predictors with respect to decreasing p-value cutoffs: **b,** p-value < 0.1; **c,** p-value < 0.05; **d,** p-value < 0.01, and **e,** p-value < $5 \cdot 10^{-5}$. **f,** CCTN predictions as considered in the main manuscript and the appendix unless stated otherwise. Only predictors with p-values < $5 \cdot 10^{-5}$ were kept and potential local regulator genes 50 genes up- and downstream of each target gene were removed to avoid the integration of spurious regulators that may just reflect the copy number state of a target gene instead of true regulatory interactions.

2.7 Figure S7: Comparison of CCTN to tumor type-specific networks

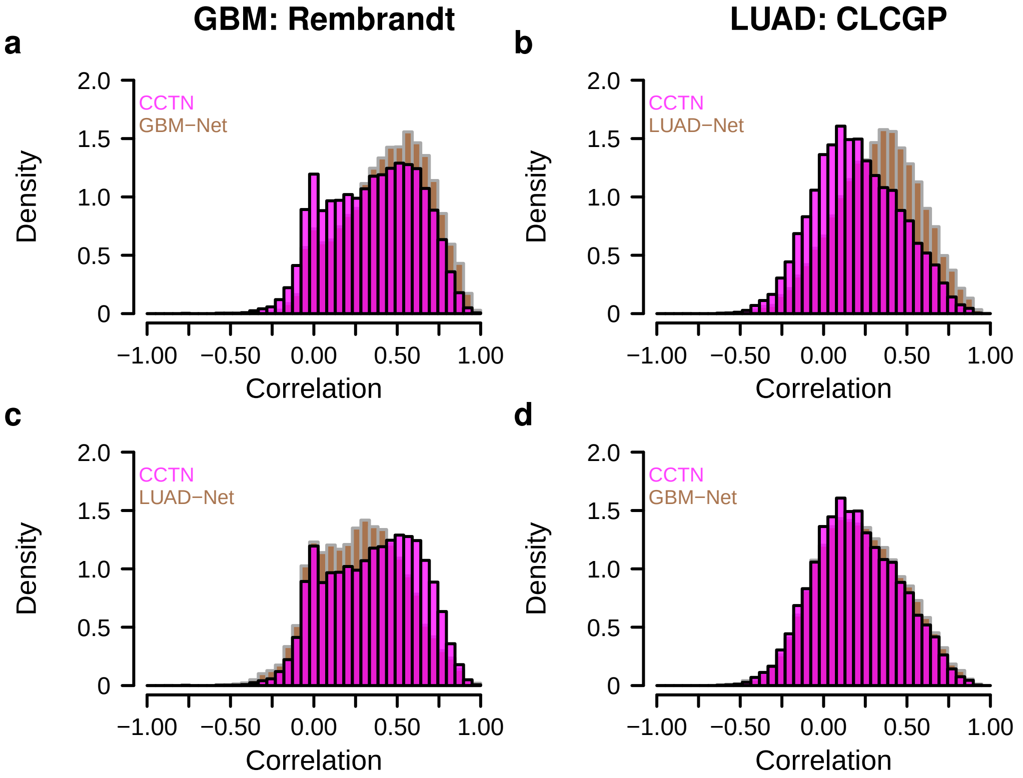

Figure S7: Comparison of CCTN predictions to predictions of tumor type-specific network models. CCTN was trained as described in the main manuscript using gene copy number and gene expression data of 768 cancer cell lines. We further trained two tumor type-specific networks using gene copy number and gene expression data of the TCGA glioblastoma (GBM) and TCGA lung adenocarcinoma (LUAD) cohorts to compare the prediction quality of CCTN to tumor type-specific networks. We evaluated the prediction quality of CCTN, GBM-Net and LUAD-Net on independent glioblastoma data from Rembrandt (Madhavan et al. (2009)) and lung adenocarcinoma from CLCGP (CLCGP 2013) by computing the correlations between network-based predicted and experimentally measured expression levels for each gene as described in the main manuscript. **a,** CCTN and GBM-Net predictions of gene expression levels for glioblastoma patients from Rembrandt. Overall range of reached correlations of both network models is quite similar. The tumor type-specific GBM-Net reaches a higher proportion of correlations greater than 0.25 compared to CCTN. **b,** CCTN and LUAD-Net predictions of gene expression levels for lung adenocarcinoma patients from CLCGP. Similar like in subpanel **a**, the tumor type-specific LUAD-Net reaches a higher proportion of correlations greater than 0.25 compared to CCTN. **c,** CCTN and LUAD-Net predictions of gene expression levels for glioblastoma patients from Rembrandt. CCTN reaches a higher proportion of correlations greater than 0.5 compared to LUAD-Net indicating that CCTN tends to better generalize to glioblastoma data than LUAD-Net. **d,** CCTN and GBM-Net predictions of gene expression levels for lung adenocarcinoma patients from CLCGP. Both network models reach nearly the same prediction quality. In summary, tumor type-specific network models tend to reach moderately better predictions for their specific tumor domain than CCTN (**a** and **b**), but the generalization of these tumor type-specific network models to other tumor types is weaker than for CCTN (**c**) or in the range of CCTN (**d**).

2.8 Figure S8: Summary of LINCS-based validation of CCTN impact values

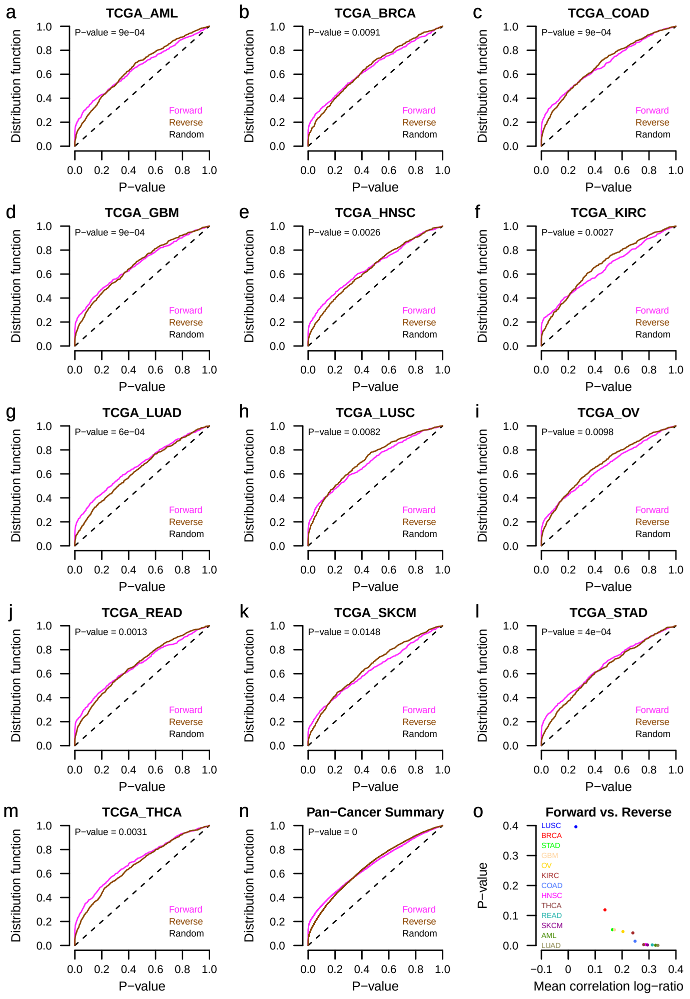

Figure S8: LINCS-based validation of CCTN-based network propagation. **a – m,** Cumulative p-value distributions correlating experimentally measured and computationally predicted single-gene perturbations. Forward: P-values of correlations between computed impacts flowing from a perturbed regulator to its targets and experimentally measured impacts. Reverse: P-values of correlations between computed impacts flowing in the reverse direction from responding targets to their perturbed regulator and experimentally measured forward impacts. Random: Baseline for non-significant enrichment of small p-values. Individual results are shown for 13 TCGA cohorts utilizing the corresponding CCTN-derived impact matrix. If CCTN would only represent a coexpression network, then the forward and the backward model would reach identical predictive power for the prediction of experimentally measured genes that respond to single-gene perturbations, but if the forward model is able to explain single-gene perturbations better than the reverse model this indicates that CCTN is at least in part enriched for potential true causal relationships allowing to predict the directionality of effects. All subpanels show that the forward model better predicts single-gene perturbations than the corresponding reverse model (enrichment of small p-values for forward compared to revers: p-value < 0.015 for all cohorts, one-sided Kolmogorov-Smirnov test comparing the forward model (pink) to the reverse model (brown)). Thus, this indicates that CCTN is enriched for true causal relationships. **n,** Pan-cancer summary pooling the p-values from all 13 TCGA cohorts. **o,** Pairwise comparison of forward and reverse impacts for each TCGA cohort. Correlations between computationally predicted and experimentally measured impacts were computed for each regulator under the forward and the reverse model. Log-ratios of forward versus reverse correlations were computed for each regulator and plotted on the x-axis as average across all regulators for each TCGA cohort. The y-axis shows the corresponding p-values from one-sided paired t-tests testing if the average correlation for the forward model is significantly larger than the correlation for the reverse model. The correlations of the forward model are on average significantly greater than the corresponding correlations of the reverse model for 11 of 13 TCGA cohorts (p-value < 0.1). This again supports that CCTN can correctly predict the directionality of effects.

2.9 Figure S9: RF-based prediction of patient survival for TCGA cohorts

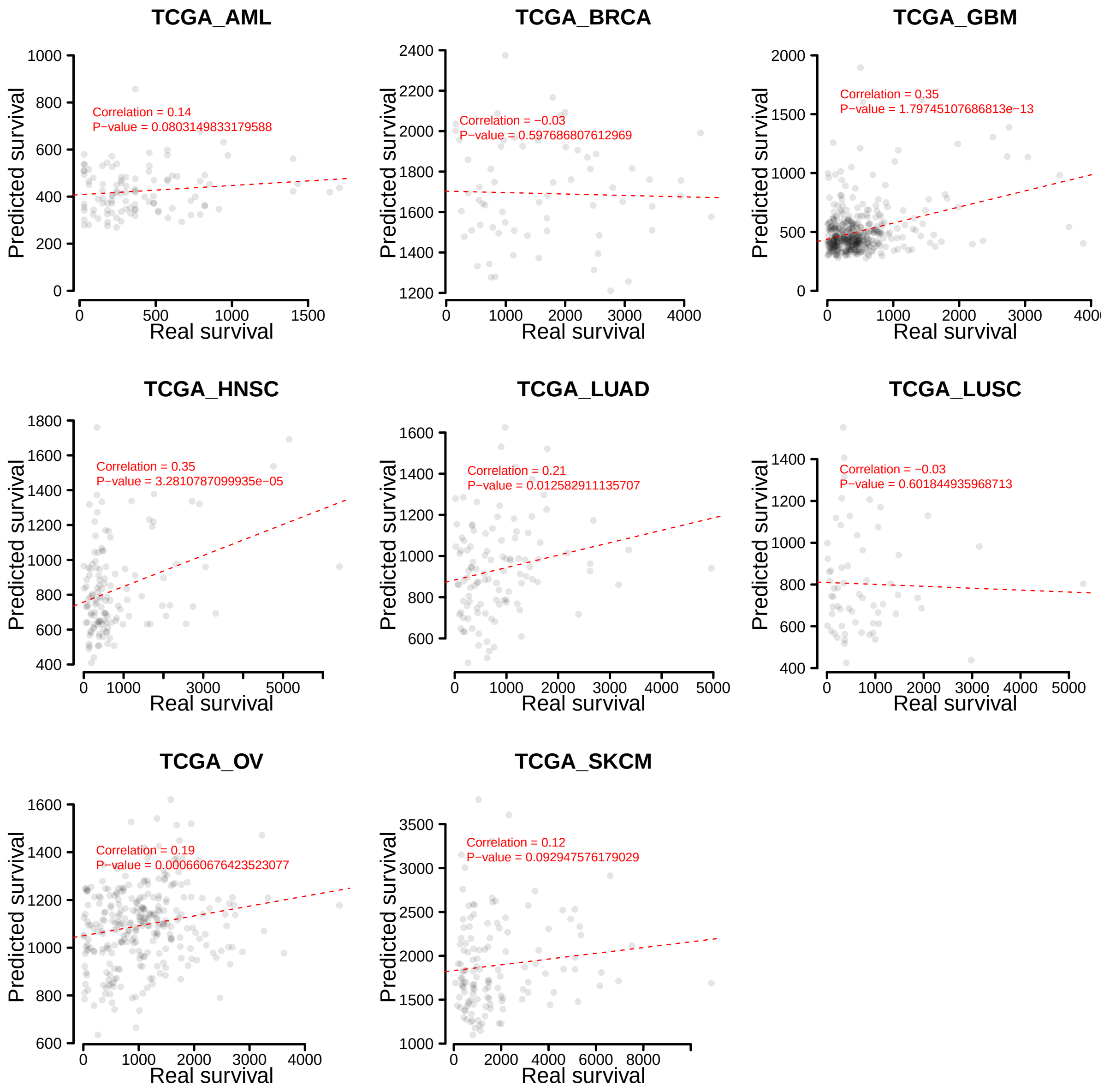

Figure S9: Performance of patient survival prediction for individual TCGA cancer cohorts containing more than 20 patients with survival information. A Random Forest (RF) was learned on each cohort based on patient-specific gene expression data and survival information. Resulting RF-based survival prediction is shown for out-of-bag data (patient-specific expression profiles that were not utilized during the construction of individual trees of the RF). Based on these results, BRCA and LUSC were excluded from the subsequent analysis and only the cohorts with more than 100 patients (AML, GBM, HNSC, LUAD, OV, and SKCM) were kept.

2.10 Figure S10: Comparison of RF to RSF without and with censoring

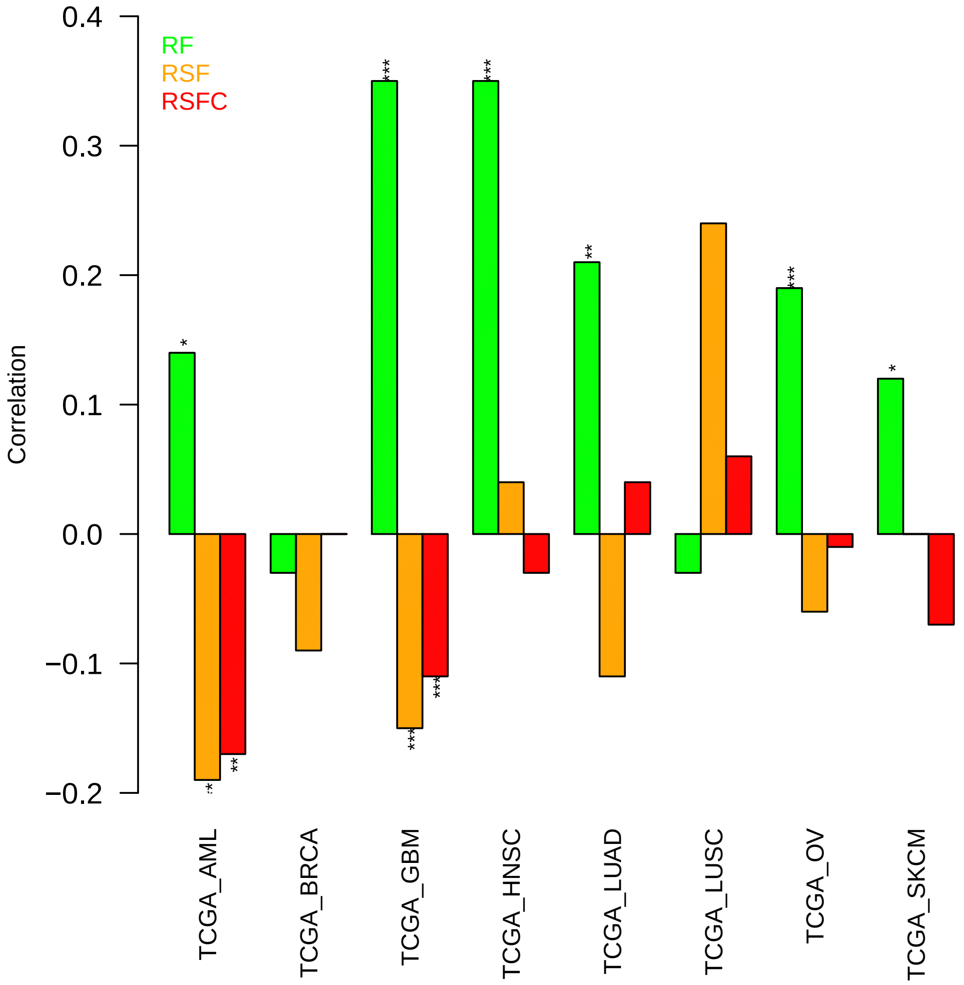

Figure S9: Survival prediction on held out patient samples comparing our random forest approach (RF) against random survival forest without (RSF) and with censoring (RSFC). The RF-approach directly predicted survival for each patient, whereas RSF and RSFC predict a mortality value for each patient. RF and RSF were trained on each TCGA cohort on the same set of patients that were already dead. RSFC additionally utilized all patients that were alive (censoring). Survival prediction quality was evaluated by correlating the predicted survival or mortality with real patient survival. Note that one would expect negative correlations between mortality and real survival for a predictive RSF and RSFC on held out samples, because a high mortality indicates a low patient survival. Significant correlations are represented by '*' (p-value < 0.1), '**' (p-value < 0.05) and '***' (p-value < 0.01). Overall, RF tended to reach the best prediction of patient survival from gene expression data except for AML.

2.11 Figure S11: RF-based prediction of Rembrandt GBM patient survival

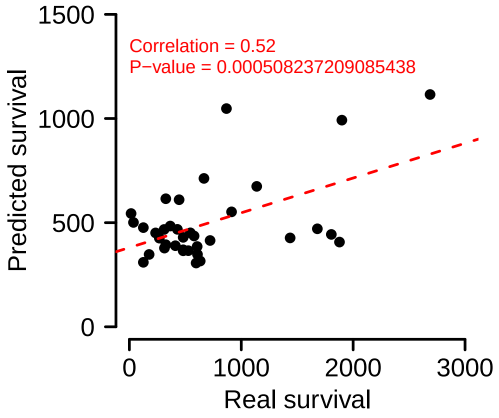

Figure S11: Evaluation of the survival prediction quality of the TCGA GBM Random Forest (RF) for independent GBM patients from Rembrandt (Madhavan et al. (2009)). Data were filtered and pre-processed as described in Seifert et al. (2014). Gene expression data and survival information of 36 GBM patients were used to test the RF obtained from the TCGA GBM cohort. The obtained significant correlation between RF-predicted and real patient survival clearly indicates that the RF has learned GBM relevant survival signature genes.

2.12 Figure S12: Selection of survival predictor genes for TCGA cohorts

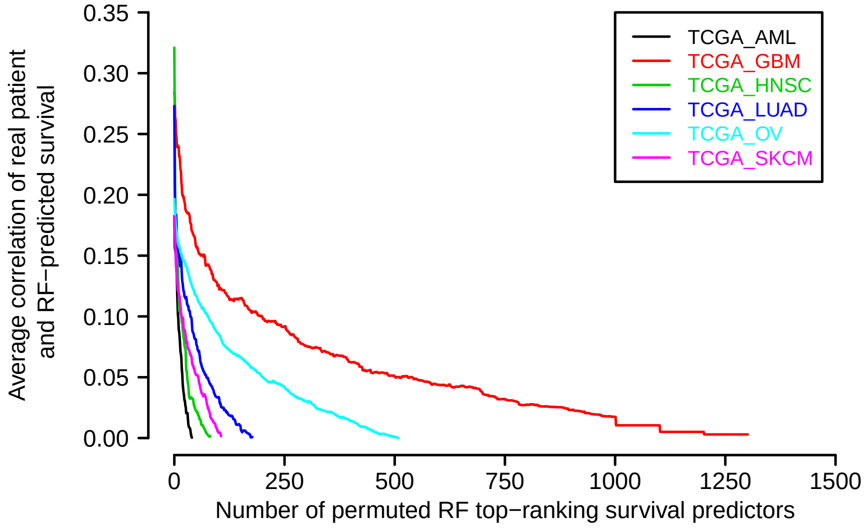

Figure S12: Average correlation profiles between RF-predicted and real patient survival for successively increasing numbers of permuted top-ranking survival predictors for 1,000 permutation runs. Individual TCGA cohort-specific correlation profiles dye out towards zero enabling to select the most relevant genes associated with patient survival. We considered a stringent (all predictor genes above a correlation cutoff of 0.1) and a less stringent correlation cutoff (all predictor genes above a correlation cutoff of 0.05) to derive TCGA cohort-specific survival signature genes. Results for survival signature genes obtained based on the stringent cutoff are described in the main manuscript. Similar results obtained for the less stringent cutoff are additionally shown in the appendix (Fig. S13 and Fig. S17). See Tab. S6 for TCGA cohort-specific survival signature genes.

2.13 Figure S13: TCGA cohort-specific survival signatures

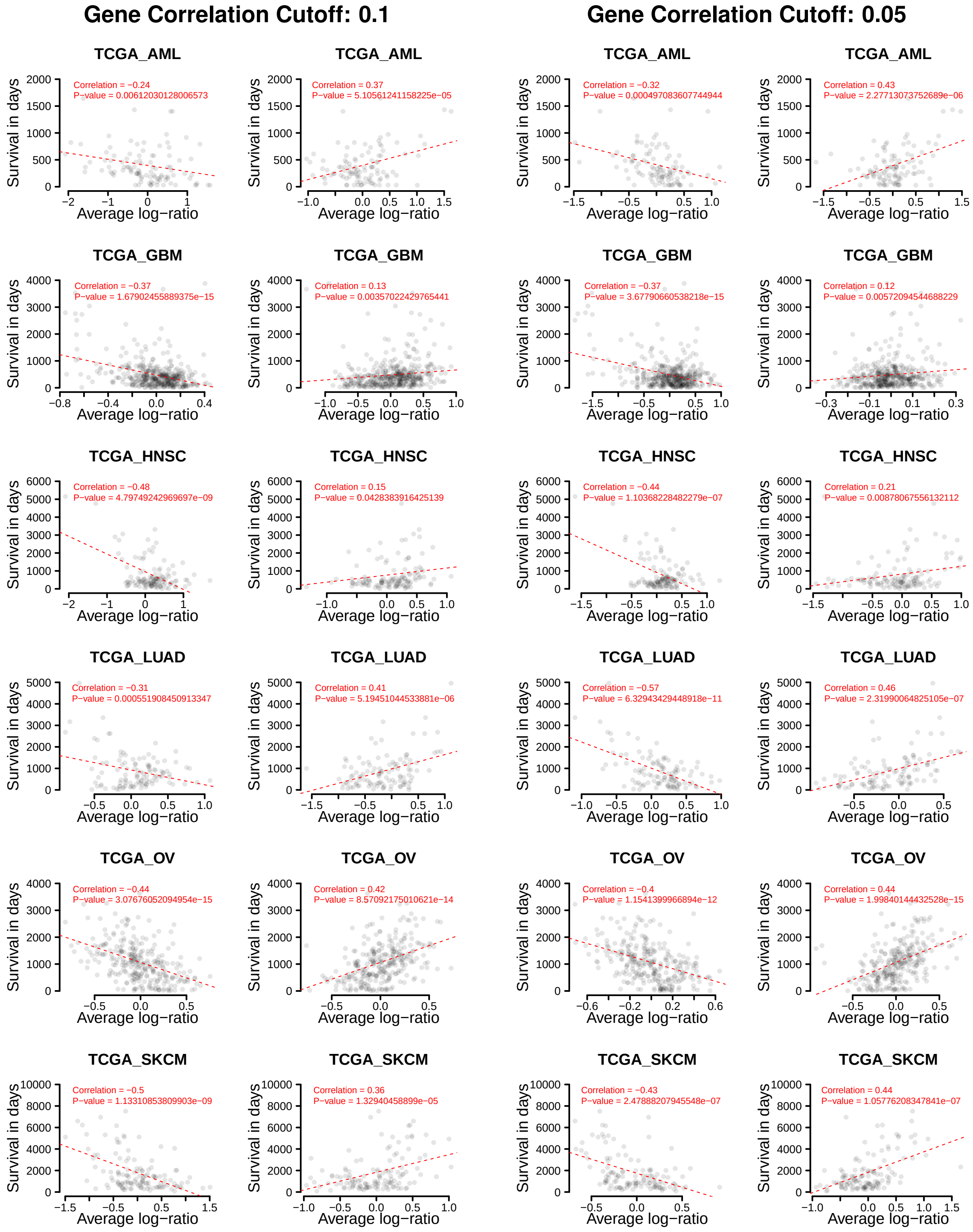

Figure S13: TCGA cohort-specific survival signatures obtained based on survival signature genes identified with our RF approach. Underlying cohort-specific survival signature genes were selected based on cohort-specific correlation profiles in Fig. S12 quantifying the importance of individual predictor genes. The two left columns represent results for survival signature genes above a stringent correlation cutoff of 0.1 in Fig. S12. The two right columns represent corresponding results for survival signature genes above a less stringent correlation cutoff of 0.05. For each TCGA cohort, we grouped the selected survival signature genes into two clusters using hierarchical clustering. We always obtained a cluster of genes that were positively correlated with survival and a cluster of genes that were negatively correlated with survival. Finally, we computed for each tumor patient the corresponding average expression levels of the survival signatures for the two corresponding TCGA cohort-specific survival gene clusters and plotted the obtained average survival signature gene expression levels of each gene cluster against the corresponding patient survival information (grey dots). Correlation tests confirmed that the average expression levels of survival signature genes of individual tumor patients is significantly associated with their survival.

2.14 Figure S14: Validation of entire pipeline on Rembrandt GBM cohort

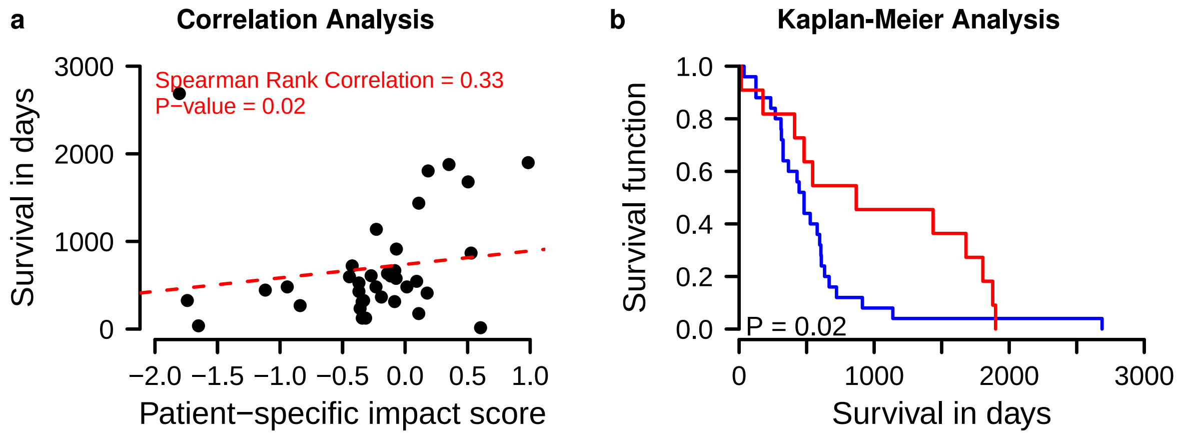

Figure S14: Integrated validation of the entire impact computation pipeline from CCTN-based network prediction to CCTN-based impact computation utilizing the independent Rembrandt GBM cohort. **a,** Gene expression and corresponding gene copy number profiles of GBM patients from the Rembrandt cohort (Madhavan et al. (2009)) were filtered and pre-processed in Seifert et al. (2014). We first demonstrated that the survival of Rembrandt GBM patients was predictable using the Random Forest (RF) learned from gene expression profiles and survival information of GBM patients from the TCGA cohort (Fig. S11). We then determined all GBM survival signature genes from the TCGA GBM RF that were above a correlation cutoff of 0.05 (Fig. S12, Tab. S6). We now computed for each of the 36 Rembrandt GBM patients a patient-specific impact matrix utilizing our network propagation algorithm with respect to initial computations based on CCTN using the patient-specific gene expression and gene copy number profiles (Text S1: Patient-specific relative impact scores). We next determined for each Rembrandt GBM patient all deleted and amplified genes (CNA cutoff: absolute gene-specific aCGH log-ratio $\geq$ 0.75) and determined their individual impacts on the GBM survival signature genes derived from the TCGA GBM RF. Each obtained impact of a specific gene deletion (or amplification) on a specific survival signature gene quantifies if this deletion (or amplification) has a repressive (impact < 0), no (impact = 0), or an activating (impact > 0) impact on the expression of the survival signature gene. Next, we know for all TCGA GBM RF survival signature genes if their expression is positively or negatively correlated with TCGA GBM patient survival. Considering the Rembrandt GBM patients, we now multiplied the individual impacts of each patient-specific deleted/amplified gene with the corresponding signs of the correlations of the survival signature genes that were affected by this gene CNA. Note that each of these impact scores now quantifies if the repressive or activating impact of the underlying gene CNA has a positive or negative contribution on patient survival. Finally, we computed the sum of all impact scores obtained for all patient-specific gene deletions and amplifications resulting in a patient-specific impact score. Based on the overall definition of this patient-specific impact score, negative scores should be associated with shorter survival than positive impact scores. To test this, we plotted the patient-specific impact scores (x-axis) against the corresponding individual patient survival (y-axis). The patient-specific impact scores are significantly positively correlated with survival (Spearman rank correlation test: rho = 0.33, p-value = 0.024). In addition, a t-test confirmed that Rembrandt GBM patients with positive patient-specific impact scores tended to survive significantly longer than patients with negative impact scores (p-value < 0.04). **b,** Corresponding Kaplan-Meier curves for Rembrandt GBMs representing patients with negative (blue, patients with short survival) and positive (red, patients with longer survival) impact scores. The red curve is significantly above the blue curve (p-value = 0.02) clearly indicating that the obtained patient-specific impact scores can distinguish short from long survival.

2.15 Figure S15: Selection of gene CNAs with high impact on survival

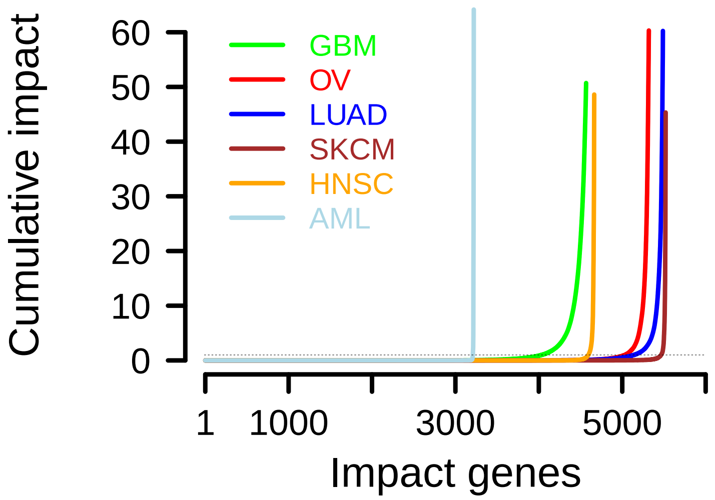

Figure S15: Selection of gene copy number alterations (deletions or amplifications) with high impact on TCGA cohort-specific survival signature genes. All genes with at least one CNA in a TCGA cohort were determined (CNA cutoff: absolute gene-specific aCGH log-ratio ≥ 0.75). Average impacts of each of these genes on corresponding cohort-specific survival signature genes (genes with correlation ≥ 0.1 in Fig. S12) were computed based on the cohort-specific impact matrix obtained from our network propagation algorithm. The majority of cohort-specific gene CNAs had only very little impact (values very close to zero) on survival signature genes. In addition, there were also always gene CNAs in each cohort that had high impact on the survival signature genes. We therefore ranked the gene CNAs in each cohort from low to high impacts and computed the cumulative sum of impacts. We then plotted the cumulative impact against the number of involved CNA genes enabling to distinguish between low impact genes (cumulative impact close to zero) and high impact genes (cumulative impact clearly greater than zero). For each TCGA cohort, we selected all genes above a cumulative impact cutoff of one (black dashed line) as corresponding high survival impact genes. The resulting genes are considered in the analysis that is part of the main manuscript. See Tab. S7 for individual impact scores of TCGA cohort-specific genes affected by CNAs. We further confirmed that the selected survival impact genes above the black dashed line had impact values that were highly significant in comparison to corresponding gene-specific impact scores obtained for each tumor cohort under consideration of ten random networks of the same complexity as CCTN (degree-preserving network permutations). The obtained q-values for all cohort-specific selected CNA-impact genes were less than 0.006 (ranged from 0.0048 for GBM to 0.0058 for HNSC). See methods in the main manuscript for details.

2.16 Figure S16: Genomic locations of rare and frequent survival-impact genes

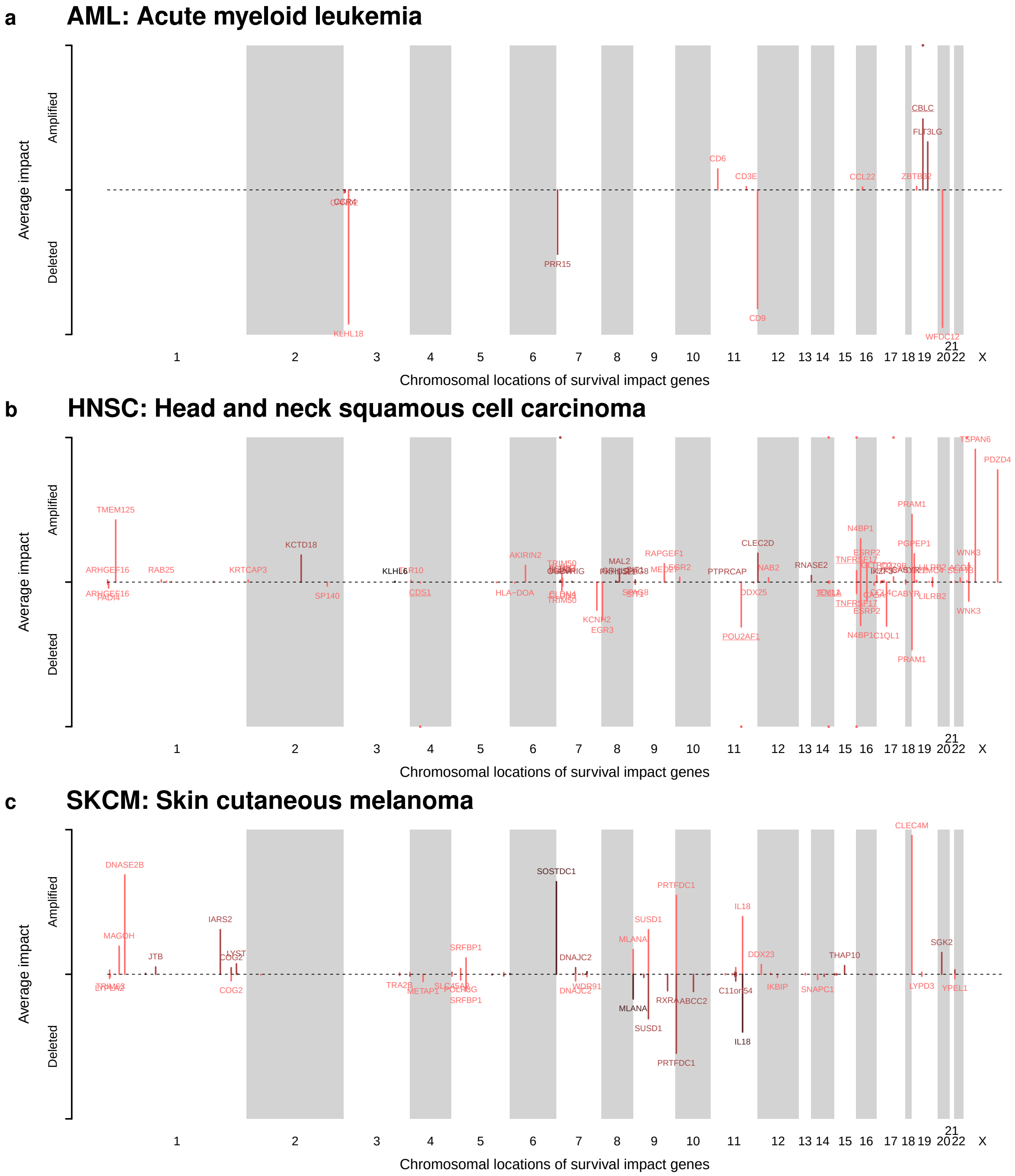

Figure S16: Chromosomal locations of gene CNAs with high-impact on survival signatures for TCGA cohorts AML, HNSC and SKCM. Deletions and amplifications of individual genes were determined for each TCGA cohort (CNA cutoff: absolute gene-specific aCGH log-ratio ≥ 0.75) and further filtered for gene CNAs with high impact on survival signature genes (High-impact cutoff: all CNAs with cumulative impact cutoff > 1 in Fig. S15). **a,** AML-specific gene CNAs with high-impact on AML survival signature genes. **b,** HNSC-specific gene CNAs with high-impact on HNSC survival signature genes. **c,** SKCM-specific gene CNAs with high-impact on SKCM survival signature genes. Genes are colored based on their observed mutation frequency (light red: rare, black: frequent) and displayed by peaks with heights according to their impact on survival signature genes. Selected gene names are displayed depending on their height of impact. Gene names of known cancer genes are additionally underlined. Colored dots at both the outer sides of the y-axis additionally highlight the position of identified high-impact genes previously reported as cancer genes.

2.17 Figure S17: Patient-specific survival risk curves

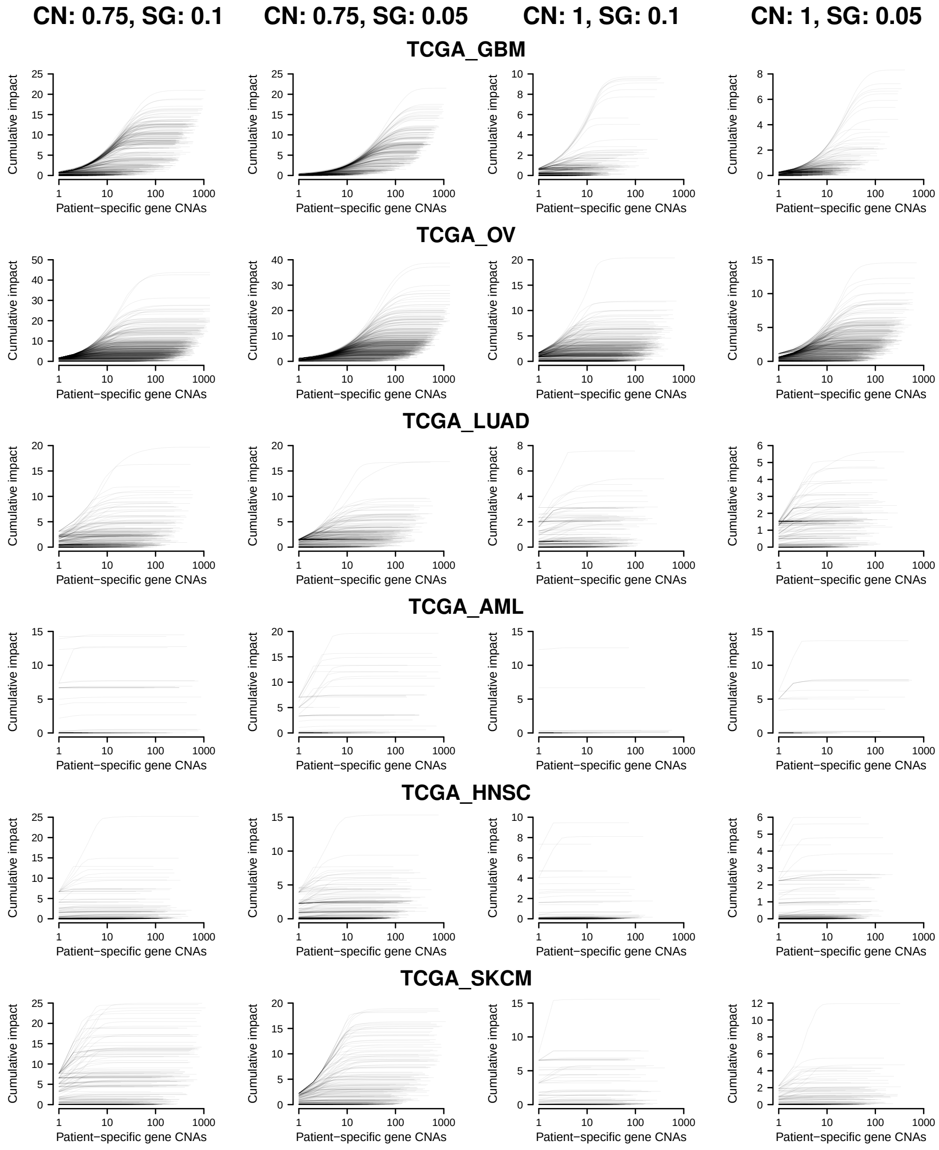

Figure S17: Patient-specific survival risk curves for individual TCGA cohorts. We determined genes affected by deletions and amplifications for each tumor patient and used the cohort-specific survival impact matrix obtained from our network propagation algorithm to determine the impact of each patient-specific gene CNA on the cohort-specific survival signature. We next ranked all patient-specific gene CNAs from high to low impact and computed corresponding cumulative impacts. We finally plotted for each patient its corresponding cumulative impact curve quantifying the impacts of individual gene CNAs on survival signature genes. **CN: 0.75, SG: 0.1,** Shows patient-specific survival risk curves for patient-specific gene CNAs with an absolute gene copy number log-ratio $\geq$ 0.75 and corresponding cohort-specific survival signature genes above a correlation cutoff of 0.1 in Fig. S12. **CN: 0.75, SG: 0.05,** Corresponding patient-specific survival risk curves for a less stringent selection of survival signature genes (genes with a correlation above 0.05 in Fig. S12). Thus, the same patient-specific gene CNAs as in CN: 0.75, SG: 0.1 are acting on an increased number of signature genes. **CN: 1, SG: 0.1,** Shows patient-specific survival risk curves for patient-specific gene CNAs with an absolute gene copy number log-ratio $\geq$ 1 and corresponding cohort-specific survival signature genes above a correlation cutoff of 0.1 in Fig. S12. Thus, a reduced number of patient-specific gene CNAs as in CN: 0.75, SG: 0.1 are acting on the same number of signature genes. **CN: 1, SG: 0.05,** Corresponding patient-specific survival risk curves for a less stringent selection of survival signature genes (genes with a correlation above 0.05 in Fig. S12). Generally, the overall cohort-specific trends are highly similar for the different cutoffs.

2.18 Figure S18: General characterization of gene CNAs impacting on survival

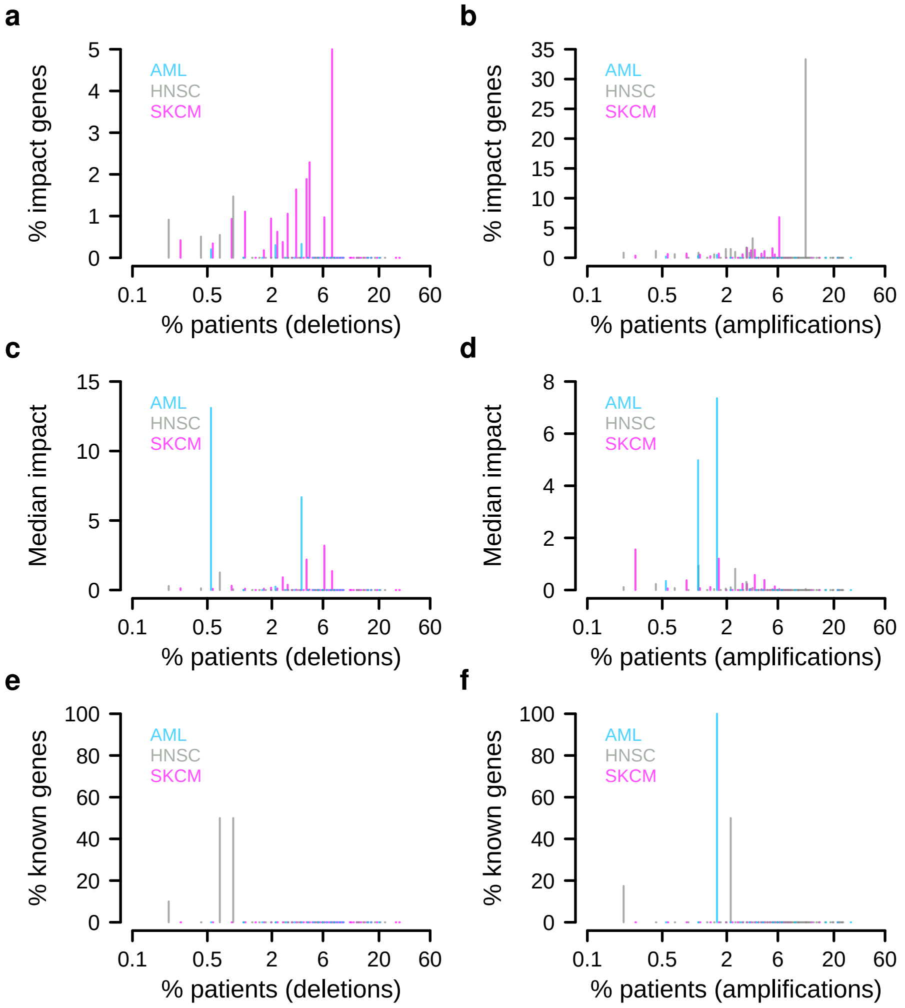

Figure S18: General characterization of cohort-specific gene CNAs with high impact on survival signature genes. Deletions and amplifications of individual genes were determined for each TCGA cohort (CNA cutoff: absolute gene-specific aCGH log-ratio ≥ 0.75) and further filtered for gene CNAs with high impact on survival signature genes (High-impact cutoff: all CNAs with cumulative impact cutoff > 1 in Fig. S15). All gene deletions and amplifications in each TCGA cohort (AML, HNSC, SKCM) were binned separately based on their frequency. **a - b,** Percentage of genes in each bin belonging to the cohort-specific genes with strongest impact (cumulative impact cutoff > 1 in Fig. S15) on survival signature genes. **c - d,** Median impact of strongest impact genes in each frequency bin. The median impact quantifies the contribution of all high impact gene CNAs in a bin to the variation of the expression levels of all cohort-specific survival signature genes. Average percentages of explained variance of survival signature expression computed for all high impact gene CNAs were used to determine the median impact per bin. **e - f,** Proportion of known cancer genes among the strongest impact genes in each bin. Corresponding results for GBM, OV and LUAD are shown in Fig. 5 of the main manuscript.

2.19 Figure S19: Impact of rare and frequent gene CNAs on survival

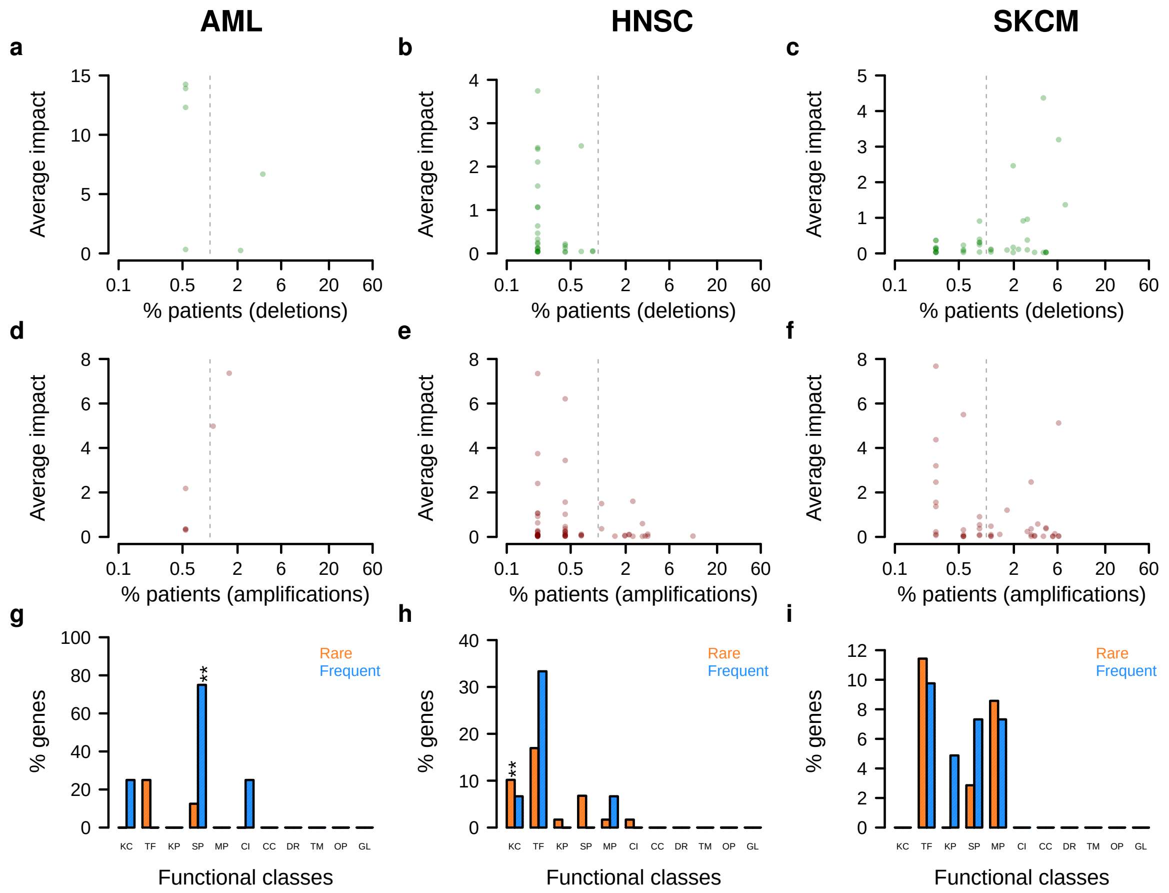

Figure S19: Individual impact of rare and frequent gene CNAs on TCGA cohort-specific survival signatures for AML, HNSC and SKCM. Deletions and amplifications of individual genes were determined for each TCGA cohort (CNA cutoff: absolute gene-specific aCGH log-ratio $\geq$ 0.75) and further filtered for gene CNAs with high impact on survival signature genes (High-impact cutoff: all CNAs with cumulative impact cutoff > 1 in Fig. S15). **a - f,** Average impact of gene deletions (a – c) and amplifications (d – f) on the expression of cohort-specific survival signature genes. The vertical grey dashed line at 1% of patients defines the cutoff used to separate rare from frequent CNAs. **g – i,** Corresponding joint functional annotation of deleted and amplified impact genes. Genes were classified as rarely (CNA frequency< 1%) or frequently (CNA frequency >= 1%) mutated. Proportion of genes in selected functional classes are shown: KC: known cancer genes, TF: transcription factors, KP: kinases and phosphatases, SP: signaling pathways, MP: metabolic pathways, CI: cell-cell interaction, CC: cell cycle, DR: DNA replication, TM: telomere maintenance, OP: oxidative phosphorylation, GL: glycolysis. Significant enrichment of an individual category is represented by '**' (p-value < 0.01). Corresponding results for GBM, OV and LUAD are shown in Fig. 6 of the main manuscript. Note that the height of the bars doses not necessarily correlate with significance due to different sizes of functional classes.

2.20 Figure S20: Identification of survival-associated gene CNAs using t-tests

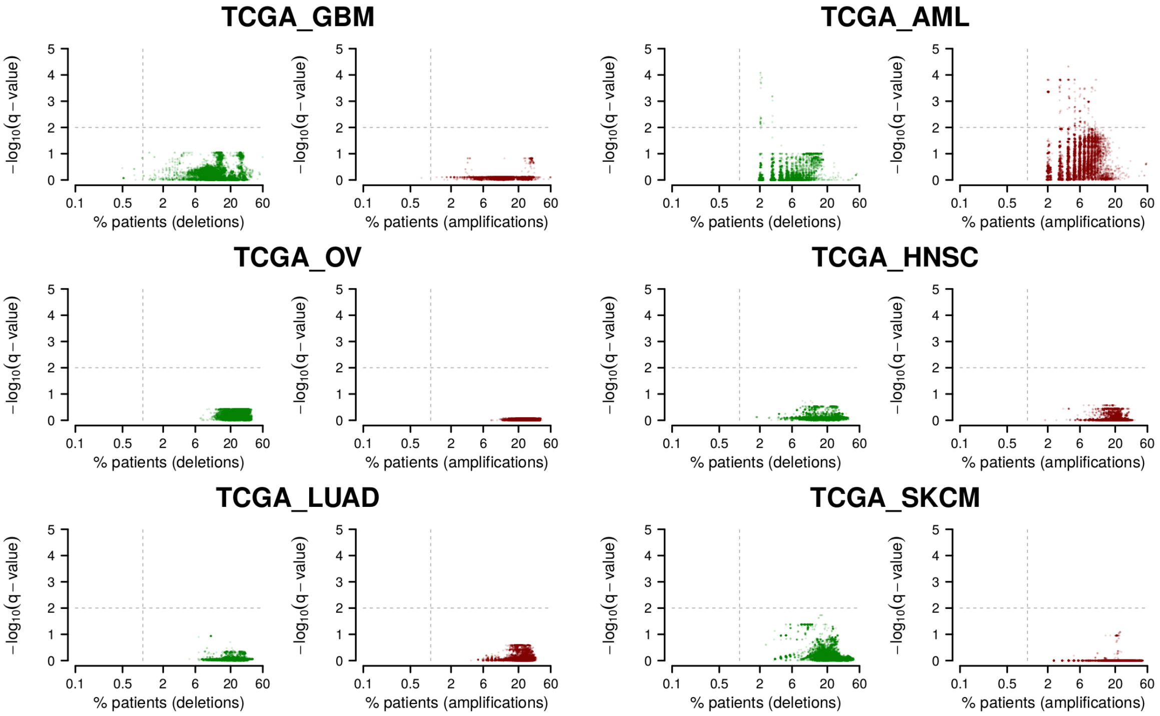

Figure S20: Identification of survival-associated gene CNAs for six TCGA cohorts with sufficient patients using t-tests. For each cohort, each gene that was affected by a CNA in at least two patients was considered as a candidate gene CNA that may influence patient survival. Differences in survival were quantified by a t-test for each candidate gene comparing the survival of patients that had this specific gene CNA to patients that did not show a mutation of this gene. The resulting p-values of all cohort-specific tests were further corrected for multiple testing by computing corresponding q-values. The subpanels highlight the results that were obtained for each individual cohort considering gene deletions (dark green) and amplifications (dark red). The CNA rates of genes among patients with available survival information are displayed in log-scale. The corresponding q-values are shown in negative log-scale. Except for AML, no survival-associated gene CNAs with q-values below 0.01 (grey dashed horizontal line) were detectable. Further, for non of the cohorts any rare gene CNA occurring in less than 1% of patients could be identified (grey dashed vertical line).

2.21 Figure S21: Distance of survival impact genes from genomic features

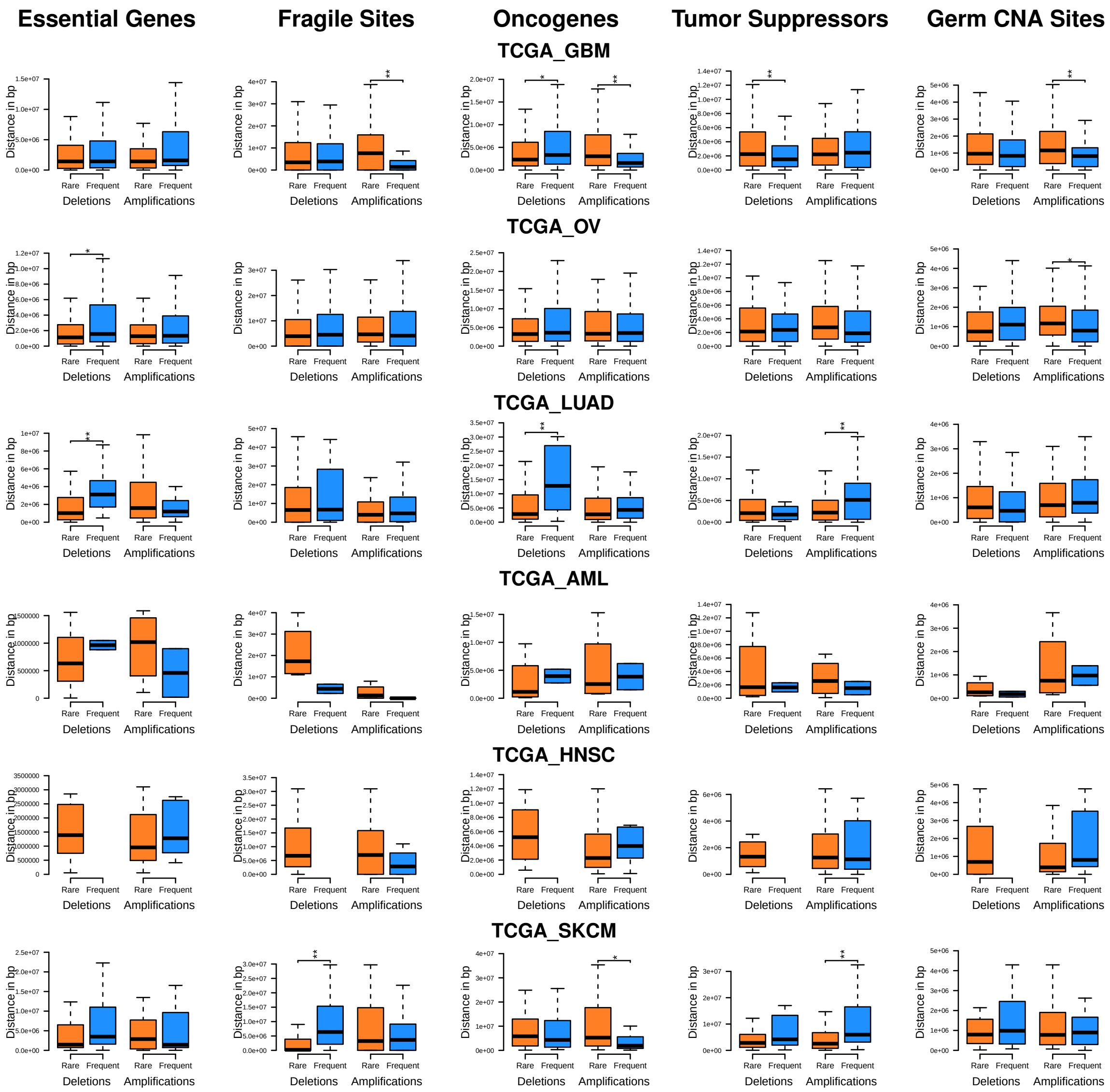

Figure S21: Distances of rare and frequent high-survival-impact gene CNAs from genomic features. Deletions and amplifications of individual genes were determined for each TCGA cohort (CNA cutoff: absolute gene-specific aCGH log-ratio ≥ 0.75) and further filtered for gene CNAs with high impact on survival signature genes (High-impact cutoff: all CNAs with cumulative impact cutoff > 1 in Fig. S15). The resulting cohort-specific high-impact gene CNAs were further separated into rarely (CNA frequency< 1%) and frequently (CNA frequency >= 1%) mutated genes. The chromosomal distances of rare and frequent gene CNAs from known essential genes, fragile sites, oncogenes, tumor suppressor genes, and known frequently observed CNV sites in human germ cells were computed and visualized by cohort-specific boxplots. See Tab. S8-10 for known genomic features. The distance distributions are highly tumor-type-specific. Significant differences in distances to known genomic features between rarely and frequently gene CNAs are represented by '*' (p-value < 0.05) and '**' (p-value < 0.01) based on Wilcoxon tests.

2.22 Figure S22: Kaplan-Meier analyses

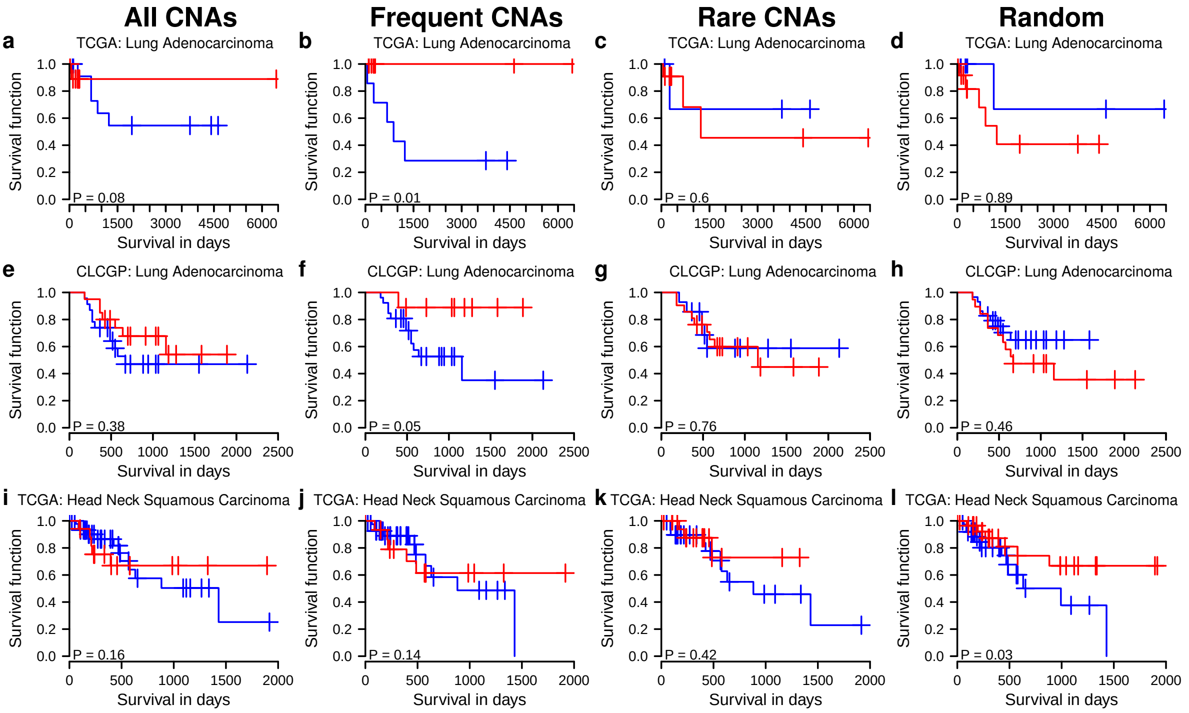

Figure S22: Impact of rare and frequent patient-specific gene CNAs on survival for lung adenocarcinoma and head neck squamous cell carcinoma patients. Kaplan-Meier curves for patients with negative (blue) and positive (red) average impact of their corresponding tumor-specific gene CNAs on cancer type-specific survival signature genes. Shown are results for independent tumor cohorts (TCGA: new lung adenocarcinoma patients (LUAD), CLCGP: lung adenocarcinoma (LUAD), TCGA: new head and neck squamous cell carcinoma patients (HNSC)) that were not involved in any step of CCTN network inference or RF-based prediction of survival signature genes. CCTN derived from cancer cell lines and cancer type-specific survival signature genes identified from TCGA cohorts were used to investigate the impact of rare and frequent gene CNAs on patient survival for these cohorts. Grouping of patients into short and long survival by CCTN is expected to be predictive if patients with positive average survival impact scores (red) tend to survive longer than patients with negative impact scores (blue). The corresponding p-value quantifies if the red curve is significantly above the blue curve in comparison to randomly permuted class labels. **a – d,** TCGA LUAD results. **a,** Patients with positive average impact scores on survival signature genes (red) tend to survive significantly longer than patients with negative impact scores (blue). **b,** Frequent patient-specific gene CNAs (frequency $>=$ 1% in corresponding training cohort) can explain the significant difference in panel a. **c,** Rare patient-specific gene CNAs (frequency $<$ 1% in corresponding training cohort) do not significantly contribute to the observed significant differences in panel a. **d,** Loss of patient separation into short and long survival for patient-specific survival impact scores computed based on all patient-specific gene CNAs under a random network. **e – f,** CLCGP LUAD results. Very similar behavior as observed for the independent analysis of new TCGA LUAD patients. **i – l,** TCGA HNSC results. Patient-specific frequent and rare gene CNAs both contribute to a discrimination between patients with short and long survival, but the overall separation is not better than under a random network.

2.23 Figure S23: Kaplan-Meier analyses comparing CCTN to random networks

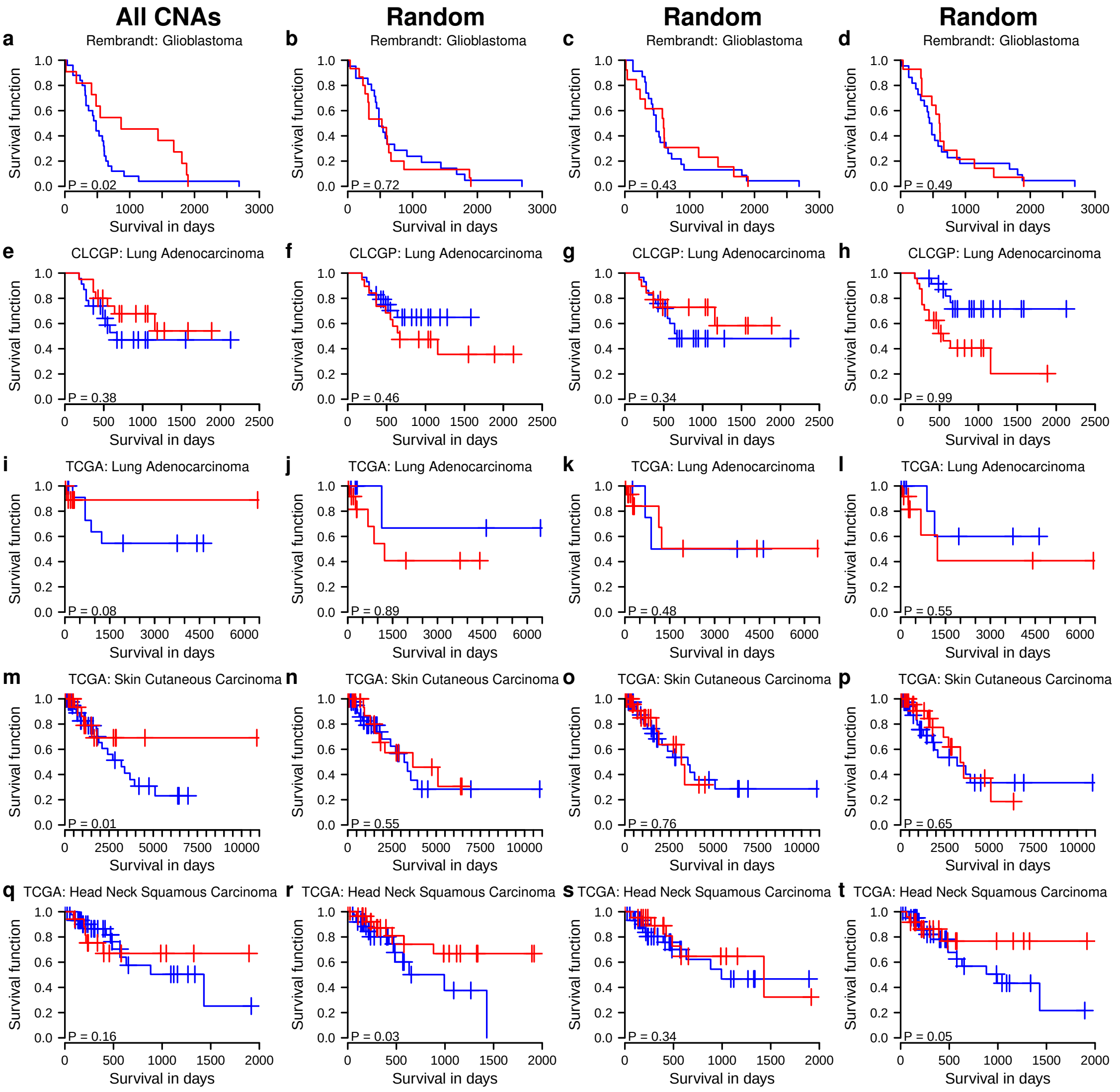

Figure S23: Comparison of CCTN-based Kaplan-Meier analyses to results obtained under random networks. Kaplan-Meier curves for patients with negative (blue) and positive (red) average impact of their corresponding tumor-specific gene CNAs on cancer type-specific survival signature genes. Shown are results for independent tumor cohorts (Rembrandt: Glioblastoma (GBM), CLCGP: Lung adenocarcinoma (LUAD), TCGA: new lung adenocarcinoma patients (LUAD), TCGA: Skin cutaneous carcinoma new patients (SKCM), TCGA: new head and neck squamous cell carcinoma patients (HNSC)) that were not involved in any step of CCTN network inference or RF-based prediction of survival signature genes. CCTN derived from cancer cell lines and cancer type-specific survival signature genes identified from TCGA cohorts were used to investigate the impact of tumor-specific gene CNAs of each patient on survival. Separation of patients into short and long survival by CCTN is expected to be predictive if patients with positive average survival impact scores (red) tend to survive longer than patients with negative impact scores (blue). The corresponding p-value quantifies if the red curve is significantly above the blue curve in comparison to randomly permuted class labels. Comparisons to results obtained using random networks of the same complexity as CCTN (degree-preserving permutations) provide additional evidence for the relevance of predictions. The leftmost column shows the discrimination between patients that were classified by CCTN into long (red curve) and short (blue curve) survival. These expected discrimination is widely lost using random networks of the same complexity as CCTN for patient-specific impact computations except for head neck squamous carcinoma (see columns with label 'Random'). This indicates that CCTN contains relevant information to predict impacts of tumor-specific gene CNAs on tumor type-specific survival signature genes for the separation into short and long-lived patients.

2.24 Figure S24: CCTN p-value and q-value distributions

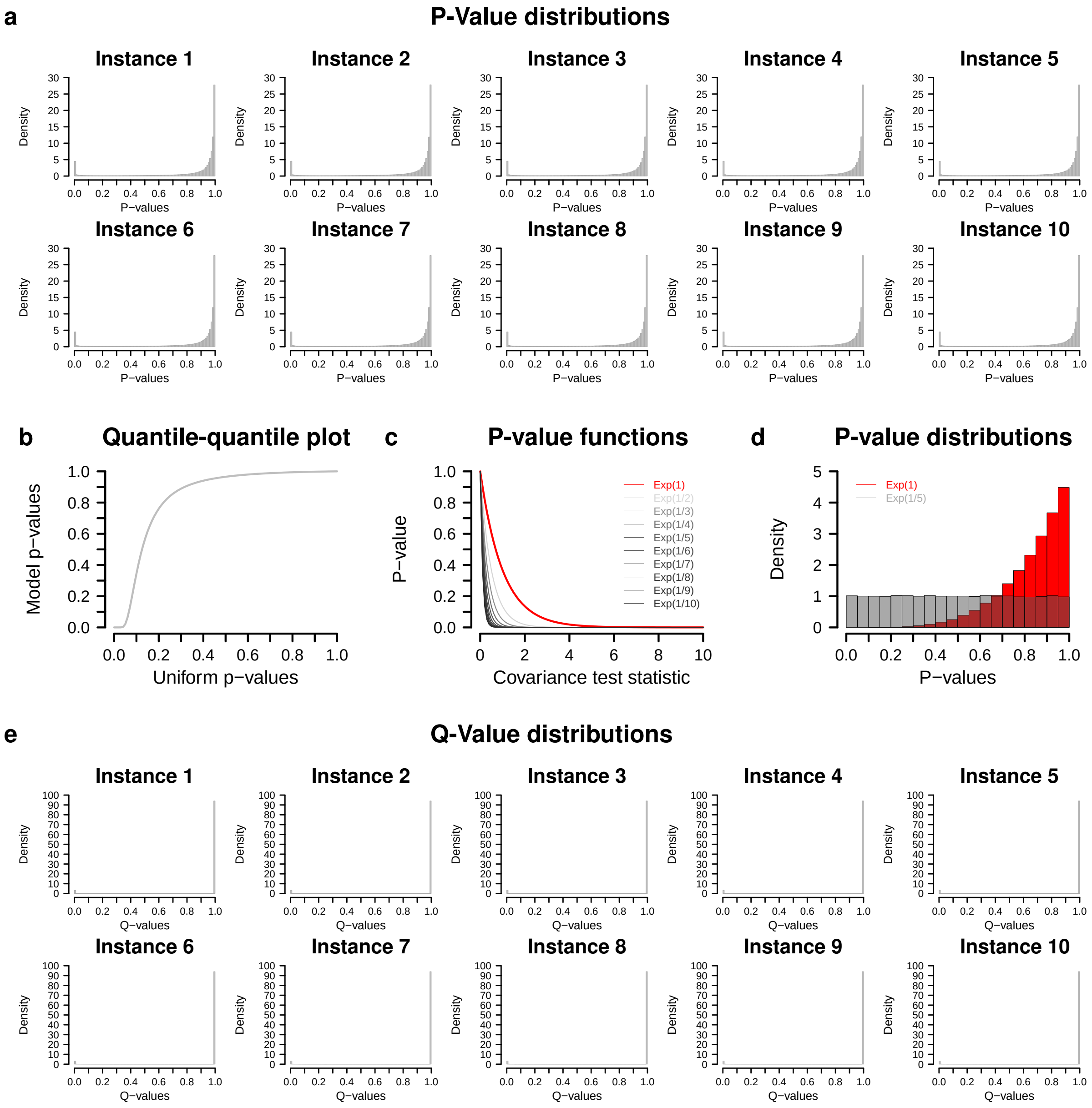

Figure S24: a, P-value distributions of active CCTN predictors that were selected based on lasso for ten different CCTN instances. All p-value distributions show a strong enrichment of non-significant p-values close to one and a smaller peak for highly significant p-values with values close to zero. P-values in between these two extremes tend to be uniformly distributed. As one would expect for lasso-based network inference, only very few predictor genes were selected as predictors for the prediction of the expression levels of specific response genes (small p-values close to zero), whereas the majority of predictors is not relevant (p-values clearly greater than zero or close to one). Considering CCTN in our analysis, we only focused on those predictors that had p-values $< 5 \cdot 10^{-5}$ (standard numerical precision limit of the R package covTest defining the smallest p-value that is provided by this package before p-values are rounded to zero by covTest) in all ten CCTN instances. **b,** Quantile-quantile plot comparing the pooled p-value distribution of the ten CCTN instances against a uniform distribution. The enrichment of model p-values close to one is explained by the theory behind the covariance test statistic by Lockhart et al. (2014). Comparisons of the test statistic against an exponential distribution Exp(1) with scale parameter one along the lasso path are increasingly conservative leading to an enrichment of greater p-values as illustrated in c and d. **c,** P-value functions for the covariance test statistic by Lockhart et al. (2014). The underlying covariance test statistic used to determine the p-values asymptotically follows an exponential distribution Exp(1) with scale parameter one

(red). In more detail, consider a set of N predictors of which the first predictors $\{1, \ldots, n\}$ are the truly active ones, whereas the remaining predictors $\{n+1, \ldots, N\}$ are the truly non-active ones. It was shown in Lockhart et al. (2014) that the covariance test statistic of each truly active predictor follows an Exp(1), whereas the test statistic of each truly non-active predictor $i \in \{n+1, \ldots, N\}$ follows an Exp(s) with scale parameter $s := 1/i$. Because truly active and non-active predictors are generally not known, comparisons of the covariance test statistic along the lasso path to an Exp(1) (red) are done. These comparisons are increasingly conservative. This is shown by the fact that Exp(1) is providing greater p-values for the same value of the covariance test statistic than an Exp(s) with scale parameter $0 < s < 1$. This increasing conservatism leads to a left-skewed p-value distribution as illustrated in d. This also explains the enrichment of p-values close to one in a and b. **d,** P-value distributions for randomly drawn test statistic values from Exp($1/5$) under consideration of Exp(1) and Exp($1/5$) for the corresponding p-value computations. P-values belonging to the truly underlying Exp($1/5$) are uniformly distributed. Corresponding p-values computed using the Exp(1) (because it is generally not known if a predictor belongs to the truly active ones) show the characteristic left skew. This is expected from the theory behind the significance test for lasso by Lockhart et al. (2014). **e,** Corresponding q-value distributions of active CCTN predictors that were selected based on lasso for ten different CCTN instances. Q-values were computed using the R function p.adjust. All active predictors that had very small p-values also had very small q-values.

2.25 Figure S25: Influence of local chromosomal predictors

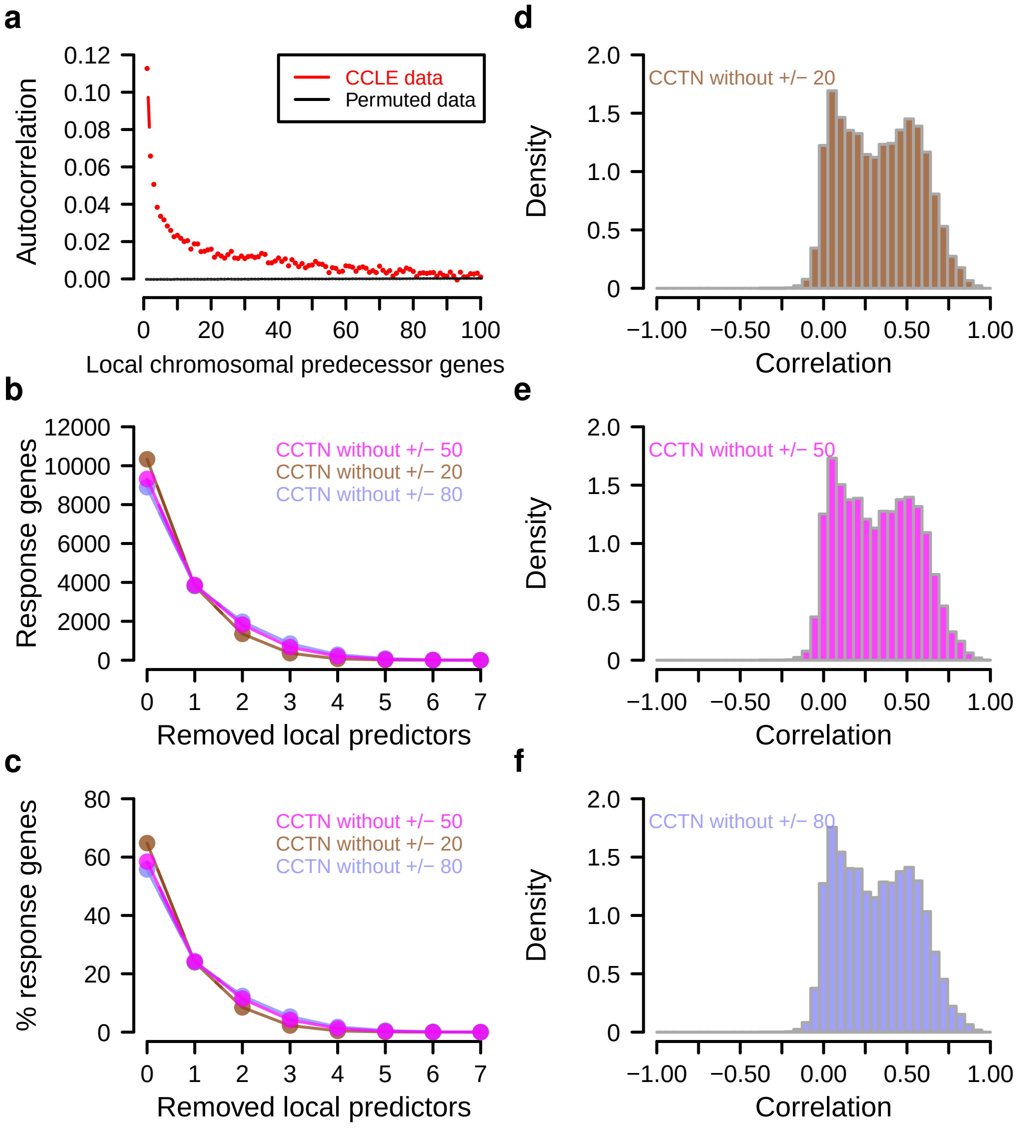

Figure S25: Influence of potential local chromosomal predictor genes on the prediction of CCLE gene expression levels. **a,** Local chromosomal correlations of expression levels of genes in close chromosomal proximity considering the CCLE data set. The red curve represents the average autocorrelation function computed based on the individual autocorrelation functions that were obtained for each chromosome-specific expression profile of each of the 768 CCLE cell lines. The autocorrelation function quantifies the similarity of expression levels of neighboring gene pairs on a chromosome under consideration of an increasing number of genes located in between both genes of a gene pair. Local chromosomal correlations of expression levels between neighboring genes die out fast towards zero for an increasing distance of genes (red curve). Local chromosomal correlations are completely lost considering randomly permuted chromosome-specific CCLE expression profiles. **b - c,** Count statistics summarizing the number/percentage of response genes that were unaffected (removed local predictors equal to zero) or affected (removed local predictors greater than zero) by the removement of local chromosomal predictors twenty, fifty or eighty genes up and downstream of each response gene in CCTN. The majority of response genes (about 58%) did not have any local chromosomal predictor. For about 24% of response genes one local chromosomal predictor was removed. Two local chromosomal predictors were removed for about 11% of the response genes. Only very few response genes had more than three local predictors. Overall the curves show that the removement of local predictors is very robust considering different local gene predictor cutoffs. **d - f,** CCTN-based prediction of gene expression levels for the considered 13 TCGA cohorts (median gene-specific correlations between predicted and originally measured expression levels) is nearly unaffected by the choice of different local gene predictor cutoffs.

2.26 Figure S26: CCTN variance inflation factors

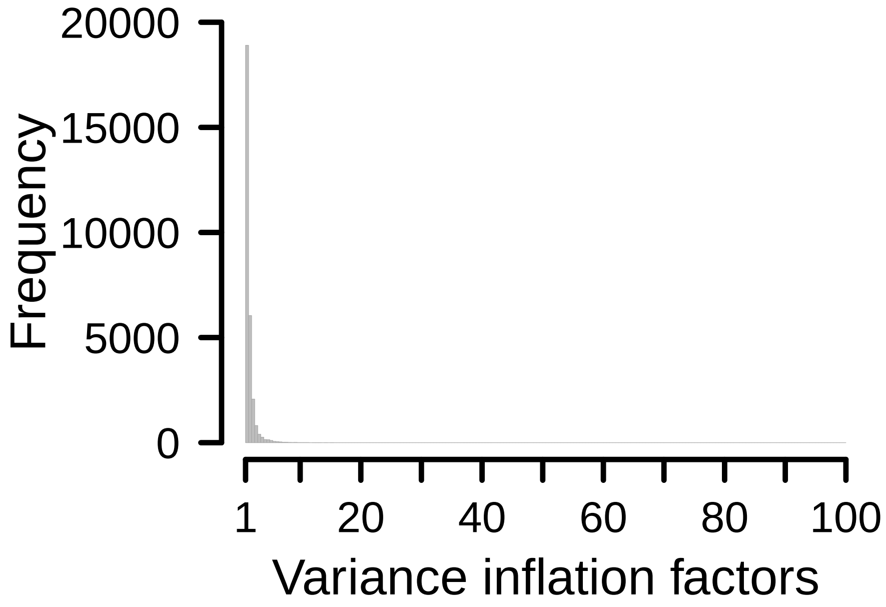

Figure S26: Histogram of variance inflation factors for predictors (regulator genes) of CCTN response genes (target genes). The variance inflation factor is a measure to quantify the correlation of predictors that were selected for a specific response gene based on a linear regression approach. For each response gene with at least two predictors one determines for each subset of predictors $S \setminus \{i\}$ how good they can explain the expression level of each predictor $\{i\}$. This is done via standard linear regression and one defines the corresponding variance inflation factor as $1/(1 - R_i^2)$, where R_i^2 is the coefficient of determination of the corresponding linear model. In the literature, a variance inflation factor greater than ten is indicating a high collinearity between predictors Kutner et al. (2004). Lasso-regression in combination with the significance test is expected to account for the collinearity of response gene-specific predictors by selecting only those predictors that best explain the expression of the response gene. For CCTN this is done by lasso-based shrinkage of individual predictor parameters toward zero to remove irrelevant predictors and by an additional selection of predictors with highly significant p-values. The histogram clearly shows that the vast majority of variance inflation factors is close to one. Only 0.16% of all variance inflation factors were greater than ten. Thus, as one would expect for lasso approach, CCTN accounts for collinearity of predictors.

2.27 Figure S27: Robustness of CCTN network inference

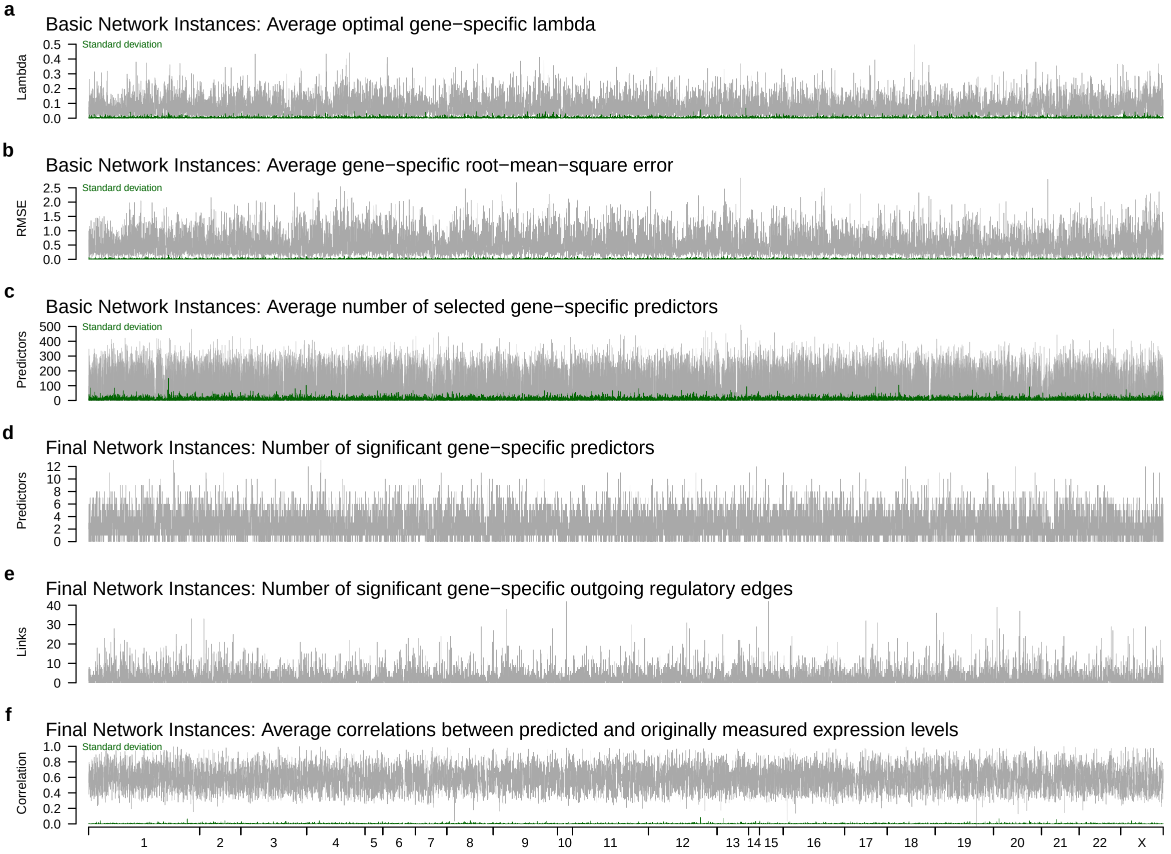

Figure S27: Evaluation of the stability of the developed lasso-based network inference approach. The inference of CCTN from the CCLE data set was repeated ten times. The 15,942 genes of the CCLE data set are represented from chromosome 1 to X. **a - c,** Summary for basic CCTN network instances. **a,** Average optimal gene-specific complexity parameter λ_i (grey) and corresponding standard deviations (green). **b,** Average gene-specific root-mean-square error (grey) and corresponding standard deviations (green). **c,** Average number of selected gene-specific predictors (grey) and corresponding standard deviations (green). **d - f,** Summary for final CCTN network instances only focusing on relevant predictors. **d,** Number of gene-specific predictors in the final CCTN network. Only relevant gene-specific predictors (gene copy number, regulator genes at least fifty genes up or downstream of each target gene) with a p-value < $5 \cdot 10^{-5}$ in all ten CCTN network instances were included. **e,** Number of outgoing gene-specific regulatory edges in the final CCTN network. **f,** CCTN-prediction accuracy of the CCLE data set across the ten final CCTN network instances.

2.28 Figure S28: Patient-specific vs. cohort-specific impact scores

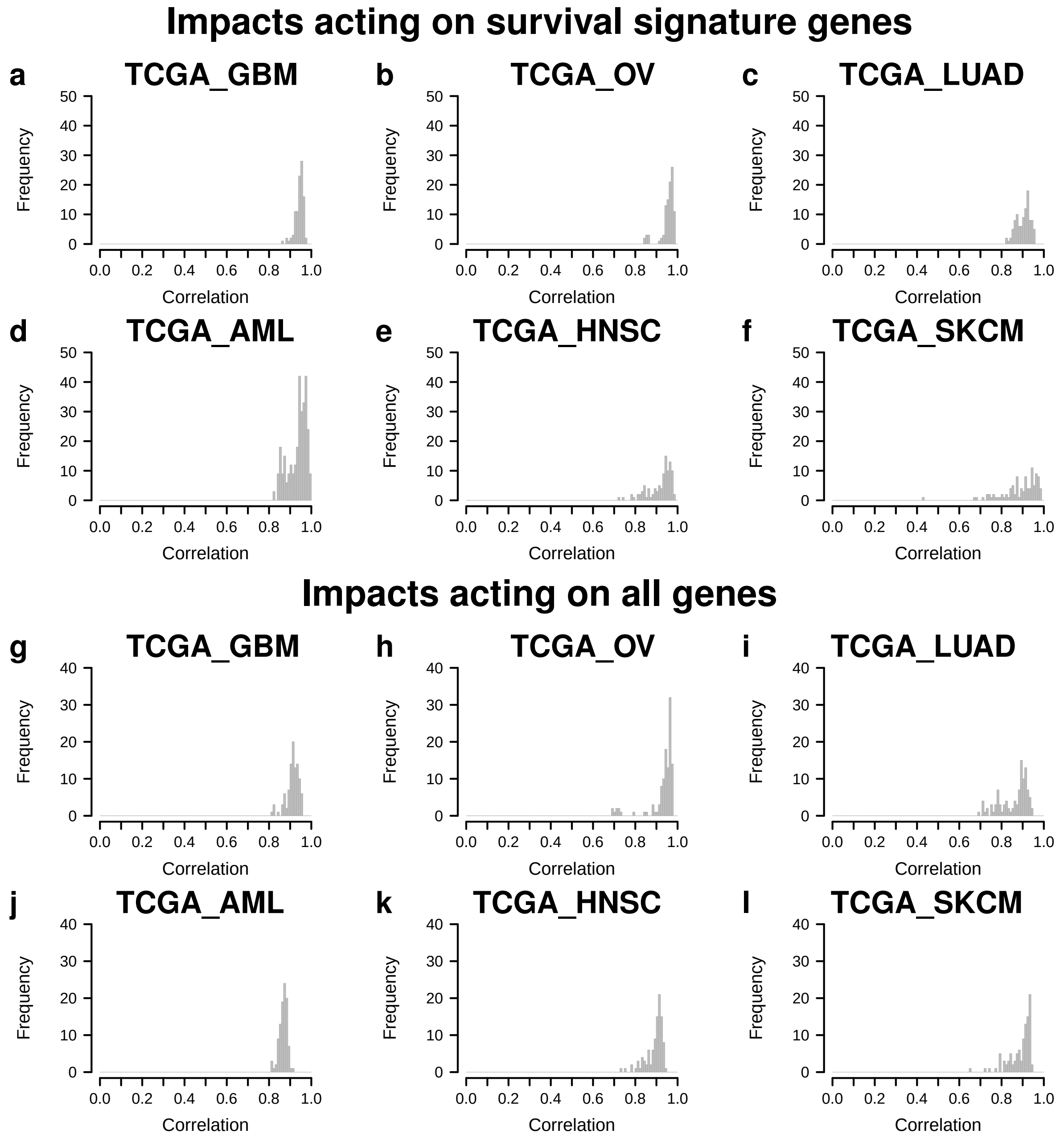

Figure S28: Comparison of patient-specific to cohort-specific impact scores for six TCGA cohorts. Patient-specific and cohort-specific impact matrices were computed by our network propagation algorithm with respect to CCTN. See methods in the main manuscript and Text S1 for details. The resulting impact matrices enable to quantify the direct and indirect impact of each specific gene on the expression of an another specific gene. Since these computations are extremely time and resource consuming, we randomly selected 100 tumor patients from each TCGA cohort for the computation of patient-specific impact scores. **a – f,** Correlations between all patient-specific impact values and corresponding cohort-specific impact values focusing on cohort-specific survival signature genes. **g – l,** Correlations between all patient-specific impact values and corresponding cohort-specific impact values considering all 15,942 $\times$ 15,942 impact values for genes included in CCTN.

References

Belcastro, V., Siciliano, V., Gregoretti, F., Mithbaokar, P., Dharmalingam, G., Berlingieri, S., Iorio, F., Oliva, G., Polishchuck, R., Brunetti-Pierri, N., and di Bernardo, D. (2011). Transcriptional gene network inference from a massive dataset elucidates transcriptome organization and gene function. *Nucleic Acids Res*, 39(20):8677–8688.

CLCGP (2013). Clinical Lung Cancer Genome Project (CLCGP) and Network Genomic Medicine (NGM): A genomics-based classification of human lung tumors. *Sci. Transl. Med.*, 5(209):209ra153.

Kutner, M. H., Nachtsheim, C. J., and Neter, J. (2004). *Applied Linear Regression Models*, volume 4. McGraw-Hill Education.

Lockhart, R., Taylor, J., Tibshirani, R. J., and Tibshirani, R. (2014). A significance test for the lasso. *Ann. Stat.*, 42(2):413–468.

Madhavan, S., Zenklusen, J.-C., Kotliarov, Y., Sahmi, H., Fine, H. A., and Buetow, K. (2009). Rembrandt: Helping Personalized Medicine Become a Reality Through Integrative Translational Research. *Mol. Cancer Res.*, 7(2):157–167.

Seifert, M., Abou-El-Ardat, K., Friedrich, B., Klink, B., and Deutsch, A. (2014). Autoregressive Higher-Order Hidden Markov Models: Exploiting Local Chromosomal Dependencies in the Analysis of Tumor Expression Profiles. *PLoS ONE*, 9(6):e100295.
